# Stereochemical Analysis of Natural Products: Bisindole Alkaloids of the *Strychnos-Strychnos* Type

**DOI:** 10.3390/ijms27010337

**Published:** 2025-12-28

**Authors:** Dmitry A. Grigoriev, Valentin A. Semenov, Luc Angenot, Leonid B. Krivdin

**Affiliations:** 1 A. E. Favorsky Irkutsk Institute of Chemistry, Siberian Branch of the Russian Academy of Sciences, Favorsky St. 1, 664033 Irkutsk, Russia; 2 Laboratory of Pharmacognosy, Center of Interdisciplinary Research on Medicines, Faculty of Medicine, University of Liège, 4000 Liège, Belgium; l.angenot@uliege.be

**Keywords:** monomeric and dimeric indole alkaloids of *Strychnos*-type, stereochemistry, conformational analysis, ^1^H and ^13^C NMR computational NMR, DFT calculations

## Abstract

Results of the high-level computational study of 12 bisindole alkaloids of the *Strychnos-Strychnos* type are reported in addition of that of five retuline-like monomers frequently encountered in moieties constituting dimeric alkaloids. Based on the comparison of theoretical and experimental NMR chemical shifts, a detailed conformational survey of these stereochemically rich natural products containing multiple asymmetric centers was performed. Our original methodology is based on searching multiple conformational states, geometry optimization, and high-level NMR calculations at the DFT level. Taking into account all known experimental chemical shifts together with their calculated values in the natural products under study, a correlation estimate of NMR chemical shifts was performed by using statistical descriptors. In general, a good agreement of the performed calculations with experiment was achieved, which is manifested by the Corrected Mean Absolute Error of about 0.2 ppm for ^1^H and 1.9 ppm for ^13^C NMR chemical shifts in this series.

## 1. Introduction

It is well known that NMR spectroscopy provides a powerful and versatile tool for the elucidation and verification of chemical structure of natural products. The development of computational NMR opens up new possibilities in this field. Quantum-chemical calculations of NMR chemical shifts are intended to clarify the reliable assignment of NMR experimental data of mono- and bisindole alkaloids, which is demonstrated in the present paper for 17 representatives of the *Strychnos-Strychnos* family. Total synthesis of natural products is one of the most challenging and exciting areas of organic and bioorganic chemistry, imitating nature’s ability to create diverse molecular structures. At that, the development of complex multistage syntheses is associated with significant material and time costs. Another peculiarity deals with the fact that even in the present “golden age” of NMR, it is far from being uncommon to establish the structure of the erroneously identified newly isolated natural products, resulting in numerous revisions and reestablishments of the originally proposed structures, as observed for the studied series of compounds.

At present, we are witnessing an unprecedentedly fast development of theoretical and computational methods in the field of NMR spectroscopy [1,2,3,4,5] in parallel with a marked progress in experimental NMR. As a result, an impressive breakthrough of the DFT methods, which are among the most efficient and economic computational tools for the stereochemical analysis of natural products and biological macromolecules, is taking place. In fact, all contemporary computational efforts are aimed at increasing the accuracy of quantum chemical calculations of NMR parameters by taking into account the choice between the cost and the quality of the performed calculations. On the other hand, the usage of the ab initio wavefunction-based correlated methods based on the polarization propagator approach and/or coupled-cluster theories is becoming more and more popular but is not yet used routinely in the computational NMR of natural products. Notwithstanding all its power, NMR experiment, either conducted alone or combined with other experimental techniques, may sometimes give results that are hard to interpret to make an unambiguous conclusion on the chemical structure of a particular natural product. In particular, such a situation may occur when it is necessary to determine which of the very close relative structures provide the observed NMR signals in the usual or unexpected ranges. Whenever such a situation occurs, quantum chemical calculations may be of essential help in resolving the problem. In general, computation of NMR chemical shifts provides a new guide in the understanding of the fundamental factors controlling stereochemistry and chemical reactivity of a variety of natural products and bioorganic molecules, being on the cutting edge of modern computational chemistry.

At the same time, it is somewhat difficult to identify the stereochemical structure of natural products by means of NMR, which is because they have several asymmetric centers. Calculations of NMR chemical shifts of natural products like alkaloids with multiple asymmetric centers are performed nowadays mostly within the DFT framework, in contrast to the non-empirical computations applied to much smaller molecules.

It should also be emphasized that at the DFT level, electron correlation is involved in an implicit way, so that such calculations are much more economical, as compared to the non-empirical methods, the latter taking into account electron correlation explicitly. From this point of view, to date, many different approaches to the existing methods have been developed to improve the accuracy of calculations within the DFT formalism. A number of approaches based on determining the probability distribution of a set of stereoisomers, like that of the DP4 family of methods, are widely utilized in stereochemical analysis of complex natural products, being much more robust as compared to classical statistical and all the more so computational methods [6,7,8,9,10]. Among other approaches, machine learning methods should also be mentioned, which are extensively used in the calculations of NMR chemical shifts [11,12]. However, it should be kept in mind that the results of the DFT calculations drastically depend on the choice of a particular functional. In this study, all calculations of ^1^H and ^13^C NMR isotropic magnetic shielding constants (and, accordingly, chemical shifts) were carried out at the DFT level in the liquid phase, with the most reliable for this purpose PBE0 functional being compared to available experiment.

**Scheme 1:**
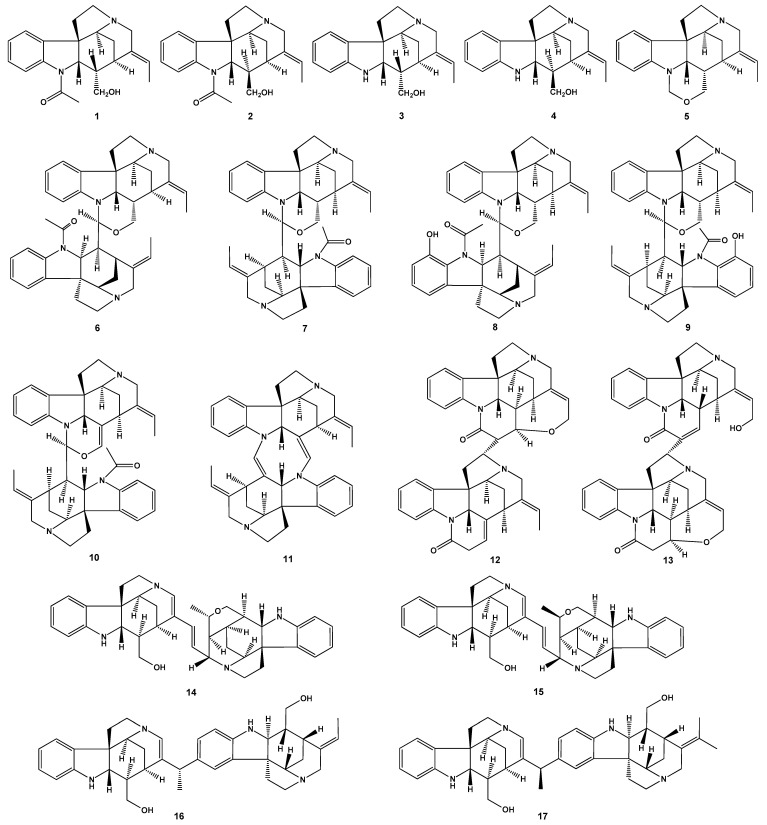
Structures of the studied mono- and bisindole alkaloids of the *Strychnos*-type **1**–**17**.

As already mentioned, this study continues a series of papers devoted to the stereochemical analysis of natural bisindole alkaloids [13,14,15]. In this paper, based on the correlation of theoretical and experimental NMR chemical shifts, we perform a detailed conformational survey of the known alkaloids of *Strychnos-Strychnos* type shown in Scheme 1. These dimers are frequently constituted of moieties belonging to the retuline and isoretuline series whose ^13^C NMR data have only been partially published.

The first bisindole alkaloids (*diquaternary* and *ditertiary*) were discovered in Calabash-curare after intensive research for about thirty years in the UK, Germany, and Switzerland. Calabash curare, as well as the barks of *Strychnos toxifera*, contained several dozen alkaloids very difficult to separate. Toxiferine I, 18,18′-dideoxy- (synonym of *C*-dihydrotoxiferine) was one of the first *quaternary* to be isolated in 1941, but it has been misnamed because no formal relation to toxiferine I (later isolated) was demonstrated to justify the prefix “di-hydro” [16,17,18]. Toxiferine I, 18,18′-dideoxy- was the first bisindole *Strychnos* alkaloid to be structurally elucidated through an outstanding series of studies involving, in particular, synthesis from Wieland-Gumlich aldehyde [19]. Finally, the arrangement of the double bonds in the central portion of the molecule has been deduced and established by NMR studies [20,21]. Dihydrotoxiferine is thus a *diquaternary* symmetrical bisindole alkaloid with strong muscle-relaxant (curarizing) properties.

However, the most encountered symmetrical dimer is the bis-nor parent of the former alkaloid that has lost two methyl groups located on the basic nitrogens N-4 and N-4′as shown in the structure of **11** (Scheme 1). It was discovered in 1955 and called bisnordihydrotoxiferine as its methylation yielded the parent [22]. Later, complete NMR data were published in 1997 [23]. This alkaloid could also be named “bisdesmethyl 4,4′- 18,18′-dideoxy-toxiferine 1” instead of bisnordihydrotoxiferine, which has been used in all phytochemical papers. This symmetrical alkaloid represents a self-condensation product of 18-desoxy-Wieland-Gumlich aldehyde that could also be named as deacetylisoretulinal. All these molecules are strictly symmetrical and linked by two bonds between N-1 and C-17′ on the one hand, and between C-17 and N-1′ on the other hand. Their NMR spectra are much easier to analyze than unsymmetrical dimers because the signals are divided into two groups. Some new symmetrical *tertiary* dimers, particularly, matopensine and derivatives, have also been isolated [24].

**Figure 1:**
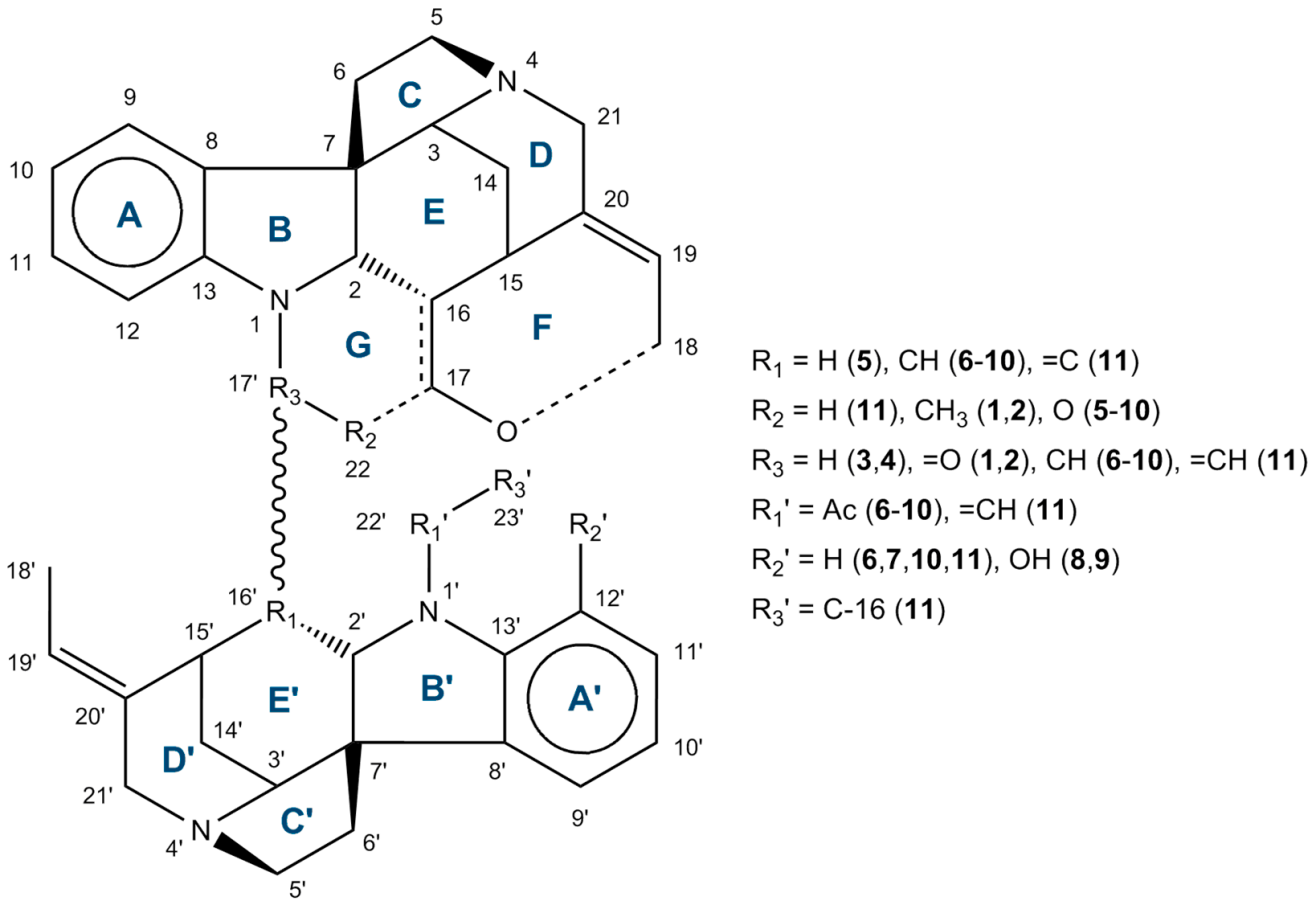
General layout of rings and numbering of the *Strychnos*-type alkaloids **1**–**11**.

The other bisindole *Strychnos-Strychnos* type alkaloids found in the *Strychnos genus* are all unsymmetrical. They are classified into four subgroups:

(1) The first subgroup is represented by the strychnobilines, whose first two have been isolated in 1978 from the rootbarks of *Strychnos variabilis*, an African species growing in Central Africa, where it is said to be poisonous. This type of dimers linked by one junction between N-1 and C-17′ presents thus two parts: the upper one has six rings (five for deacetyl-retuline portion plus one oxazinic portion), and the lower part has five rings (deacetyl(iso)retuline product)—see layout in Figure 1. This subgroup of bisindole alkaloids has the priority for study because the ^13^C NMR data are missing [25,26,27,28].

(2) The second subgroup was discovered in 1979, when sungucine was identified from *Strychnos icaja* roots, which are used as arrow and ordeal poison [29]. Later, other alkaloids of this group, such as isosungucine and strychnogucines, were isolated [30,31]. All of them result from both strychnane moieties (6 or 7 rings) linked by a single bond between C-23 and C-5′. For these alkaloids, there are also X-ray experiments available [32].

(3) The third subgroup is constituted from panganensines isolated in 1996 from the African *Strychnos panganensis*. They are also formed by the attachment of two strychnane units—retuline, isoretuline, and spermostrychnine skeletons. The formation of one bond between C-21′ and C-18 has been described for panganensines R and S, although the junction between C-19 and C-10′ was proposed in the case of panganensines X and Y. The ^1^H and ^13^C NMR data were described by Nuzillard et al. [33].

(4) The fourth subgroup was described in 2014 during the structural identification of strychnobaillonine, which was also isolated from *Strychnos icaja* roots. This unsymmetrical alkaloid shows two junctions: one including C-17–N-1′ like symmetrical dimers such as bisnordihydrotoxiferine on the one hand, and a second one between C-23 and C-17′, on the other hand. These two bonds allow the union of a retuline moiety (upper part) and N-1 of deacetylisoretuline (lower part) [34]. There is a very good correlation between the experimental and calculated ^1^H and ^13^C NMR chemical shift data [35].

## 2. Results and Discussion

In the present study, we performed a stereochemical survey of 17 alkaloids of the *Strychnos*-type by using our previously proposed [36] simple and versatile methodology for the stereochemical study of the natural products containing multiple asymmetric centers, which is based on a thorough analysis of the computed NMR chemical shifts.

**Figure 2:**
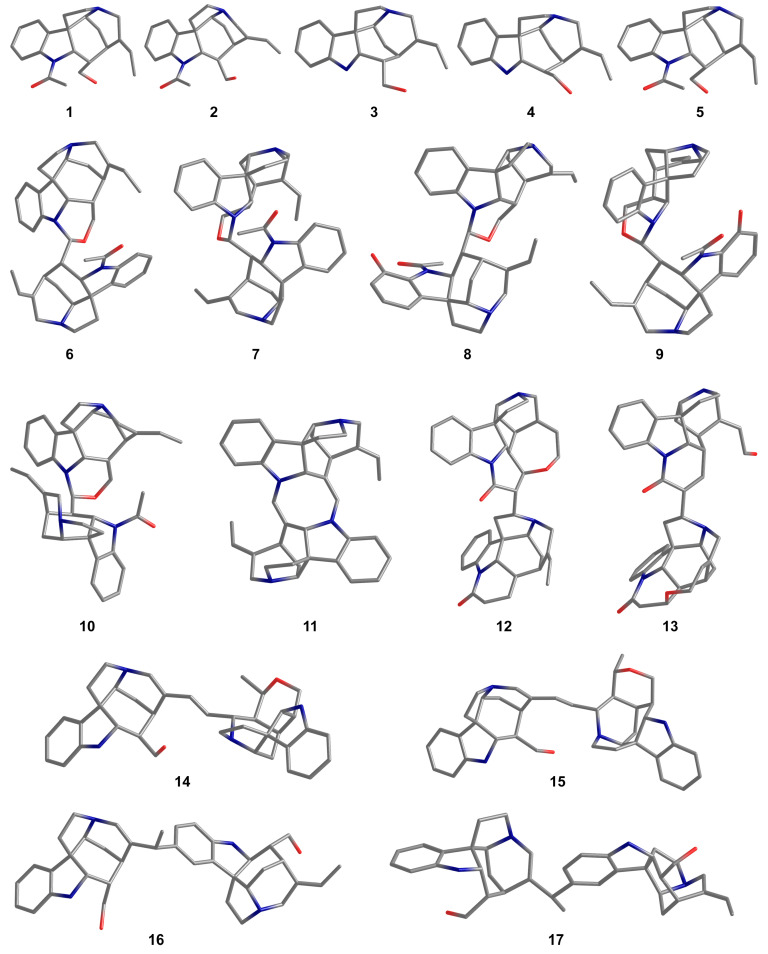
The spatial structures of compounds **1**–**17** optimized at the M06-2X/pecG-2 level. Hydrogen atoms are omitted for clarity.

At the initial stage of this study, a primary conformational search was conducted for 17 selected strychnan alkaloids **1**–**17**, followed by the optimization of their geometric parameters. The spatial structures of compounds **1**–**17** optimized at the M06-2X/pecG-2 level are shown in Figure 2. For the optimized structures, the shielding constants and corresponding ^1^H and ^13^C NMR chemical shifts were calculated within the GIAO-DFT theory using the PBE0 functional in combination with our original pecS-2 basis set developed specifically for the calculation of NMR chemical shifts. A general methodology for searching conformational states, geometry optimization, and NMR calculations is described in more detail in Section 3. Materials and Methods.

**Figure 3:**
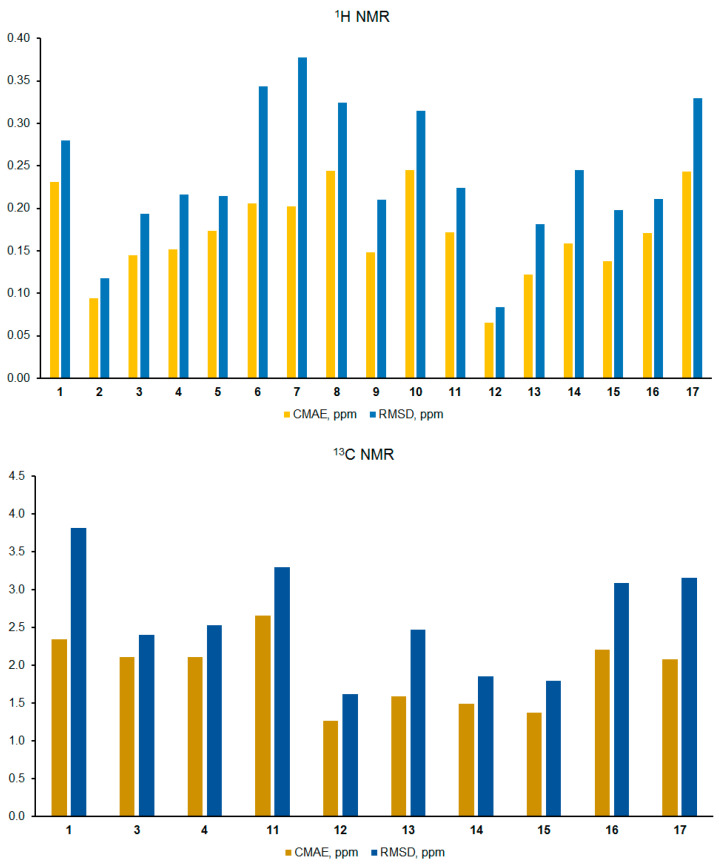
Results of the performed correlation analysis of calculated versus experimental NMR chemical shifts in the series of *Strychnos*-type alkaloids **1**–**17**.

Since a conformational search was initially performed for each of the studied alkaloids **1**–**17**, only the most energetically stable ones were selected for further investigation of the stereochemical features (excluding “special” cases of C-G rings conformations, which will be discussed below). It is known that the NMR spectra of solutions contain averaged signals of the dynamic exchange of possible present conformers. Therefore, as a rule, when calculating NMR chemical shifts, Boltzmann-weighted shielding constants are used, with their own specific coefficients depending on their free energy. However, in the present study, we did not perform Boltzmann averaging, since the distribution of conformers of the studied bisindole alkaloids mainly represents the distribution of rotamers along the C-16′–C-17′ bond—see layout in Figure 1. The rotation of two subunits along this bond has a fairly significant barrier, up to 15 kcal/mol. Thus, the criterion for determining the most probable conformer/rotamer was only the correlation between the calculated and experimental NMR chemical shifts, but not the thermodynamic criterion. For further discussion, only those alkaloid rotamers with the best parameters of the statistical descriptors used were considered—see Figure 3.

Taking into account all known experimental chemical shifts together with their calculated values, a correlation estimate of NMR chemical shifts was performed using such statistical descriptors as Corrected Mean Absolute Error (CMAE) and Root Mean Square Deviation (RMSD)—see Figure 3. In general, a good agreement with experimental data was obtained, which is manifested by CMAE of about 0.2 ppm for ^1^H and 1.9 ppm for ^13^C NMR chemical shifts; for ^1^H NMR chemical shifts, the integral RMSD value is in the range of 0.08–0.37 ppm, while in the case of ^13^C NMR, it was found to be in the range of 1.6–3.8 ppm.

As can be seen from the ^1^H NMR data presented in this diagram, the highest correlation coefficients were obtained for strychnogucine A (**12**) and isoretuline (**2**) with CMAE of 0.06–0.09 ppm. The largest discrepancy between calculated values and experiment was observed for 12′-hydroxystrychnobiline (**8**) and 16,17-dehydroisostrychnobiline (**10**). Below, we will examine in detail and try to explain the reasons for such deviations.

Overall, stereochemical modeling of compounds **1**–**17** confirmed predicted configurations of their asymmetric centers. However, there are several ambiguous cases where exclusively large deviations of calculated NMR chemical shifts from their experimental counterparts were observed. A detailed listing of all such cases is presented in Table 1.

In a number of cases, the performed study provided unambiguous assignments of the ^1^H NMR multiplets of the strongly coupled spin systems of fused rings (see layout, Figure 1), which were not explicitly assigned in the original publications. In some particular cases, we performed reassignments of the individual resonances together with experimentally unresolved signals. More detailed comments on each compound in the studied series of alkaloids are given below.

For this conformational study, five monomeric indolinic alkaloids were first analyzed because they present the characteristics of *Strychnos* type: at least a pentacyclic scaffold (rings A–E) with a morphan core (rings D, E) as the central segment surrounded by fusing rings (A–C) and sometimes G and/or F rings. We did not select the isomeric pair of aldehydic alkaloids (retulinal/isoretulinal) that were the main monomeric tertiary alkaloids found in the rootbarks of *Strychnos variabilis* because their spectra are very complex. Indeed, these alkaloids show an epimerization at the level of proton 16 and a rotamery about the acetamide function, with an equilibrium between the two forms of each epimer that will vary according to the solvent and the temperature. Consequently, these spectra correspond to the analysis of four substances in variable proportions [37].

**Retuline** (**1**). This natural product, extracted from many species of the *Strychnos* genus, including *S. variabilis*, is an important representative of monoindole alkaloids that play a significant role in forming the architecture of the *Strychnos-Strychnos* bisindole alkaloids, including this subunit. To construct an adequate structural model of corresponding bisindole alkaloids, a thorough study of their individual subunits is necessary. A crucial point in this task is the determination of the unique stereochemical features and possible configurational deviations of this group of alkaloids.

A comparison of the empirical results with theoretical calculations revealed a satisfactory correlation. However, several found discrepancies deserve special attention. For example, the deviation from theoretical value of the resonance signal for one of the methylene protons at position 21 significantly exceeds the overall deviation variance. Also, an examination of ^13^C NMR data allowed identifying three key carbon atom positions providing large deviations: C-8, C-19, and C-20 (see Table 1).

Theoretical stereoelectronic analysis of retuline (**1**) suggests a downfield shift of the C-8 resonance signal due to the mesomeric effect of the acetyl group on the cyclic nitrogen atom N-1. However, the experimentally observed chemical shift of C-8 is in the opposite direction, which is explained by the less-than-expected strong influence of the electron-withdrawing acetyl group, which provides a complex multifactorial effect of enhanced shielding of the C-8 atom, which cannot be adequately reproduced by DFT. On the contrary, deviations of C-19 and C-20 correspond to the expected trend, demonstrating a pronounced influence of the donor character of the adjacent CH_3_-18.

**Figure 4:**
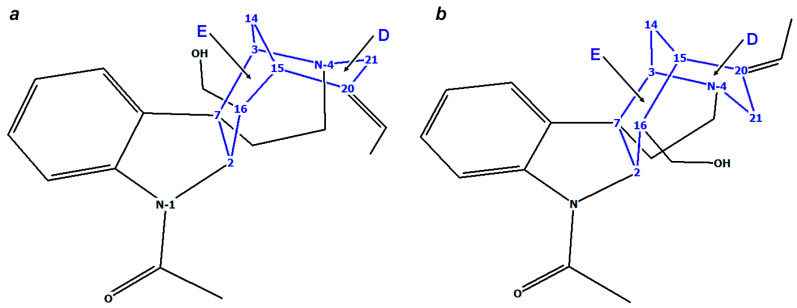
Stereochemical features of rings E and D of retuline **1** (**a**) and isoretuline **2** (**b**).

According to the early studies by Tavernier et al. [38], retuline, like strychnine, has a *boat* conformation of ring D, but with an inversion of the configuration at C-16—see Figure 4a. This is confirmed by the parameters of the long-range interaction between H_β_-5 and H_β_-21 (see Supporting Information), coordinates of **1**-***boat***. An additional argument in favor of this statement is the fact that NMR chemical shifts of C-21 and C-20 are the same in retuline and strychnine. In addition, in retuline (**1**), isoretuline (**2**), and strychnine, the *chair* conformation of ring E is the same (Figure 4).

**Figure 5:**
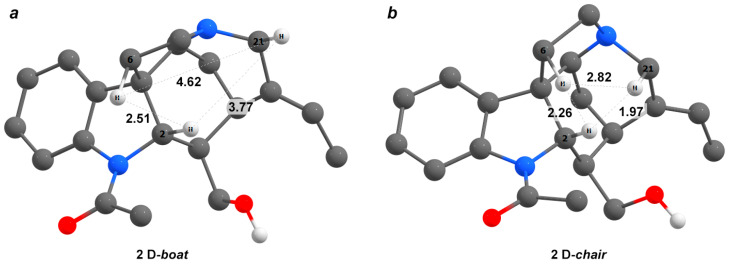
Stereochemical structures of two conformations of the D ring in isoretuline (**2**): *boat* (**a**), and *chair* (**b**).

**Isoretuline** (**2**), like retuline (**1**), is part of the dimeric bisindole alkaloids. However, its main difference is the inverted configuration of the chiral center C-16, which is manifested by the specific values of NMR chemical shifts and spin–spin coupling constants, unlike its stereoisomer retuline. Ring D in the isoretuline structure adopts a *chair* conformation [37], whereas in strychnine, despite a similar configuration of the remaining chiral centers, this cycle adopts a *boat* form, see Figure 4b. The ^13^C NMR data of isoretuline (**2**) were first reported in the paper by Wenkert et al. [39]. Our theoretical modeling of the ^1^H NMR chemical shifts of isoretuline confirmed this hypothesis: for the *boat* conformation of ring D, significant deviations were observed in the case of three protons: H-2, H_β_-6, and H_β_-21 (see Table 1). This is explained by the fact that these conformations have completely different stereochemical structures, which determine the presence or absence of NOE correlations in the *chair* and *boat* forms (see Figure 5).

**Scheme 2:**
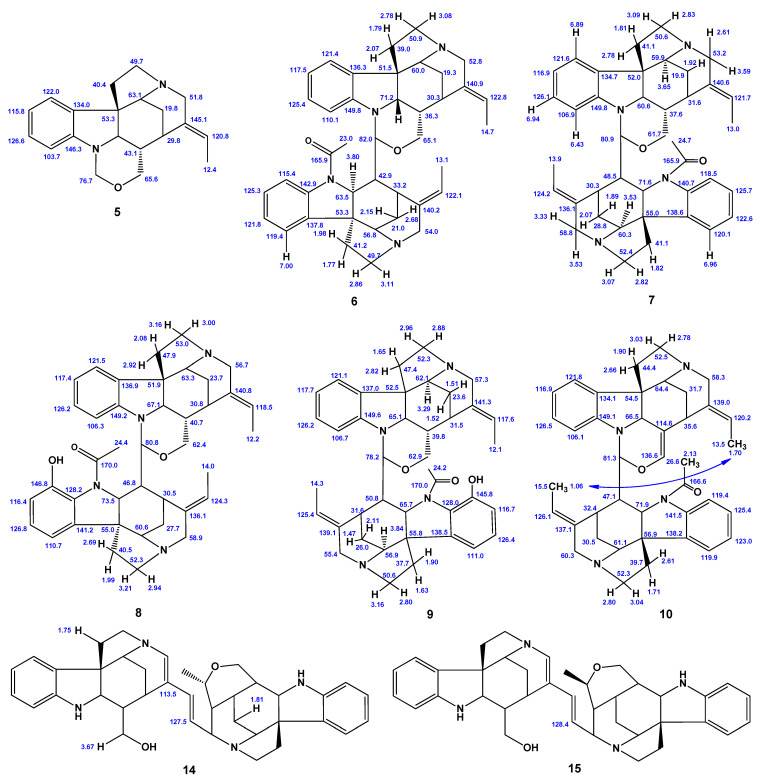
Additional assignments and missing NMR spectral data of **1**–**17**: black—experimental data, blue—computational assignments based on the performed calculations. ND stands for “not determined”.

In general, the main difference between the retuline and isoretuline alkaloids lies in the patterns of their ^13^C NMR chemical shifts of C-2, C-6, C-14, C-16, C-20, and C-21. NMR spectra of this class of alkaloids are often very complex because of several rotamers, which may be present in solution simultaneously. Furthermore, the interpretation of their NMR spectra is complicated by the equilibrium between isomeric monomers [37]. However, theoretical modeling of proton NMR chemical shifts in isoretuline (**2**) showed excellent agreement between calculated and experimental data. Thus, the CMAEs for the *boat* and *chair* conformers of ring D were only 0.18 and 0.09 ppm, respectively. In this study, the unresolved H-9–H-12 multiplet was assigned, and missing experimental data on carbon nuclei were predicted—see Scheme 2.

***N*-deacetylretuline** (**3**) **and *N*-deacetylisoretuline** (**4**). Unlike retuline (**1**) and isoretuline (**2**), removal of the acetyl group in *N*-deacetylretuline (**3**) and *N*-deacetylisoretuline (**4**) is well separated from the nitrogen atom, so that a complex electronic effect observed in the first two compounds is not present in the latter two alkaloids, where theoretical calculations demonstrated excellent accuracy in predicting NMR chemical shifts, including that of the carbon atom at C-8. This fact is confirmed by the high correlation coefficients of calculated chemical shifts as compared to experiment for both the proton ($\bar{R}$ = 0.994) and carbon ($\bar{R}$ = 0.997) NMR data.

**Figure 6:**
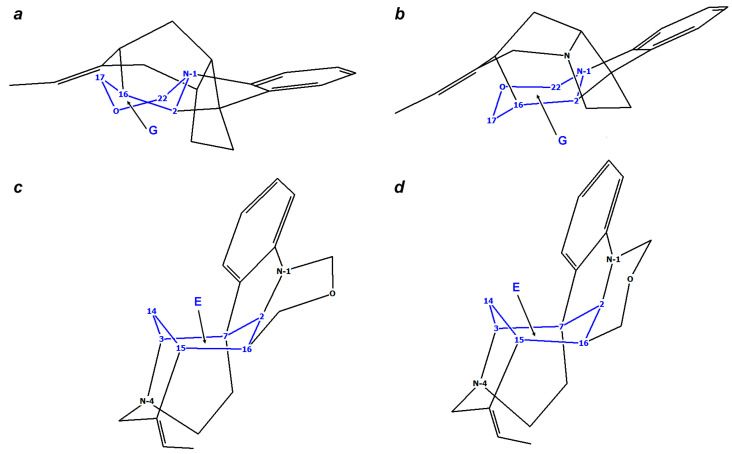
Stereochemical structures of rosibiline **5**: (**a**,**b**)—*twist*-*boat* and *chair* conformations of ring G, respectively; (**c**,**d**)—*boat* conformation of ring E.

**Rosibiline** (**5**). The key difference between this compound (isolated in 1980) and those considered above is the oxazine moiety, which provides a significant effect on the three-dimensional disposition of the rest of the molecule [40]. In this paper, we carried out a detailed study of the conformational behavior of this moiety based on the analysis of ^1^H NMR chemical shifts. In the earlier study [27], it was assumed that the oxazine system G of all strychnobilines had a *boat* conformation, while in rosibiline (**5**) it would adopt a *flat chair*-type conformation according to the coupling pattern of the protons H-16 and the protons H_α_-17 and H_β_-17—see Figure 6b. However, our additional studies of the four most probable conformations of this alkaloid showed that for its ring system, there is still the predominance of the *twist-boat* form shown in Figure 6a. This is also confirmed by the results of the correlation analysis: the CMAE for the *twist-boat* and *chair* conformations of ring G were found to be 0.17 and 0.21 ppm, respectively, while RMSD was in the range of 0.21–0.26 ppm.

It is interesting to note that, based on the data of X-ray analysis, the oxazine ring in the upper strychnane moiety of geisospermine (nevertheless, with an ethylic side chain instead of an ethylidenic one), an alkaloid of the *Corynanthe-Strychnos* type [41], also has an intermediary conformation between *true-boat* and *twist-boat* form, the latter being predominant [42]. This conclusion highlights the complex dynamics of this structural unit and indicates the importance of taking into account multiple conformational states when studying the functional properties and behavior of this alkaloid.

It was also suggested previously [27] that the conformation of the ring E in the four strychnobilines **6**–**9** is a *half-chair*, while, in rosibiline (**5**), it could adopt a *boat* conformation if its oxazinic ring had a *chair* conformation. Our detailed analysis of the conformational behavior of this molecule confirmed that for both forms of the ring G, *twist-boat* and *chair*, the *boat* conformation of the E ring is preferable—see Figure 6c,d. The missing experimental ^13^C NMR data are replenished by our theoretical values, as shown in Scheme 2.

**Strychnobiline** (**6**) and isostrychnobiline (**7**), isolated in 1978 [25], are the first representatives of a group of natural products belonging to the class of asymmetrical dimeric alkaloids with two *Strychnos-Strychnos* subunits linked via a saturated oxazine system (as a part of subunit 1), which are located at the top of the molecules. The lower part (subunit 2) contains the acetamide group, which creates certain difficulties in identifying the true conformer, as discussed below. Moreover, the rotamer with the lowest CMAE has an acyl group oriented toward the ethylidene moiety of the first subunit, rather than toward the aromatic C-8–C-13 system.

Based on the analysis of theoretical and experimental ^1^H NMR chemical shifts, we found that two points, namely those of H-12 and H_β_-17, have an extremely high deviation of about 1.0–1.5 ppm from the mathematical expectation of the entire general sample—see Table 1 and Supporting Information, Figure S1. When examining the three-dimensional structure of strychnobiline (**6**), it is seen that the protons H-12 and H_β_-17 are oriented towards the second subunit (see Supporting Information, coordinates of **6b**). Apparently, this is due to the fact that they are influenced by the non-valent electron interactions, which are difficult to take into account when considering only one conformer (rather than a set of them). Here, we mean a set of rotamers originating from the rotation around the C16′–C17′ bond. These conformers significantly affect the magnitude of NMR chemical shifts, especially in ^1^H NMR spectra.

In order to better understand the reasons for such deviations, we studied in detail six ground states of this dimer that could potentially perturb NMR chemical shifts of H-12 and H_β_-17 protons. These were four isomers of the ethylidene fragments C19–C20 and C19′–C20′, namely *E*-C19–C20/*E*-C19′–C20′, *E*-C19–C20/*Z*-C19′–C20′, *Z*-C19–C20/*E*-C19′–C20′, and *Z*-C19–C20/*Z*-C19′–C20′; however, until now, we have not encountered indolinic alkaloids of *Strychnos*-type with *Z*-ethylidenic chains. The X-ray determinations of this type of alkaloids (e.g., strychnine, *C*-curarine, akuammicine, sungucine, norfluorocurarine, and some others) have always *E*-configuration of the double bond. Moreover, retulines and strychnobilines alkaloids synthesized from strychnine had the same side chains as the natural products. Nevertheless, we had to examine this hypothesis.

In addition, the two most energetically favorable rotamers around the C16′–C17′ bond were also considered. It turned out that in none of these six states could the deviations in the calculated two ^1^H NMR chemical shifts be completely eliminated. Apparently, the reasons for this mysterious fact go far beyond common stereochemical reasons.

Despite this observation, most of the remaining theoretical NMR chemical shifts showed good agreement with the experimental data. It should be noted that no experimental ^13^C NMR data exist for this compound, so the missing information was replenished here with our theoretical values (see Scheme 2).

**Isostrychnobiline** (**7**). In this compound, like in strychnobiline (**6**), the relative orientation of two subunits along the C16′–C17′ bond is of major importance, since the NMR chemical shifts of protons oriented “inward” are strongly dependent on the influence of intramolecular through-space interactions. In particular, salient deviations are observed for protons of the methyl group CH_3_-23′, which are located very close to the opposite subunit 1. To refine actual conformation, we modelled ^13^C NMR chemical shifts for the four most stable rotamers around the C16′–C17′ bond. However, we were unable to completely neutralize this effect: the deviation of the theoretical value from the experiment was found to be 0.50 ppm—see Table 1. Missing information on ^13^C NMR spectral data was supplemented by our calculated values, see Scheme 2.

**12′-hydroxystrychnobiline** (**8**). This alkaloid continues a series of the homologous members of the strychnobiline family. The configurations of strychnobiline (**6**) and its 12′-hydroxy derivative (**8**) differ from the related pair of isostrychnobilins **7** and **9** at two stereocenters, 16′ and 17′, providing absolute configuration 2*S*, 3*S*, 7*R*, 15*R*, 16*S*, 2′*S*, 3′*S*, 7′*R*, 15′*R*, 16′*R*, 17′*R*.

As already mentioned, almost all representatives of this group of alkaloids have a *boat* conformation of the oxazine ring G, see Figure 6a. A distinctive feature of this molecule is the spatial proximity of the functional groups of two subunits, which determines intramolecular non-bonded interactions and causes significant discrepancies in the values of shielding constants, increasing the differences between calculated and experimental NMR data. This is especially pronounced for protons H-12, H-16, H-21, H-15′, and H-16′—see Table 1. Of particular note is the H-12 proton, for which an unaccounted mesomeric effect induced by the N-4 lone pair is observed transmitted through the aromatic system. As in the case of isostrychnobiline (**7**), a detailed conformational study was carried out for the hydroxy derivative **8**, which confirmed the dominant influence of the rotation of two subunits around the C-16′–C-17′ bond.

In the literature, only ^1^H NMR data are available for **6** and **8** published by Tits et al. [26], so that missing ^13^C chemical shifts were complemented here with the corresponding calculated values—see Scheme 2.

**12′-hydroxyisostrychnobiline** (**9**). The structure and stereochemistry of this representative of strychnobilines were first studied in 1979 by Tits and coauthors [27]. 12′-hydroxyisostrychnobiline (**9**) and its deoxy derivative **8** are unsymmetric dimeric alkaloids with two different functional groups linked through the oxazine ring N1–C2–C16–C17–O-C17′, which is present in the upper subunit. The lower part of this natural product has the acetamide function at C-22′–C-23′ and a cryptophenolic function at C-12′. This junction explains the presence of the single rotamer with the acyl oxygen oriented toward the aromatic ring. It is worth noting here that, according to theoretical modeling, such a disposition is not physically feasible for the most stable conformer of **9** (Δ*E*^0^ = 19,010 and 33,818 KJ). However, it is well known that in a natural environment, the predominance of the energetically unfavorable conformers and/or stereoisomers is often possible. Early studies by Tits et al. [27] allowed to propose the absolute configuration of **9** as 2*S*, 3*S*, 7*R*, 15*R*, 16*S*, 2′*S*, 3′*S*, 7′*R*, 15′*S*, 16′*S*, 17′*S* based on the ^1^H NMR data.

**Figure 7:**
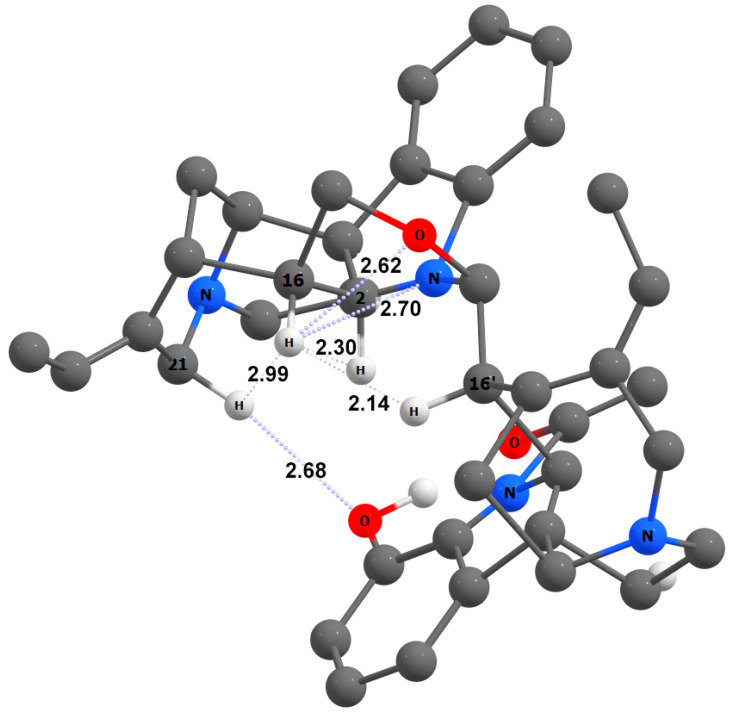
The spatial arrangement of H-16 and H_β_-21 in the most stable rotamer of 12′-hydroxyisostrychnobiline (**9**). Principal intramolecular non-valent interactions are shown as dashed lines. Interatomic distances are given in Å.

As a result of the performed theoretical stereochemical analysis, two marked deviations of C-16 and C-21 chemical shifts were revealed. The reason for these deviations is that the H-16 proton participates in a number of non-valent interactions and forms hydrogen bonds with the nitrogen and oxygen atoms of the upper subunit. As a result, the oxazine system N1–C2–C16–H16–O–C16′ occupies a specific position in space (see Figure 7). Significant stereoelectronic perturbations of the H_β_-21 proton are explained by its participation in the formation of a hydrogen bond with the oxygen atom of the hydroxyl group of the cryptophenolic function; this leads to the overall stabilization of the molecular structure of this particular rotamer.

No experimental ^13^C NMR chemical shifts are available for this compound, so their estimated values based on the performed calculations are provided in Scheme 2.

**Figure 8:**
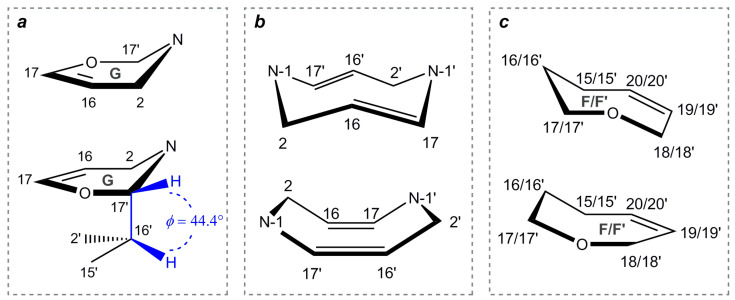
Conformational features of *Strychnos-Strychnos* type bisindole alkaloids: (**a**)—*half-chair* conformation of oxazine ring G of **10**; (**b**)—*crown* and *tub* conformations of cyclooctadiazodien core of **11**; (**c**)—*chair-like* and *boat-like* conformations of oxepin rings F/F’ of **12**/**13**.

**16,17-dehydroisostrychnobiline** (**10**) is actually an isostrychnobiline derivative, which contains a double chemical bond between the C-16 and C-17 atoms, the latter significantly affecting the geometry of the entire molecule. This is the last dimeric alkaloid of the strychnobiline series, which was isolated in 1983 from the roots of *Strychnos variabilis* [28], and later found in the root bark of *Strychnos kasengaensis* [43]. The structure and stereochemistry of **10** were analyzed in more detail in the former paper. According to its ^1^H NMR data, the same configuration as that of isostrychnobiline (**7**) was proposed for this compound, but with a different conformation of the oxazine ring G, namely a *half-chair* instead of a *boat* (see Figure 8a).

**Figure 9:**
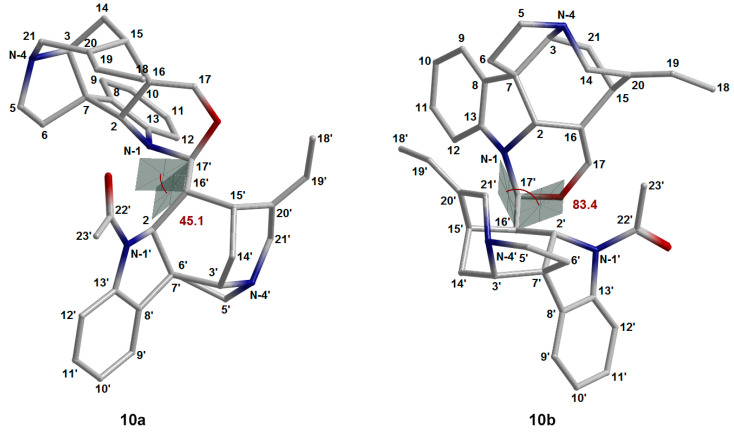
The two most stable conformations of 16,17-dehydroisostrychnobiline (**10**) dealing with the internal rotation around the C-16′–C-17′ bond. Values of the dihedral angles N1–C17′–C16′–C2′ are shown in red.

This hypothesis explained the shielding of the CH_3_-18′ protons and their upfield shift to 1.06 ppm. However, construction of the spatial structures of a number of rotamers of **10** revealed that such an anomalous upfield shift is most likely influenced not by the conformation of the oxazine ring G, but by the rotation around the C-16′–C-17′ bond (see Figure 9). Correlation analysis of several rotamers of **10** clearly demonstrates how a change in the rotation angle affects the change in the NMR chemical shifts. In this case, the methyl group CH_3_-18′ is in the superposition to the aromatic system C8–C13, providing a π-alkyl interaction, which was suggested by Tits et al. [28] (see Supporting Information, coordinates of the spatial structure of **10**).

According to the original study [28], the hydrogen atom H-17′ should occupy the *equatorial* position when the oxazinic ring is in *half-chair* conformation, and this is supported by our theoretical modeling (see Figure 8a). However, the assumption that it is in the *anti*-position to H-16′ contradicts the results of the performed calculations, since such a geometry is impossible for the established and most stable rotamer **10b** (see Figure 9). At that, H-16′ and H-17′ adopt a *syn-clinal* conformation relative to each other as shown in Figure 8a.

The ^1^H NMR chemical shifts of the methyl groups CH_3_-18 and CH_3_-18′, given in the original publication [28], are most probably to be swapped. For the rest of the ^1^H NMR chemical shifts, a comparison of calculated against experimental data demonstrates rather good agreement. It should be noted that no experimental data on ^13^C NMR are available, so here we provide only our calculated values.

**Bisnordihydrotoxiferine** (**11**) is one of the most interesting alkaloids of the *Strychnos-Strychnos* type due to its unique molecular structure. This molecule consists of two indole subunits linked via the B/B’ and G/G’ rings, which form a cyclooctadiazodiene core. The geometry of this central core plays a crucial role in the interpretation of its ^1^H and ^13^C NMR chemical shifts.

It is known that two conformations of cyclooctanes, namely *crown* and *tub*, are most favorable. Therefore, in this study, two corresponding forms of bisnordihydrotoxiferine (**11**), shown in Figure 8b, were analyzed. Although the *crown* form has a higher symmetry group (D_4*d*_) than that of a *tub* (D_2*d*_), the latter conformation was found to be essentially more stable (approximately by 53 kcal/mol).

This can be explained by several factors. First, in the *crown* conformation, strong steric interactions arise between the D/D’ rings, namely, those between H_β_-14–H-15′ and H_β_-14′–H-15 (see Supporting Information, coordinates of **11**-*crown*). These hydrogen atoms are very close, being separated by less than 2 Å, which results in their strong steric repulsion. On the contrary, in the *tub* conformation, these subunits are much more separated in space, which significantly reduces the steric strain of the molecule. Second, in the *crown* conformation, some bonds are eclipsed, leading to the additional torsional strain of the entire bisazacyclooctadiene core. The *tub* form, although experiencing some transannular (across the ring) deformation, is still more stable, since it has no high-energy eclipsed interactions.

In this regard, the highest order of correlation of calculated and experimental ^1^H and ^13^C NMR chemical shifts was achieved for the *tub* conformation: the CMAEs for ^1^H NMR were 0.17 and 0.36 ppm, while CMAEs for ^13^C NMR were 2.66 and 5.17 ppm for the *tub* and *crown* forms, respectively.

**Strychnogucine A** (**12**) is interesting in that its oxepine ring F is capable of adopting two types of conformations: *chair-like* and *boat-like* (see Figure 8c). In order to reliably establish the most probable form of **12**, we performed its theoretical stereochemical analysis. According to the calculation results, it was shown that the smallest deviations were achieved for the C15–C16–C17–O–C18–C19=C20 system, which is in the *chair-like* conformation. At that, the CMAEs for the calculation of ^1^H and ^13^C NMR chemical shifts were as follows: 0.07 and 0.15 ppm for ^1^H NMR, and 1.26 and 1.66 ppm for ^13^C NMR for the *chair-like* and *boat-like* conformations, respectively.

**Strychnogucine B** (**13**). In this compound, as well as in strychnogucine A (**12**), the key element of the stereochemical analysis is the conformation of its oxepine system F’, located in the second indole subunit. Theoretical modeling of the *chair-like* and *boat-like* conformations showed an essential preference for the former, see Figure 8c. Herewith, the difference in CMAE and RMSD values for both ^1^H and ^13^C NMR chemical shifts was found to be more than twice. Moreover, two significant deviations of the calculated NMR data from the experimental ones are observed for carbon atoms in positions C-16 and C-19. In particular, the C-19 atom in the paper by Frédérich et al. [31] was found to be in a highly upfield position, which is not typical for the *sp*^2^-hybridized carbon. The same situation occurs for the *sp*^3^-hybridized C-16, located next to the *sp*^2^-hybridized C-17. Most likely, the source of these discrepancies is typos in the original publication.

**Panganensine R** (**14**). For this first representative of panganensines, an excellent agreement was achieved between calculated and experimental NMR chemical shifts. Unfortunately, experimental data for the C-18 and C-20 atoms are absent from the literature. Moreover, a considerable deviation was found for C-15, which is probably caused by the inductive influence of the neighboring C-20 atom, the latter being a part of the conjugated π-system C18=C19–C20=C21. For ^1^H NMR chemical shifts, experimental data are missing for every second methylene proton at the C-6, C-17, and C-14′ carbons. Since these protons are chemically bound to the same carbon atom, their expected chemical shifts may nevertheless differ significantly. Our theoretical calculations supplemented all missing NMR chemical shifts, which were determined using correlation analysis (see Scheme 2). Also of particular interest is a notable deviation of the calculated chemical shift H-2 from the experiment. This can be explained by the peculiarities of the intramolecular through-space interaction with the hydroxyl group OH-17, i.e., the generation of a five-membered ring resulted in the formation of the H-2–O-17 hydrogen bond.

**Panganensine S** (**15**). In this compound, a marked deviation between calculated and experimental ^13^C NMR chemical shifts was found for the C-19 atom. We explain it as arising due to the complex stereoelectronic effects in the conjugated C18=C19–C20=C21 π-system, which are difficult to take into account when modeling the steady-state equilibrium state of the system. Calculation of the effective geometry, taking into account vibrational corrections, for the structures of bisindole alkaloids is difficult due to their large dimensions. However, in general, calculated ^1^H NMR chemical shifts demonstrated rather good agreement with the experiment. It is interesting to note that the H-2 chemical shift in stereoisomer S fits well into a general correlation trend, which indicates its less effective interaction with the hydroxyl group of OH-17, as compared to that in panganensine R (**14**).

**Panganensine X** (**16**) is an interesting object in regard to its peculiar electronic structure and conformational behavior. Analysis of the ^13^C NMR chemical shifts of all located conformers of **16** revealed three significant deviations from the general correlation pattern dealing with the C-20, C-2′, and C-20′ atoms. The most noticeable discrepancy was observed for the C-20 atom, which was also noted in the previous structures. This is likely due to the effects arising from the delocalization of electron density associated with the interaction of the N-4 lone electron pair and the π-system of the C-20=C-21 double bond. All this causes a disturbance in the electronic structure at this site of the molecule.

**Figure 10:**
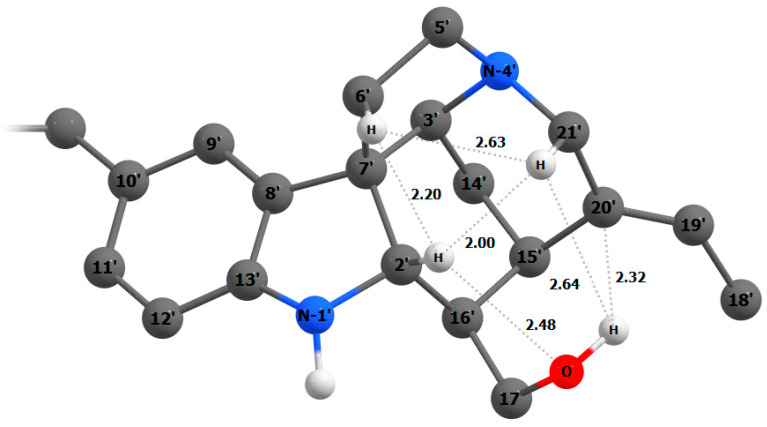
Spatial mapping of the cavity of the bowl-shaped system of rings C’-D’-E’ of the second subunit of panganensine X (**16**). Principal intramolecular non-valent interactions are shown as dashed lines. Interatomic distances are given in Å.

To study the nature of the deviations for the C-2′ and C-20′ atoms in detail, we performed a topological analysis of the electron density distribution within the QTAIM theory. It was found that two bond critical points of the (3,−1) type were localized in the cavity of the bowl-shaped system of the rings C’-D’-E’ of the second subunit (see Supporting Information, Figure S2). Based on this fact, we concluded that the C-2′ atom experienced a non-bonded interaction with the hydroxyl group OH-17′ via the hydrogen atom H-2, which leads to a redistribution of the resulting electron density (Figure 10).

In a similar way, delocalization of electron density occurs through the non-bonded contact H-2′–H-21′, which explains the chemical shift anomalies of the C-2′ atom. The perturbation of the chemical shift of the C-20′ atom is determined primarily by its interaction with the hydroxyl group OH-17′. Furthermore, the deshielding effect of the π-system C-19′=C-20′, which influences observed NMR chemical shifts, must also be taken into account.

Since the experimental chemical shift of the C-6 atom in the ^13^C NMR spectra is unknown, we have estimated it based on the performed calculations. Concerning the ^1^H NMR spectrum, most of the simulated data agree well with the experiment, with the exception of the H-2′ proton. Its deviation arises apparently due to the formation of the intramolecular hydrogen bond with the O-17′ atom. Such effects, caused by delocalization of electron density, are quite difficult to explain at the non-ab initio level, like GIAO-DFT.

**Panganensine Y** (**17**). Unlike previously discussed panganensines, in the stereoisomer Y, the interaction of the OH-17′ hydroxyl group with the H-15 atom becomes more effective, which is reflected in a significant deviation of the calculated NMR chemical shift of C-15 from its experimental value. Moreover, the deviation of the H-15 NMR chemical shift may be caused by the anisotropic influence of the A’ aromatic system. The reason for the upfield shift of the C-21 signal is most probably due to the electron deficiency at the N-4 nitrogen atom, which arises due to the delocalization of the electron density to the C-20=C-21 π-system. For the same reason, deviations of H-14_β_ and H-21 protons do arise; the negative inductive effect here propagates along π-bonds. The resonance signal value of the C-20′ atom is also distorted by partial protonation from the neighboring hydroxyl group, which is located next to the π-system of C-19′=C-20′ double bond.

**Figure 11:**
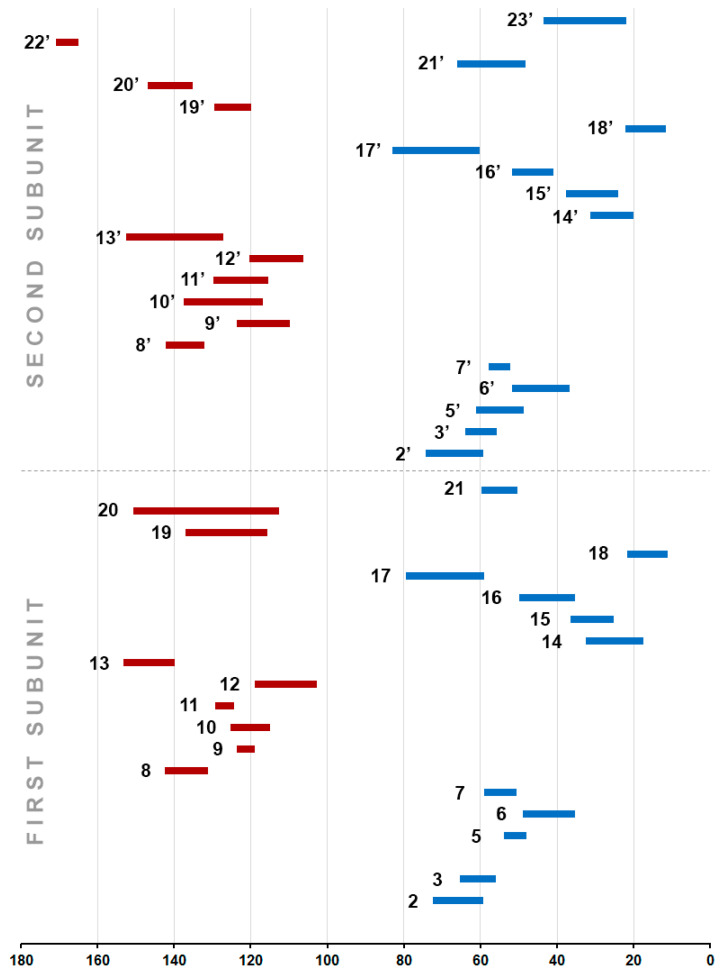
The characteristic ranges of calculated NMR chemical shift values for each of the ^13^C nuclei in the *Strychnos*-type alkaloids **1**–**17**. Blue represents conventionally highfield ranges, and red represents downfield ranges.

Theoretical modeling of ^13^C NMR chemical shift values in *Strychnos*-type alkaloids **1**–**17** allowed us to complete the picture of their distribution ranges on the scale. Figure 11 shows the characteristic ranges of NMR chemical shift values for each of the ^13^C nuclei.

**Figure 12:**
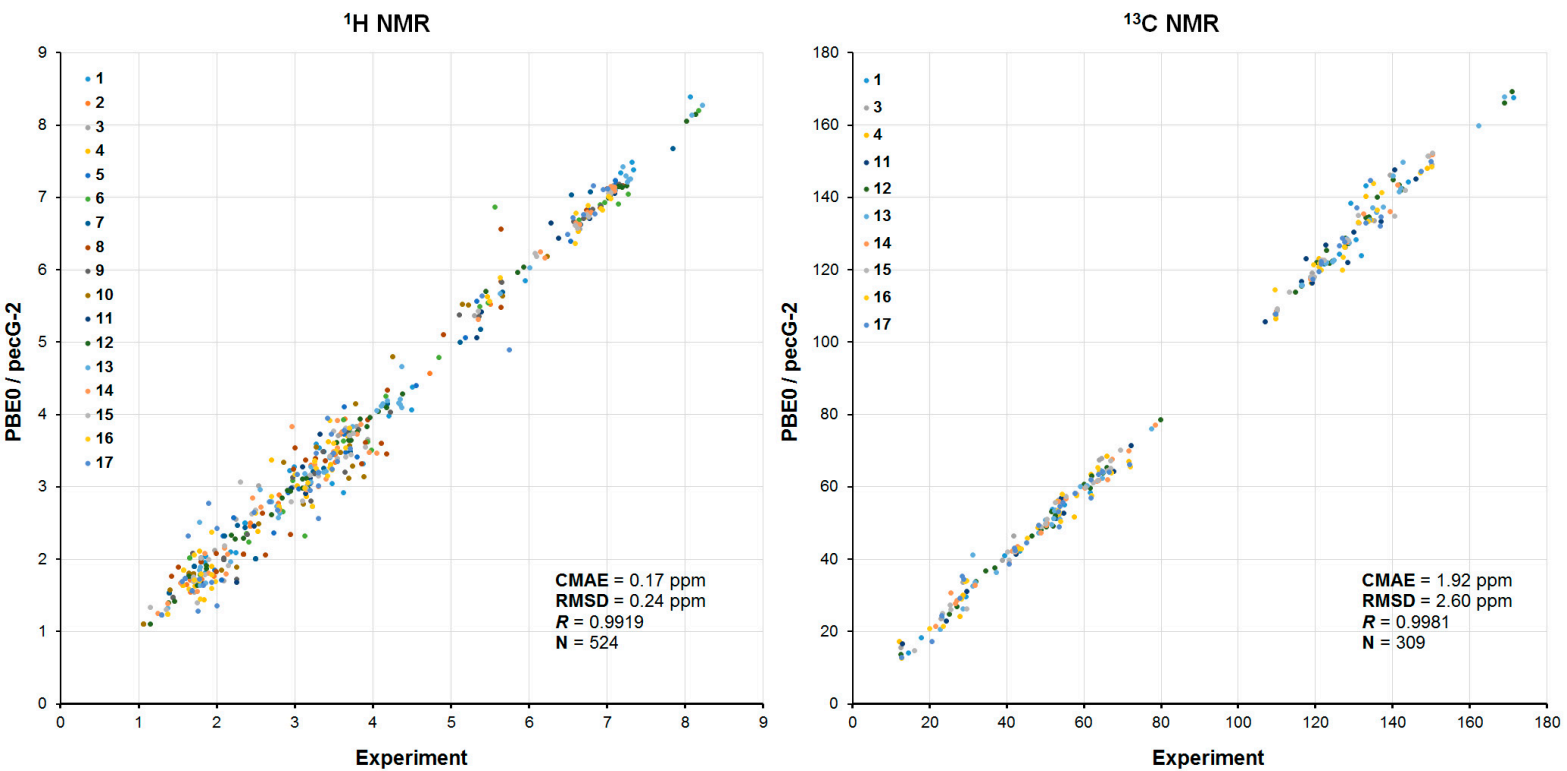
Correlation plots of calculated vs. experimental ^1^H and ^13^C NMR chemical shifts of **1**–**17**, ppm. The points corresponding to each compound are represented by different colors provided in the plots. Given correlation parameters are evaluated for the entire series of **1**–**17**.

As a final illustration, correlation graphs of the calculated and experimental ^1^H and ^13^C NMR chemical shifts of all studied *Strychnos*-type alkaloids are presented in Figure 12. In general, these data illustrate the efficiency of the proposed computational protocol proposed by our team for the stereochemical analysis of natural products. It is evident that the integral CMAE values of NMR chemical shifts (including the reassigned ones) are at the level of 0.2 ppm for protons and 2.0 ppm for carbons, which amounts to ca. 2 and 1% of the studied ranges of accordingly ^1^H and ^13^C NMR chemical shifts. At that, RMSDs are at the level of 0.2 and 2.6 ppm for ^1^H and ^13^C NMR chemical shifts, respectively. All experimental and calculated NMR chemical shifts in the series of the studied alkaloids **1–17** are tabulated in the Supporting Information Excel file.

## 3. Materials and Methods

### 3.1. Optimization of Geometric Parameters

The initial conformational search of alkaloids **1**–**17** was performed using the OPLS3 force field in the liquid phase of a particular solvent, employing the MacroModel module implemented in the Schrödinger Maestro 11.5 package [44]. During this search, approximately 10^5^ steps were performed to identify the most probable conformers/rotamers. Subsequently, the unique conformations of the alkaloids were identified and subjected to further geometry optimization using the Gaussian 09 program [45] at the M06-2X/pecG-2 level [46,47]. The solvation effect was taken into account using the IEF-PCM model developed by Tomasi [48,49].

### 3.2. Modeling (Calculation) of Shielding Constants

Calculations of ^1^H and ^13^C NMR isotropic magnetic shielding constants and corresponding chemical shifts were carried out at the GIAO-DFT level in the liquid phase of a particular solvent using the Gaussian 09 program. In these calculations, we employed the one-parameter hybrid functional PBE0 [50], which was used in combination with Rusakov’s basis set pecS-2 [35,51].

Since verifying the absolute configurations of the studied bisindole dimeric alkaloids **1**–**17**, which contain multiple asymmetric centers, is a highly challenging task, a simple and robust computational framework was employed in this study to elucidate their stereochemical structure. The algorithm for this workflow, presented in the article [36], is based on the application of a set of statistical tools for correlation analysis of calculated isotropic shielding constants and the corresponding NMR chemical shifts of a number of diastereomers and conformers of natural products.

To take into account systematic errors of calculated chemical shifts, we established correlations between their experimental chemical shifts (*x*) and isotropic magnetic shielding constants (*y*), which were further used to find the linear correlation equations of the *y* = *a*x + *b* type. The parameters *a* and *b* were then used for recalculating theoretical chemical shifts using the equation *δ_recalc_* = (*σ_calc_* − *b*)/*a*.

Corrected Mean Absolute Errors (CMAE) were calculated as follows:

(1) $$CMAE=\frac{\sum_{i=1}^{n}\left|\delta_{exp}-\left(\frac{\sigma_{calc}-b}{a}\right)\right|}{n},$$

where *σ_calc_* are the unscaled shielding constants for each of the *n* nuclei in the molecule, while *a* and *b* are the slope and intercept of the linear regression *σ_calc_* = *aδ_exp_* + *b*.

The Root Mean Square Deviation (RMSD) was evaluated as follows:

(2) $$RMSD=\sqrt{\frac{\sum_{i=1}^{n}{\left(\delta_{exp}-\delta_{calc}\right)}^{2}}{n}},$$

where *δ_exp_* and *δ_calc_* are, respectively, the experimental and scaled chemical shifts of each of the *n* nuclei.

### 3.3. Topological Analysis

The wave functions obtained as a result of geometry optimization (M06-2X/pecG-2) were used for further calculations to carry out the topological analysis of the real-space functions. Analysis of the electron density using the obtained wave functions was performed with the AIMAll 19 program [52], which was used to locate and visualize the (3,−1) bond critical points in the space of that descriptor.

## 4. Conclusions

In the present study, we report the results of the high-level computational study of not only 12 bisindole alkaloids of the *Strychnos-Strychnos* type: strychnobiline, isostrychnobiline, 12′-hydroxystrychnobiline, 12′-hydroxyisostrychnobiline, 16,17-dehydroisostrychnobiline, bisnordihydrotoxiferine, strychnogucine A, strychnogucine B, panganensine R, panganensine S, panganensine X, and panganensine Y, but also five monomers (retuline, isoretuline, *N*-deacetylretuline, *N*-deacetylisoretuline, rosibiline) that are frequently implicated in the dimerization path to symmetrical and unsymmetrical bisindolinic alkaloids. Based on the performed theoretical conformational analysis together with a comparison of calculated and experimental NMR chemical shifts, a detailed stereochemical study of these conformationally flexible natural products containing multiple asymmetric centers was performed. Our original methodology is based on simultaneous searching of the multiple conformational states, advanced geometry optimization, and high-level NMR calculations, which were performed synchronously at the DFT level of theory. A number of experimental misassignments published in the literature were corrected, and a number of unknown ^1^H and ^13^C NMR data were supplemented based on the current calculations. In general, a good agreement of the performed calculations with experiment was achieved, which is manifested by the CMAE of about 0.2 ppm for ^1^H and 1.9 ppm for ^13^C NMR chemical shifts in this series of natural products.

## Figures and Tables

**Table 1:** Selected ambiguous values of calculated NMR chemical shifts (ppm) in the series of **1–17**. Experimental values are given in parentheses.

Nucleus	1	4	6	7	8	9	11	13	14	15	16	17
^1^H NMR												
H-2									3.83 (2.96)			
H-3					3.45 (4.18)							
H_β_-6							1.68 (2.26)					
H-12		6.86 (5.56)			6.56 (5.64)							
H_α_-14												2.78 (1.90)
H-15		3.37 (2.70)								3.06 (2.30)		
H-16					2.06 (2.62)	1.73 (2.26)						
H_α_-17											2.73 (3.23)	
H_β_-17		2.32 (3.13)										
H_(α)_-21					3.32 (3.86)							4.90 (5.75)
H_β_-21	2.92 (3.62)					3.20 (3.64)						
H-2′											3.92 (3.45)	
H_β_-14′								2.51 (1.78)				
H-15′					3.54 (3.00)							
H-16′					2.35 (2.94)							
H-23′				2.00 (2.50)								
^13^C NMR												
C-8	138.4 (129.2)											
C-9						122.0 (128.3)						
C-15									30.8 (25.5)			35.3 (28.4)
C-16								41.1 (31.3)				
C-19	123.8 (132.0)							126.6 (64.7)		134.8 (140.6)		
C-20	143.3 (133.0)					147.7 (140.6)					119.9 (127.0)	
C-21												137.1 (130.7)
C-2′											65.6 (72.0)	
C-20′										143.8 (135.1)		144.7 (134.3)

## Data Availability

The original contributions presented in this study are included in the article and Supplementary Materials. Further inquiries can be directed to the corresponding author.

## Supplementary Materials

The following supporting information can be downloaded at https://www.mdpi.com/article/10.3390/ijms27010337/s1, Experimental and calculated NMR chemical shifts (Excel file); Figures S1 and S2; Cartesian coordinates of **1**–**17**.
