# Supplementary material for: Stereochemical Analysis of Natural Products: Bisindole Alkaloids of the Strychnos-Strychnos Type
Source: Int J Mol Sci. 2025 Dec 28;27(1):337. doi: 10.3390/ijms27010337 (PMC12786145; doi:10.3390/ijms27010337)
Supplement: Supplementary file 1 [file ijms-27-00337-s001.zip › Supporting Information R1.pdf]

## SUPPORTING INFORMATION

# Stereochemical analysis of natural products: bisindole alkaloids of *Strychnos-Strychnos* type

**Dmitry A. Grigoriev,** [a] **Valentin A. Semenov,** [a] **Luc Angenot,** [b] **and Leonid B. Krivdin** *[a]

[a] *A.E. Favorsky Irkutsk Institute of Chemistry, Siberian Branch of the Russian Academy of Sciences, 664033 Irkutsk, Russia.*

** Correspondence: semenov@irioch.irk.ru*

[b] *Laboratory of Pharmacognosy, Center of Interdisciplinary Research on Medicines, Faculty of Medicine, University of Liège, Liège, Belgium.*

# TABLE OF CONTENTS

**Figure S1.** Correlation plot of calculated *vs*. experimental $^{1}$H and $^{13}$C NMR chemical shifts of strychnobiline **(6)**, ppm. Points that are out the region of mathematical expectation are shown in red. ........ S4

**Figure S2.** Spatial arrangement of principal non-valent intramolecular interactions and (3,–1) bond critical points in the second subunit of panganensine X (**16**). Bond critical points are shown as green spheres with corresponding numerical values of electron density in a.u. ........ S5

Cartesian coordinates (Angstroms) of main conformers of **1-17**, optimized at the M06-2X/pecG-2 level in the liquid phase of particular solvent, modulated within the IEF-PCM scheme: ........ S6

Compound: **1-*boat*** ........ S6

Compound: **1-*half-chair*** ........ S8

Compound: **2-*chair*** ........ S10

Compound: **2-*boat*** ........ S12

Compound: **3** ........ S14

Compound: **4** ........ S16

Compound: **5-*twist-boat*** ........ S18

Compound: **5-*true-chair*** ........ S20

Compound: **6a** ........ S22

Compound: **6b** ........ S25

Compound: **7a** ........ S28

Compound: **7b** ........ S31

Compound: **7c** ........ S34

Compound: **7d** ........ S37

Compound: **8a** ........ S40

Compound: **8b** ........ S43

Compound: **8c** ........ S46

Compound: **9a** ........ S49

Compound: **9b** ........ S52

Compound: **10a** ........ S55

Compound: **10b** ........ S58

Compound: **11-*tub*** ........ S61

Compound: **11-*crown*** ........ S64

Compound: **12-*boat-like*** ........ S67

Compound: **12-*chair-like*** ........ S70

Compound: **13-*boat-like*** ........ S73
Compound: **13-*chair-like*** ........ S76
Compound: **14a** ........ S79
Compound: **14b** ........ S82
Compound: **14c** ........ S85
Compound: **14d** ........ S88
Compound: **15a** ........ S91
Compound: **15b** ........ S94
Compound: **15c** ........ S97
Compound: **15d** ........ S100
Compound: **16a** ........ S103
Compound: **16b** ........ S106
Compound: **16c** ........ S109
Compound: **16d** ........ S112
Compound: **16e** ........ S115
Compound: **16f** ........ S118
Compound: **17a** ........ S121
Compound: **17b** ........ S124
Compound: **17c** ........ S127
Compound: **17d** ........ S130

**Figure S1.** Correlation plot of calculated *vs*. experimental $^{1}$H and $^{13}$C NMR chemical shifts of strychnobiline **(6)**, ppm. Points that are out the region of mathematical expectation are shown in red.

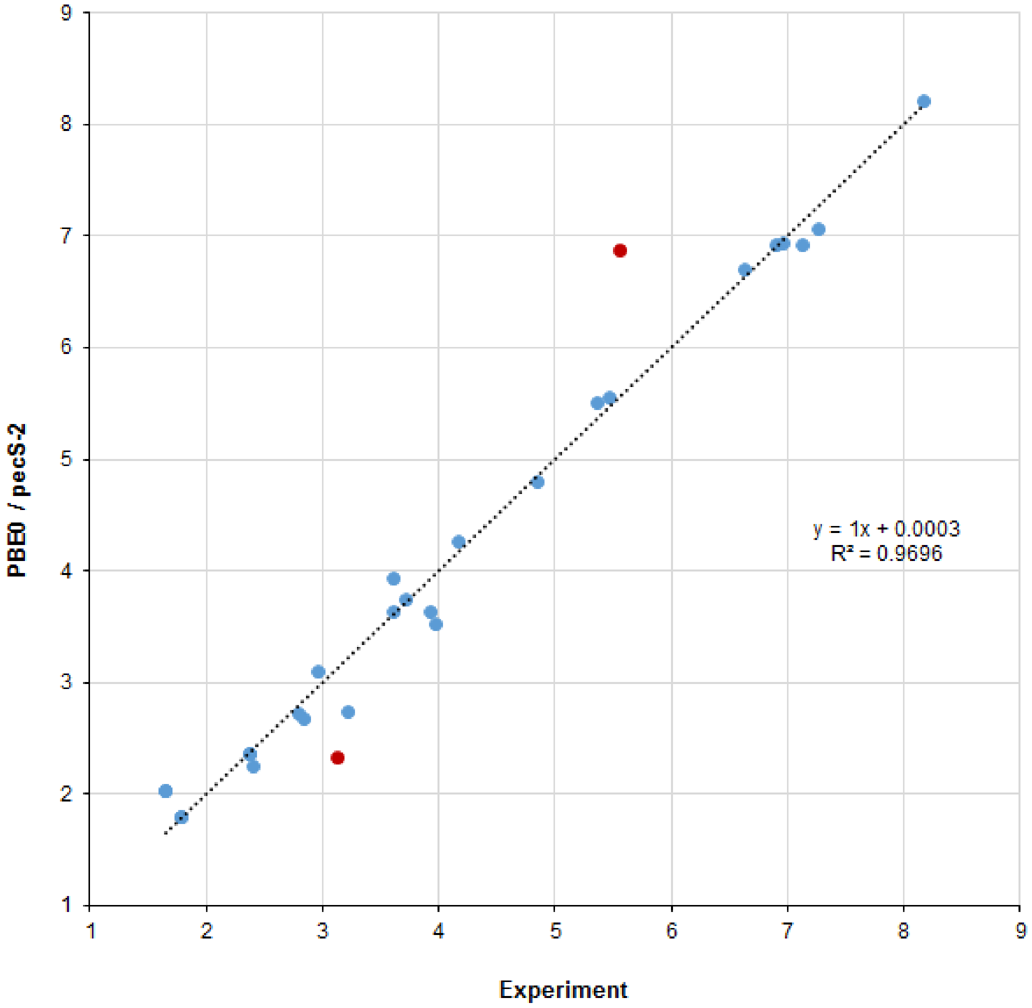

**Figure S2.** Spatial arrangement of principal non-valent intramolecular interactions and (3,–1) bond critical points in the second subunit of panganensine X (**16**). Bond critical points are shown as green spheres with corresponding numerical values of electron density in a.u.

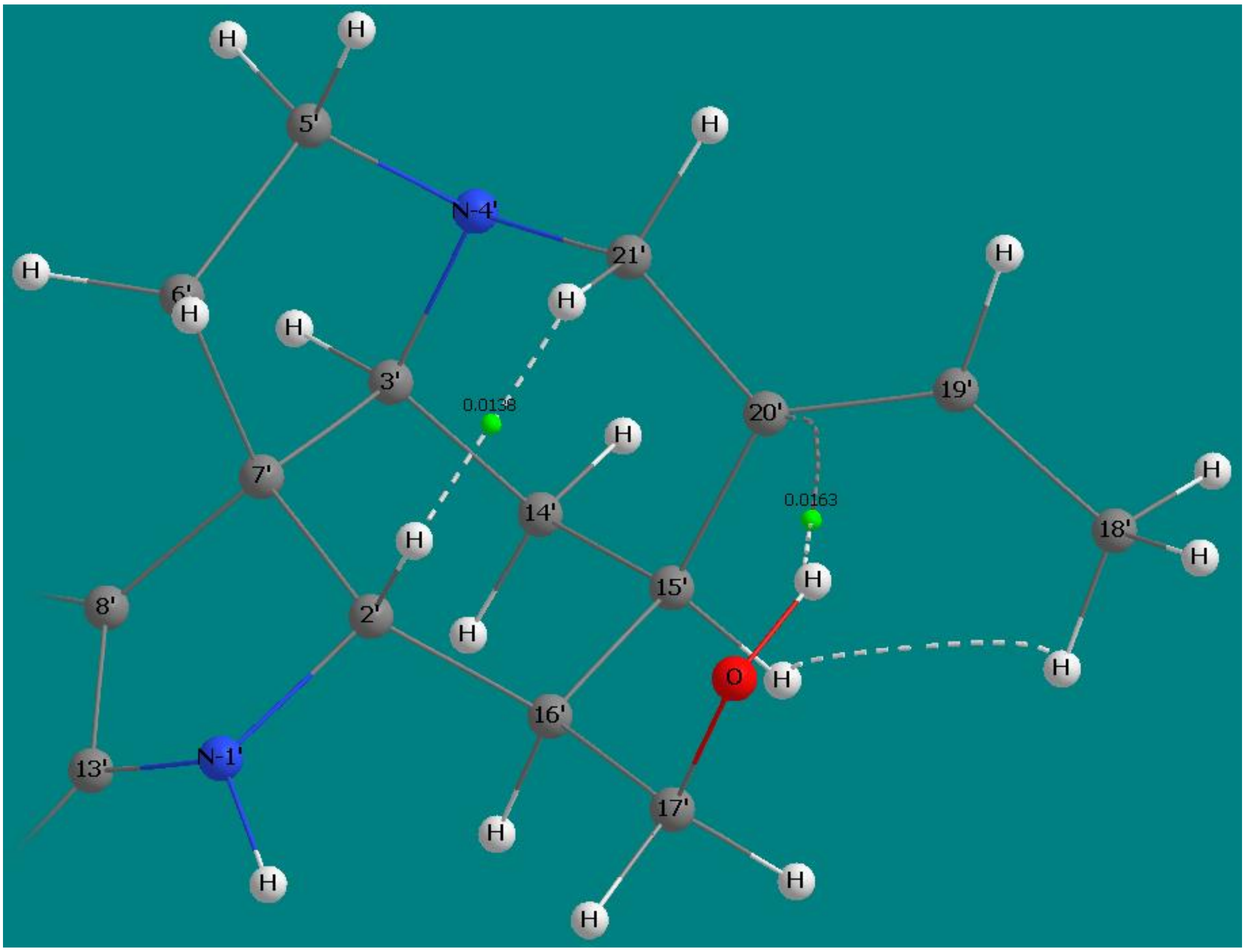

Cartesian coordinates (Angstroms) of main conformers of **1-17**, optimized at the M06-2X/pecG-2 level in the liquid phase of particular solvent, modulated within the IEF-PCM scheme:

Compound: **1-*boat***

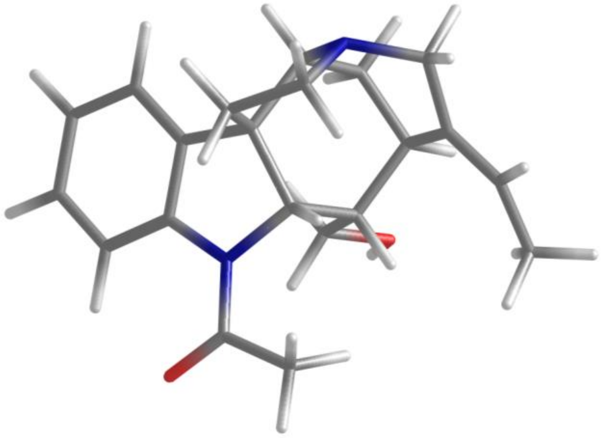

symmetry c1

| | | | |
|---|---|---|---|
| N | -1.227740000 | 1.011492000 | -0.711066000 |
| C | 0.083771000 | 0.367406000 | -0.495553000 |
| C | 0.714856000 | -2.038631000 | 0.263244000 |
| N | 1.788334000 | -2.295945000 | -0.725235000 |
| C | 1.301618000 | -1.924676000 | -2.060965000 |
| C | -0.199231000 | -1.715463000 | -1.890214000 |
| C | -0.291552000 | -1.137884000 | -0.467471000 |
| C | -1.705116000 | -1.093829000 | 0.044949000 |
| C | -2.484864000 | -2.087774000 | 0.598549000 |
| C | -3.806624000 | -1.799691000 | 0.937547000 |
| C | -4.324873000 | -0.534605000 | 0.707144000 |
| C | -3.550595000 | 0.478883000 | 0.143815000 |
| C | -2.236237000 | 0.179177000 | -0.175867000 |
| C | 1.266524000 | -1.436792000 | 1.544115000 |
| C | 1.899185000 | -0.085013000 | 1.216052000 |
| C | 0.795404000 | 0.897040000 | 0.767947000 |
| C | -0.154309000 | 1.244070000 | 1.910741000 |
| C | 3.861023000 | 2.086430000 | 0.253855000 |
| C | 3.782575000 | 0.685648000 | -0.275502000 |
| C | 2.967381000 | -0.277709000 | 0.146667000 |
| C | 3.075102000 | -1.691693000 | -0.380669000 |
| C | -1.435097000 | 2.220604000 | -1.308630000 |
| C | -0.219687000 | 2.896405000 | -1.896838000 |
| H | 0.728355000 | 0.569854000 | -1.346875000 |

| | | | |
|---|---|---|---|
| H | 1.779287000 | -0.999666000 | -2.403947000 |
| H | 1.530828000 | -2.699005000 | -2.792218000 |
| H | -0.731518000 | -2.666164000 | -1.921564000 |
| H | -0.632053000 | -1.055099000 | -2.640225000 |
| H | -2.081475000 | -3.077321000 | 0.765664000 |
| H | -4.428376000 | -2.565291000 | 1.378108000 |
| H | -5.350964000 | -0.321774000 | 0.971110000 |
| H | -3.954161000 | 1.460042000 | -0.034774000 |
| H | 0.209721000 | -2.981517000 | 0.488283000 |
| H | 1.997746000 | -2.112327000 | 1.985580000 |
| H | 0.464825000 | -1.331314000 | 2.272914000 |
| H | 2.356865000 | 0.336044000 | 2.110343000 |
| H | 1.279812000 | 1.835613000 | 0.497198000 |
| H | -0.868409000 | 1.997848000 | 1.572257000 |
| H | -0.727963000 | 0.375970000 | 2.242554000 |
| H | 3.341201000 | 2.198072000 | 1.203190000 |
| H | 4.901454000 | 2.373617000 | 0.401927000 |
| H | 3.435431000 | 2.804318000 | -0.450561000 |
| H | 4.491969000 | 0.436265000 | -1.058503000 |
| H | 3.716414000 | -1.723973000 | -1.259700000 |
| H | 3.559622000 | -2.312208000 | 0.376685000 |
| H | 0.053809000 | 2.044033000 | 3.677872000 |
| H | 0.573373000 | 3.015449000 | -1.162170000 |
| H | -0.523669000 | 3.869784000 | -2.263601000 |
| H | 0.175185000 | 2.307301000 | -2.723925000 |
| O | 0.636327000 | 1.757460000 | 2.972030000 |
| O | -2.543450000 | 2.720189000 | -1.388155000 |

Compound: **1-*half-chair***

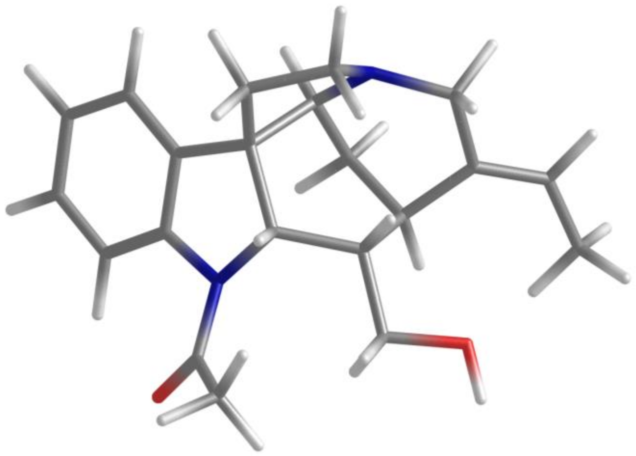

symmetry c1

| | | | |
|---|---|---|---|
| N | 1.383857000 | 1.146436000 | 0.149826000 |
| C | 0.333257000 | 0.403195000 | 0.898043000 |
| C | -0.314157000 | -1.842003000 | -0.205441000 |
| N | -1.400792000 | -2.223000000 | 0.696444000 |
| C | -0.925563000 | -2.187536000 | 2.078172000 |
| C | 0.580144000 | -1.933713000 | 1.975209000 |
| C | 0.702280000 | -1.105459000 | 0.697681000 |
| C | 2.031260000 | -1.056088000 | 0.012231000 |
| C | 2.859493000 | -2.108227000 | -0.327829000 |
| C | 3.994691000 | -1.861198000 | -1.093300000 |
| C | 4.266471000 | -0.571517000 | -1.524108000 |
| C | 3.436996000 | 0.497105000 | -1.194404000 |
| C | 2.325529000 | 0.238459000 | -0.402614000 |
| C | -0.730164000 | -0.956086000 | -1.358085000 |
| C | -1.677074000 | 0.099743000 | -0.793443000 |
| C | -1.133439000 | 0.739771000 | 0.516987000 |
| C | -1.439994000 | 2.229699000 | 0.518226000 |
| C | -4.521019000 | 0.676619000 | -2.006738000 |
| C | -4.133513000 | -0.437636000 | -1.086297000 |
| C | -2.951461000 | -0.654415000 | -0.521834000 |
| C | -2.776301000 | -1.801018000 | 0.466755000 |
| C | 1.756353000 | 2.458420000 | 0.374538000 |
| C | 1.276146000 | 3.188037000 | 1.607878000 |
| H | 0.445090000 | 0.632972000 | 1.958733000 |
| H | -1.412436000 | -1.375790000 | 2.634472000 |
| H | -1.144091000 | -3.113067000 | 2.612753000 |
| H | 1.112949000 | -2.874847000 | 1.839128000 |
| H | 0.986957000 | -1.437843000 | 2.853772000 |
| H | 2.624695000 | -3.114254000 | -0.005372000 |

| | | | |
|---|---|---|---|
| H | 4.653550000 | -2.673743000 | -1.362351000 |
| H | 5.139224000 | -0.382550000 | -2.133028000 |
| H | 3.653265000 | 1.491889000 | -1.538760000 |
| H | 0.191912000 | -2.735472000 | -0.585419000 |
| H | -1.239093000 | -1.519845000 | -2.138592000 |
| H | 0.163212000 | -0.517558000 | -1.804920000 |
| H | -1.843992000 | 0.881512000 | -1.528624000 |
| H | -1.714903000 | 0.331373000 | 1.346090000 |
| H | -1.210611000 | 2.664797000 | 1.490339000 |
| H | -0.843036000 | 2.737826000 | -0.247010000 |
| H | -3.701350000 | 1.361005000 | -2.200434000 |
| H | -4.884137000 | 0.287122000 | -2.958889000 |
| H | -5.336119000 | 1.255904000 | -1.569985000 |
| H | -4.932192000 | -1.132488000 | -0.838704000 |
| H | -3.177898000 | -1.479705000 | 1.433329000 |
| H | -3.401997000 | -2.638751000 | 0.148599000 |
| H | -3.020971000 | 3.320636000 | 0.185500000 |
| H | 0.652969000 | 4.028310000 | 1.312270000 |
| H | 2.173451000 | 3.594798000 | 2.068342000 |
| H | 0.747836000 | 2.583347000 | 2.333016000 |
| O | -2.822598000 | 2.384186000 | 0.243487000 |
| O | 2.532179000 | 3.023746000 | -0.370726000 |

Compound: **2-*chair***

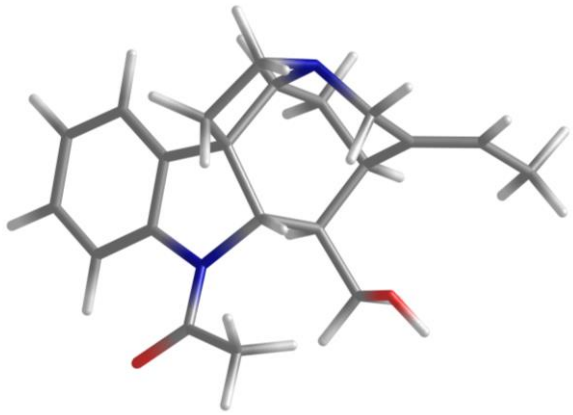

symmetry c1

| | | | |
|---|---|---|---|
| N | 1.457300000 | 1.157812000 | 0.163341000 |
| C | 0.134672000 | 0.511800000 | 0.315775000 |
| C | -0.562996000 | -1.989553000 | 0.071186000 |
| N | -1.556427000 | -2.053621000 | 1.143615000 |
| C | -0.724261000 | -2.136756000 | 2.347757000 |
| C | 0.576221000 | -1.324624000 | 2.064769000 |
| C | 0.502748000 | -0.983398000 | 0.554865000 |
| C | 1.827774000 | -1.076217000 | -0.160195000 |
| C | 2.529356000 | -2.190385000 | -0.577627000 |
| C | 3.765575000 | -2.019441000 | -1.197124000 |
| C | 4.281995000 | -0.745517000 | -1.384793000 |
| C | 3.587814000 | 0.386648000 | -0.964989000 |
| C | 2.358666000 | 0.198026000 | -0.353845000 |
| C | -1.160198000 | -1.672198000 | -1.278430000 |
| C | -1.821020000 | -0.297068000 | -1.195065000 |
| C | -0.711776000 | 0.751976000 | -0.953302000 |
| C | -1.234641000 | 2.176496000 | -1.019473000 |
| C | -4.834039000 | 0.428279000 | -1.597161000 |
| C | -4.197695000 | -0.149973000 | -0.371752000 |
| C | -2.910292000 | -0.403378000 | -0.153891000 |
| C | -2.458003000 | -0.888500000 | 1.199982000 |
| C | 1.855766000 | 2.355786000 | 0.694430000 |
| C | 0.867682000 | 3.092246000 | 1.564067000 |
| H | -0.366669000 | 0.936595000 | 1.177034000 |
| H | -1.274208000 | -1.774698000 | 3.213171000 |
| H | -0.470304000 | -3.180496000 | 2.527580000 |
| H | 1.455468000 | -1.929273000 | 2.276194000 |
| H | 0.651127000 | -0.422140000 | 2.669727000 |
| H | 2.132144000 | -3.184569000 | -0.422183000 |
| H | 4.322788000 | -2.881870000 | -1.532846000 |
| H | 5.240786000 | -0.623256000 | -1.868072000 |
| H | 3.988849000 | 1.375280000 | -1.105257000 |

| | | | |
|---|---|---|---|
| H | -0.082052000 | -2.970957000 | 0.040556000 |
| H | -1.892169000 | -2.433312000 | -1.545392000 |
| H | -0.373721000 | -1.675782000 | -2.034804000 |
| H | -2.264072000 | -0.057838000 | -2.159116000 |
| H | -0.027676000 | 0.644113000 | -1.801087000 |
| H | -0.396555000 | 2.878114000 | -1.084459000 |
| H | -1.833955000 | 2.291853000 | -1.925687000 |
| H | -4.120815000 | 0.659124000 | -2.382950000 |
| H | -5.580536000 | -0.253411000 | -2.006351000 |
| H | -5.358880000 | 1.350587000 | -1.343536000 |
| H | -4.884457000 | -0.350645000 | 0.444797000 |
| H | -1.983546000 | -0.047093000 | 1.723009000 |
| H | -3.325933000 | -1.165851000 | 1.796687000 |
| H | -2.515891000 | 3.254144000 | -0.006826000 |
| H | 1.257442000 | 4.089245000 | 1.735778000 |
| H | 0.791389000 | 2.575517000 | 2.522027000 |
| H | -0.130769000 | 3.141510000 | 1.140932000 |
| O | -2.018143000 | 2.446470000 | 0.133422000 |
| O | 2.980145000 | 2.788565000 | 0.512988000 |

Compound: **2-*boat***

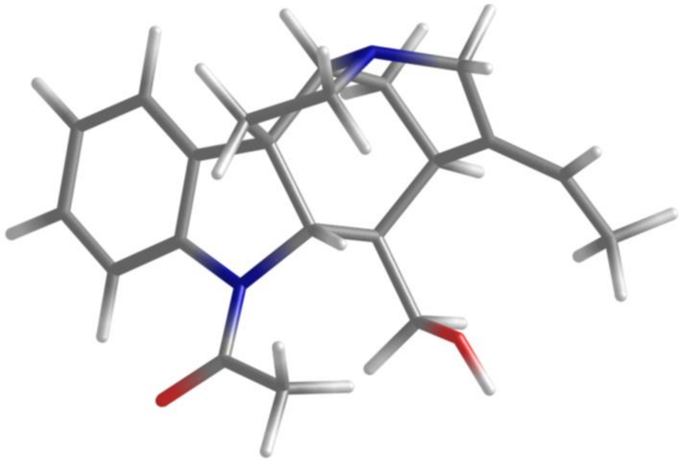

symmetry c1

| | | | |
|---|---|---|---|
| N | 1.344993000 | 1.157927000 | 0.226765000 |
| C | 0.066904000 | 0.412658000 | 0.268723000 |
| C | -0.430260000 | -2.128376000 | 0.034714000 |
| N | -1.435750000 | -2.218668000 | 1.109651000 |
| C | -0.916121000 | -1.559201000 | 2.312495000 |
| C | 0.564896000 | -1.330113000 | 2.018446000 |
| C | 0.544882000 | -1.045442000 | 0.509438000 |
| C | 1.902854000 | -1.032531000 | -0.137731000 |
| C | 2.706268000 | -2.078671000 | -0.545974000 |
| C | 3.960255000 | -1.796454000 | -1.084610000 |
| C | 4.390183000 | -0.483037000 | -1.201859000 |
| C | 3.590717000 | 0.581737000 | -0.791028000 |
| C | 2.346729000 | 0.283562000 | -0.261194000 |
| C | -1.058286000 | -1.847875000 | -1.319331000 |
| C | -1.787132000 | -0.504413000 | -1.255324000 |
| C | -0.697811000 | 0.580784000 | -1.053000000 |
| C | -1.151906000 | 2.012183000 | -1.274244000 |
| C | -4.475579000 | 1.115725000 | -1.179328000 |
| C | -4.029204000 | 0.069262000 | -0.210251000 |
| C | -2.897944000 | -0.630202000 | -0.215308000 |
| C | -2.782184000 | -1.833378000 | 0.714786000 |
| C | 1.633278000 | 2.346056000 | 0.846773000 |
| C | 0.542531000 | 2.975866000 | 1.672678000 |
| H | -0.547420000 | 0.772094000 | 1.090137000 |
| H | -1.424543000 | -0.603849000 | 2.487678000 |
| H | -1.070610000 | -2.175063000 | 3.198385000 |
| H | 1.144836000 | -2.233453000 | 2.208798000 |
| H | 0.999074000 | -0.515057000 | 2.596034000 |
| H | 2.373130000 | -3.102821000 | -0.445553000 |
| H | 4.598883000 | -2.603862000 | -1.412246000 |

| | | | |
|---|---|---|---|
| H | 5.363751000 | -0.275820000 | -1.622997000 |
| H | 3.926082000 | 1.600635000 | -0.877692000 |
| H | 0.126113000 | -3.069217000 | -0.016151000 |
| H | -1.742298000 | -2.653491000 | -1.584777000 |
| H | -0.275984000 | -1.825005000 | -2.078600000 |
| H | -2.244225000 | -0.296784000 | -2.224366000 |
| H | 0.028437000 | 0.398768000 | -1.853022000 |
| H | -0.270080000 | 2.658999000 | -1.328732000 |
| H | -1.665270000 | 2.077058000 | -2.237475000 |
| H | -3.759504000 | 1.274322000 | -1.980656000 |
| H | -5.429081000 | 0.825029000 | -1.623587000 |
| H | -4.633893000 | 2.070043000 | -0.677574000 |
| H | -4.755678000 | -0.179480000 | 0.559089000 |
| H | -3.369638000 | -1.665550000 | 1.616075000 |
| H | -3.244676000 | -2.684097000 | 0.206615000 |
| H | -2.358796000 | 3.299409000 | -0.445369000 |
| H | 0.885962000 | 3.956279000 | 1.982400000 |
| H | 0.362997000 | 2.366226000 | 2.559493000 |
| H | -0.397119000 | 3.051508000 | 1.133284000 |
| O | -2.003702000 | 2.436356000 | -0.223318000 |
| O | 2.738255000 | 2.852335000 | 0.763652000 |

Compound: **3**

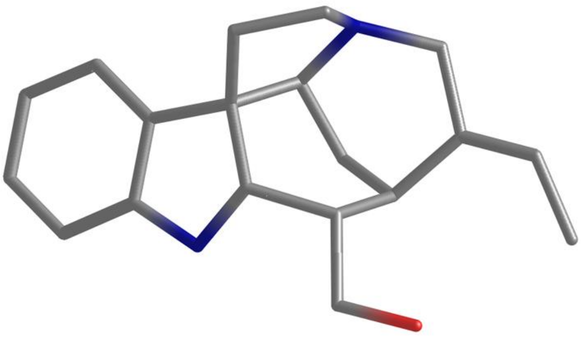

symmetry c1

| | | | |
|---|---|---|---|
| N | 1.228431000 | 1.691614000 | 0.099331000 |
| C | 0.375051000 | 0.740456000 | 0.843438000 |
| C | 0.223731000 | -1.339116000 | -0.668249000 |
| N | -0.725292000 | -2.268347000 | -0.018981000 |
| C | -0.585294000 | -2.196883000 | 1.433183000 |
| C | 0.828363000 | -1.669680000 | 1.627138000 |
| C | 0.979652000 | -0.651828000 | 0.495388000 |
| C | 2.391493000 | -0.283654000 | 0.118417000 |
| C | 3.499437000 | -1.084542000 | -0.074446000 |
| C | 4.705808000 | -0.499429000 | -0.455682000 |
| C | 4.780910000 | 0.874685000 | -0.645689000 |
| C | 3.666862000 | 1.689293000 | -0.469567000 |
| C | 2.473570000 | 1.092254000 | -0.088535000 |
| C | -0.474783000 | -0.317671000 | -1.562489000 |
| C | -1.633253000 | 0.308722000 | -0.778005000 |
| C | -1.120354000 | 0.942020000 | 0.555673000 |
| C | -1.420471000 | 2.431140000 | 0.630782000 |
| C | -4.774311000 | 0.646720000 | -0.642448000 |
| C | -3.984266000 | -0.611517000 | -0.465186000 |
| C | -2.667295000 | -0.781570000 | -0.546667000 |
| C | -2.086737000 | -2.188491000 | -0.513667000 |
| H | 1.250367000 | 2.630555000 | 0.468328000 |
| H | 0.523936000 | 0.901852000 | 1.918123000 |
| H | -1.319039000 | -1.518956000 | 1.887047000 |
| H | -0.732154000 | -3.179308000 | 1.882627000 |
| H | 1.544980000 | -2.478376000 | 1.480792000 |
| H | 1.000187000 | -1.234119000 | 2.609189000 |
| H | 3.437010000 | -2.154810000 | 0.076441000 |
| H | 5.583159000 | -1.112751000 | -0.599545000 |
| H | 5.720995000 | 1.322678000 | -0.936147000 |
| H | 3.730355000 | 2.757383000 | -0.624172000 |
| H | 0.947841000 | -1.909156000 | -1.256523000 |
| H | -0.857782000 | -0.796502000 | -2.463631000 |

| | | | |
|---|---|---|---|
| H | 0.247181000 | 0.434064000 | -1.877284000 |
| H | -2.087456000 | 1.105262000 | -1.366798000 |
| H | -1.665794000 | 0.488920000 | 1.383916000 |
| H | -0.913412000 | 2.863637000 | 1.501552000 |
| H | -1.040707000 | 2.926525000 | -0.267155000 |
| H | -4.161629000 | 1.475794000 | -0.978051000 |
| H | -5.578539000 | 0.481352000 | -1.360418000 |
| H | -5.239967000 | 0.951037000 | 0.296359000 |
| H | -4.577104000 | -1.503915000 | -0.278731000 |
| H | -2.716965000 | -2.844762000 | 0.085982000 |
| H | -2.101597000 | -2.591771000 | -1.529873000 |
| H | -3.024917000 | 3.541687000 | 0.653741000 |
| O | -2.819948000 | 2.609420000 | 0.746432000 |

Compound: **4**

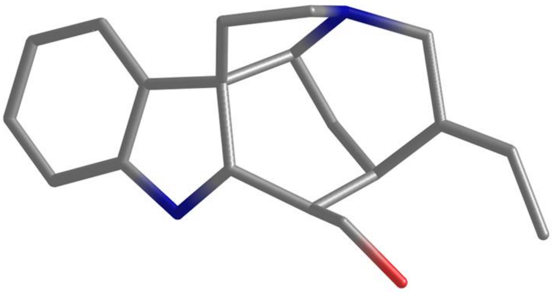

symmetry c1

| | | | |
|---|---|---|---|
| N | -1.368461000 | -1.787342000 | -0.003501000 |
| C | -0.409587000 | -0.904922000 | 0.693007000 |
| C | -0.244575000 | 1.307759000 | -0.634610000 |
| N | 0.730936000 | 2.151687000 | 0.088318000 |
| C | 0.604010000 | 1.934845000 | 1.526864000 |
| C | -0.821253000 | 1.438858000 | 1.683646000 |
| C | -0.996771000 | 0.527951000 | 0.466933000 |
| C | -2.429086000 | 0.233010000 | 0.100593000 |
| C | -3.498894000 | 1.092702000 | -0.050062000 |
| C | -4.744212000 | 0.577330000 | -0.406468000 |
| C | -4.895861000 | -0.788220000 | -0.612343000 |
| C | -3.821137000 | -1.661566000 | -0.479838000 |
| C | -2.587694000 | -1.131963000 | -0.126696000 |
| C | 0.410095000 | 0.363363000 | -1.638386000 |
| C | 1.575586000 | -0.364479000 | -0.960045000 |
| C | 1.028831000 | -1.240179000 | 0.219829000 |
| C | 1.992586000 | -1.426740000 | 1.389868000 |
| C | 4.705673000 | -0.741096000 | -1.046926000 |
| C | 3.943891000 | 0.484415000 | -0.651306000 |
| C | 2.627282000 | 0.676393000 | -0.616667000 |
| C | 2.086267000 | 2.085576000 | -0.420109000 |
| H | -1.394886000 | -2.741440000 | 0.323360000 |
| H | -0.466777000 | -1.099555000 | 1.770003000 |
| H | 1.312055000 | 1.183258000 | 1.896780000 |
| H | 0.797523000 | 2.857568000 | 2.074125000 |
| H | -1.514358000 | 2.277151000 | 1.607883000 |
| H | -1.006850000 | 0.924831000 | 2.624290000 |
| H | -3.377156000 | 2.155838000 | 0.115796000 |
| H | -5.591333000 | 1.238152000 | -0.516599000 |
| H | -5.865446000 | -1.182854000 | -0.882545000 |
| H | -3.943718000 | -2.722103000 | -0.649774000 |
| H | -0.964192000 | 1.946862000 | -1.153001000 |
| H | 0.775479000 | 0.917917000 | -2.502538000 |
| H | -0.338741000 | -0.342760000 | -1.999083000 |

| | | | |
|---|---|---|---|
| H | 2.020949000 | -1.054833000 | -1.672449000 |
| H | 0.920794000 | -2.242320000 | -0.204100000 |
| H | 1.463484000 | -1.915556000 | 2.213458000 |
| H | 2.379748000 | -0.479918000 | 1.756732000 |
| H | 4.058731000 | -1.548684000 | -1.374969000 |
| H | 5.400374000 | -0.495401000 | -1.851938000 |
| H | 5.295158000 | -1.118207000 | -0.211968000 |
| H | 4.563573000 | 1.346663000 | -0.414889000 |
| H | 2.736826000 | 2.657342000 | 0.241500000 |
| H | 2.110083000 | 2.594715000 | -1.387642000 |
| H | 2.850122000 | -3.046758000 | 0.733254000 |
| O | 3.136290000 | -2.179819000 | 1.030861000 |

Compound: **5-*twist-boat***

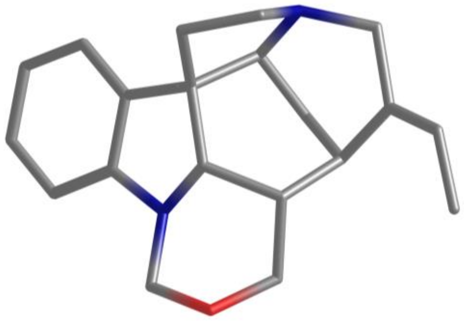

symmetry c1

| | | | |
|---|---|---|---|
| N | 1.242908000 | 1.368505000 | 0.093059000 |
| C | 0.185313000 | 0.574456000 | 0.728004000 |
| C | -0.208887000 | -1.552790000 | -0.698182000 |
| N | -1.285217000 | -2.319212000 | -0.021809000 |
| C | -1.173793000 | -2.121695000 | 1.424107000 |
| C | 0.302308000 | -1.831394000 | 1.620560000 |
| C | 0.608610000 | -0.902313000 | 0.444322000 |
| C | 2.072574000 | -0.748864000 | 0.108050000 |
| C | 3.047133000 | -1.707391000 | -0.063384000 |
| C | 4.347989000 | -1.311056000 | -0.383484000 |
| C | 4.647780000 | 0.035823000 | -0.524405000 |
| C | 3.671062000 | 1.017206000 | -0.363608000 |
| C | 2.380654000 | 0.603882000 | -0.061154000 |
| C | -0.751813000 | -0.506687000 | -1.667108000 |
| C | -1.818783000 | 0.321459000 | -0.949016000 |
| C | -1.180581000 | 1.106150000 | 0.227610000 |
| C | -0.957166000 | 2.565611000 | -0.196319000 |
| C | -4.549807000 | 1.295102000 | 0.029545000 |
| C | -4.127205000 | -0.139103000 | -0.064081000 |
| C | -2.955205000 | -0.585639000 | -0.504157000 |
| C | -2.619413000 | -2.057141000 | -0.545607000 |
| C | 1.282478000 | 2.764602000 | 0.446423000 |
| H | 0.230608000 | 0.730743000 | 1.813557000 |
| H | 0.443674000 | -2.245341000 | -1.234860000 |
| H | -1.775500000 | -1.268680000 | 1.770583000 |
| H | -1.515315000 | -3.007009000 | 1.959625000 |
| H | 0.877707000 | -2.751937000 | 1.515834000 |
| H | 0.538988000 | -1.382499000 | 2.582489000 |
| H | 2.812076000 | -2.757494000 | 0.056781000 |
| H | 5.122029000 | -2.053098000 | -0.512390000 |
| H | 5.659348000 | 0.335025000 | -0.761103000 |
| H | 3.914903000 | 2.064087000 | -0.474975000 |
| H | -1.174709000 | -0.983474000 | -2.550355000 |
| H | 0.070499000 | 0.124975000 | -2.006692000 |

| | | | |
|---|---|---|---|
| H | -2.224733000 | 1.056428000 | -1.647802000 |
| H | -1.871201000 | 1.100579000 | 1.070327000 |
| H | -0.593874000 | 2.604241000 | -1.228597000 |
| H | -1.885325000 | 3.129687000 | -0.137388000 |
| H | -3.880417000 | 1.958296000 | -0.513805000 |
| H | -5.554445000 | 1.429597000 | -0.370178000 |
| H | -4.575847000 | 1.627761000 | 1.069297000 |
| H | -4.854564000 | -0.871726000 | 0.272010000 |
| H | -3.344383000 | -2.631593000 | 0.030317000 |
| H | -2.665686000 | -2.427981000 | -1.571913000 |
| H | 1.832021000 | 2.944599000 | 1.377901000 |
| H | 1.756595000 | 3.324456000 | -0.367575000 |
| O | -0.033221000 | 3.210500000 | 0.663934000 |

Compound: **5-*true-chair***

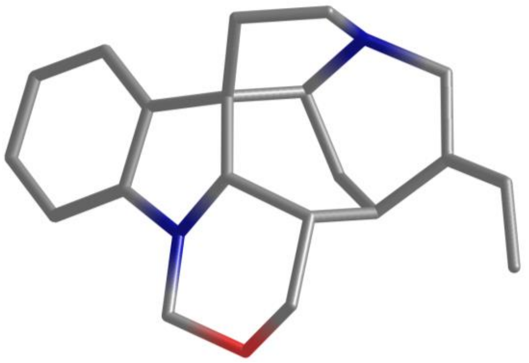

symmetry c1

| | | | |
|---|---|---|---|
| N | 1.163440000 | 1.358756000 | 0.142459000 |
| C | 0.235006000 | 0.495196000 | 0.871716000 |
| C | -0.087347000 | -1.467125000 | -0.753100000 |
| N | -1.153947000 | -2.327052000 | -0.189014000 |
| C | -1.057480000 | -2.338968000 | 1.270302000 |
| C | 0.398953000 | -1.987105000 | 1.536441000 |
| C | 0.698791000 | -0.932018000 | 0.473645000 |
| C | 2.155238000 | -0.681264000 | 0.154345000 |
| C | 3.195396000 | -1.566384000 | -0.019739000 |
| C | 4.456874000 | -1.080743000 | -0.374482000 |
| C | 4.648049000 | 0.280890000 | -0.554174000 |
| C | 3.604813000 | 1.190748000 | -0.388827000 |
| C | 2.357473000 | 0.692636000 | -0.039216000 |
| C | -0.655963000 | -0.334366000 | -1.602747000 |
| C | -1.742939000 | 0.385115000 | -0.797823000 |
| C | -1.205251000 | 0.915880000 | 0.566238000 |
| C | -1.275376000 | 2.440503000 | 0.698262000 |
| C | -4.631199000 | 1.165198000 | -0.080897000 |
| C | -4.121424000 | -0.236533000 | -0.231415000 |
| C | -2.885308000 | -0.592956000 | -0.566100000 |
| C | -2.486439000 | -2.048935000 | -0.706189000 |
| C | 0.986124000 | 2.769508000 | 0.282981000 |
| H | 0.405848000 | 0.623619000 | 1.952900000 |
| H | 0.591609000 | -2.076546000 | -1.354166000 |
| H | -1.723224000 | -1.599200000 | 1.733822000 |
| H | -1.333787000 | -3.316433000 | 1.665304000 |
| H | 1.026084000 | -2.862189000 | 1.363827000 |
| H | 0.581996000 | -1.628946000 | 2.547325000 |
| H | 3.040760000 | -2.628998000 | 0.117662000 |
| H | 5.282634000 | -1.764441000 | -0.504049000 |
| H | 5.627825000 | 0.650968000 | -0.821991000 |
| H | 3.772817000 | 2.249351000 | -0.524048000 |
| H | -1.086605000 | -0.731202000 | -2.521576000 |

| | | | |
|---|---|---|---|
| H | 0.143377000 | 0.344747000 | -1.889471000 |
| H | -2.104241000 | 1.237926000 | -1.373330000 |
| H | -1.841776000 | 0.526034000 | 1.360678000 |
| H | -2.250987000 | 2.810274000 | 0.393118000 |
| H | -1.113616000 | 2.719216000 | 1.747204000 |
| H | -4.040241000 | 1.878269000 | -0.652102000 |
| H | -5.664144000 | 1.239825000 | -0.416069000 |
| H | -4.612272000 | 1.482242000 | 0.964460000 |
| H | -4.838220000 | -1.028055000 | -0.036130000 |
| H | -3.203626000 | -2.689366000 | -0.193930000 |
| H | -2.510393000 | -2.335511000 | -1.759943000 |
| H | 1.142382000 | 3.094342000 | 1.324868000 |
| H | 1.677533000 | 3.296863000 | -0.368770000 |
| O | -0.320517000 | 3.108703000 | -0.108127000 |

Compound: **6a**

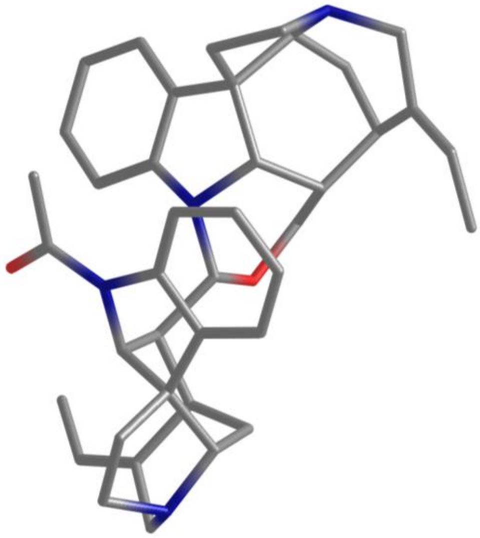

symmetry c1

| | | | |
|---|---|---|---|
| N | 0.583381000 | 0.767489000 | 0.012289000 |
| C | 1.826811000 | -0.045311000 | -0.127485000 |
| C | 4.327173000 | 0.701488000 | -0.239351000 |
| N | 4.959404000 | -0.441808000 | 0.459057000 |
| C | 4.175191000 | -0.747575000 | 1.662108000 |
| C | 3.295208000 | 0.478103000 | 1.867389000 |
| C | 2.947427000 | 0.876128000 | 0.423616000 |
| C | 2.330810000 | 2.240823000 | 0.296969000 |
| C | 2.927169000 | 3.478389000 | 0.431323000 |
| C | 2.153615000 | 4.628846000 | 0.293468000 |
| C | 0.797260000 | 4.511489000 | 0.032876000 |
| C | 0.181826000 | 3.268271000 | -0.089593000 |
| C | 0.958841000 | 2.123483000 | 0.045521000 |
| C | 4.262566000 | 0.487505000 | -1.741394000 |
| C | 3.424421000 | -0.758984000 | -2.022309000 |
| C | 1.969304000 | -0.509334000 | -1.598579000 |
| C | 1.255110000 | 0.476670000 | -2.518261000 |
| C | 2.525027000 | -3.692206000 | -2.339607000 |
| C | 3.600167000 | -3.202583000 | -1.416575000 |
| C | 4.028715000 | -1.949891000 | -1.285489000 |
| C | 5.198954000 | -1.603847000 | -0.393226000 |
| N | -1.633343000 | 0.197118000 | 1.833213000 |
| C | -2.534462000 | 0.094639000 | 0.673713000 |
| C | -3.423696000 | -1.921760000 | -0.689149000 |
| N | -4.863791000 | -1.560327000 | -0.680136000 |
| C | -5.263905000 | -1.235141000 | 0.696691000 |
| C | -4.093072000 | -1.675348000 | 1.565231000 |
| C | -2.879810000 | -1.409652000 | 0.654789000 |
| C | -1.628851000 | -2.009305000 | 1.237771000 |

| | | | |
|---|---|---|---|
| C | -1.141780000 | -3.296611000 | 1.162152000 |
| C | 0.012197000 | -3.632234000 | 1.870599000 |
| C | 0.633186000 | -2.680242000 | 2.661651000 |
| C | 0.149779000 | -1.374601000 | 2.746878000 |
| C | -0.973789000 | -1.036395000 | 2.008233000 |
| C | -2.728849000 | -1.341487000 | -1.910524000 |
| C | -2.779001000 | 0.185644000 | -1.820105000 |
| C | -1.908478000 | 0.650786000 | -0.624790000 |
| C | -0.445047000 | 0.245670000 | -0.867084000 |
| C | -3.742072000 | 3.104256000 | -1.771803000 |
| C | -4.623882000 | 1.897105000 | -1.647334000 |
| C | -4.232006000 | 0.626646000 | -1.688088000 |
| C | -5.222696000 | -0.514551000 | -1.641426000 |
| C | -1.424851000 | 1.432961000 | 2.379194000 |
| C | -0.397525000 | 1.585883000 | 3.462965000 |
| H | 1.727027000 | -0.931539000 | 0.501117000 |
| H | 4.904767000 | 1.604870000 | -0.029363000 |
| H | 4.825258000 | -0.945624000 | 2.513818000 |
| H | 3.550230000 | -1.636587000 | 1.507215000 |
| H | 3.856024000 | 1.286641000 | 2.337698000 |
| H | 2.414615000 | 0.269191000 | 2.469233000 |
| H | 3.986457000 | 3.556516000 | 0.638493000 |
| H | 2.607397000 | 5.604248000 | 0.388734000 |
| H | 0.193496000 | 5.401575000 | -0.074780000 |
| H | -0.879188000 | 3.224404000 | -0.263145000 |
| H | 5.267496000 | 0.379912000 | -2.147108000 |
| H | 3.829561000 | 1.370224000 | -2.209998000 |
| H | 3.423039000 | -0.968933000 | -3.094809000 |
| H | 1.437417000 | -1.456094000 | -1.691242000 |
| H | 1.685582000 | 1.476935000 | -2.435293000 |
| H | 1.339569000 | 0.155623000 | -3.556287000 |
| H | 2.842411000 | -4.609346000 | -2.834272000 |
| H | 1.609397000 | -3.928261000 | -1.792985000 |
| H | 2.277233000 | -2.963352000 | -3.108746000 |
| H | 4.089929000 | -3.964968000 | -0.818873000 |
| H | 5.457180000 | -2.450116000 | 0.241304000 |
| H | 6.074586000 | -1.405253000 | -1.015510000 |
| H | -3.416062000 | 0.695542000 | 0.875748000 |
| H | -3.335005000 | -3.011341000 | -0.693936000 |
| H | -6.191028000 | -1.740369000 | 0.965508000 |
| H | -5.432802000 | -0.158582000 | 0.805345000 |
| H | -4.144578000 | -2.743240000 | 1.778797000 |
| H | -4.031437000 | -1.138004000 | 2.510042000 |
| H | -1.650640000 | -4.039523000 | 0.562720000 |

| | | | |
|---|---|---|---|
| H | 0.412120000 | -4.633790000 | 1.812593000 |
| H | 1.518446000 | -2.942783000 | 3.223589000 |
| H | 0.667487000 | -0.666510000 | 3.368057000 |
| H | -1.704391000 | -1.703858000 | -1.969940000 |
| H | -3.226099000 | -1.692611000 | -2.813354000 |
| H | -2.358466000 | 0.622804000 | -2.722599000 |
| H | -1.965850000 | 1.729704000 | -0.559388000 |
| H | -0.373956000 | -0.834466000 | -0.726474000 |
| H | -2.795741000 | 2.870187000 | -2.257480000 |
| H | -4.238526000 | 3.878274000 | -2.354753000 |
| H | -3.520076000 | 3.535369000 | -0.792435000 |
| H | -5.685005000 | 2.093055000 | -1.530702000 |
| H | -5.299416000 | -0.961925000 | -2.634666000 |
| H | -6.216875000 | -0.148324000 | -1.392078000 |
| H | 0.596296000 | 1.482712000 | 3.025179000 |
| H | -0.498049000 | 2.583151000 | 3.875758000 |
| H | -0.517797000 | 0.843325000 | 4.247847000 |
| O | -0.137996000 | 0.551505000 | -2.222696000 |
| O | -2.068692000 | 2.389429000 | 1.979528000 |

Compound: **6b**

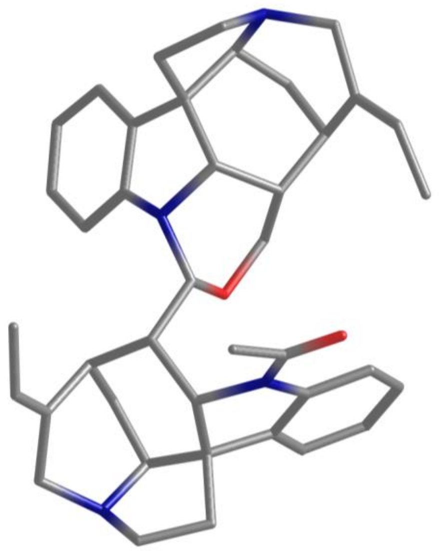

symmetry c1

| | | | |
|---|---|---|---|
| N | -0.938613000 | -1.222294000 | 0.389905000 |
| C | -2.159991000 | -0.371284000 | 0.533583000 |
| C | -4.578170000 | -0.726536000 | -0.365719000 |
| N | -5.335223000 | -0.108816000 | 0.743072000 |
| C | -4.785311000 | -0.596688000 | 2.015136000 |
| C | -3.927232000 | -1.800162000 | 1.632637000 |
| C | -3.333745000 | -1.352296000 | 0.289242000 |
| C | -2.675197000 | -2.421760000 | -0.533860000 |
| C | -3.261971000 | -3.425057000 | -1.277730000 |
| C | -2.462218000 | -4.253759000 | -2.062052000 |
| C | -1.095801000 | -4.032794000 | -2.107430000 |
| C | -0.494076000 | -3.030695000 | -1.348370000 |
| C | -1.286522000 | -2.249809000 | -0.516770000 |
| C | -4.244228000 | 0.262937000 | -1.468530000 |
| C | -3.399341000 | 1.392157000 | -0.882516000 |
| C | -2.036524000 | 0.852057000 | -0.420420000 |
| C | -1.098346000 | 0.548776000 | -1.594153000 |
| C | -2.549128000 | 3.974002000 | 0.547575000 |
| C | -3.745190000 | 3.140373000 | 0.897707000 |
| C | -4.153823000 | 2.037253000 | 0.275848000 |
| C | -5.444907000 | 1.345109000 | 0.653914000 |
| N | 1.883221000 | 1.787273000 | 0.913176000 |
| C | 2.556423000 | 0.480995000 | 0.772852000 |
| C | 3.860946000 | -0.577665000 | -1.210938000 |
| N | 5.040229000 | -1.088989000 | -0.468802000 |
| C | 5.538730000 | -0.032668000 | 0.422095000 |
| C | 4.827962000 | 1.233152000 | -0.034967000 |
| C | 3.449350000 | 0.707585000 | -0.476338000 |

| | | | |
|---|---|---|---|
| C | 2.683861000 | 1.760244000 | -1.230012000 |
| C | 2.770779000 | 2.153869000 | -2.546708000 |
| C | 1.973174000 | 3.209652000 | -2.991484000 |
| C | 1.122722000 | 3.857912000 | -2.110573000 |
| C | 1.031035000 | 3.474920000 | -0.771555000 |
| C | 1.817530000 | 2.414917000 | -0.353761000 |
| C | 2.755029000 | -1.617675000 | -1.300474000 |
| C | 2.284385000 | -1.949858000 | 0.117941000 |
| C | 1.569689000 | -0.727962000 | 0.715376000 |
| C | 0.190248000 | -0.326797000 | 0.174485000 |
| C | 2.131209000 | -3.046145000 | 3.000799000 |
| C | 3.388499000 | -2.835718000 | 2.210340000 |
| C | 3.479332000 | -2.391650000 | 0.959266000 |
| C | 4.799451000 | -2.352563000 | 0.225242000 |
| C | 1.348503000 | 2.285650000 | 2.065372000 |
| C | 1.583847000 | 1.485713000 | 3.324151000 |
| H | -2.197447000 | 0.005282000 | 1.555611000 |
| H | -5.169667000 | -1.543768000 | -0.785615000 |
| H | -5.581491000 | -0.856651000 | 2.711862000 |
| H | -4.167061000 | 0.172710000 | 2.493373000 |
| H | -4.547873000 | -2.683039000 | 1.475963000 |
| H | -3.165819000 | -2.038096000 | 2.373012000 |
| H | -4.335862000 | -3.556895000 | -1.268454000 |
| H | -2.907520000 | -5.040723000 | -2.652581000 |
| H | -0.474996000 | -4.639447000 | -2.751667000 |
| H | 0.561723000 | -2.862400000 | -1.454681000 |
| H | -5.159220000 | 0.655120000 | -1.910570000 |
| H | -3.711026000 | -0.259891000 | -2.262210000 |
| H | -3.211942000 | 2.148134000 | -1.649142000 |
| H | -1.562013000 | 1.654649000 | 0.144921000 |
| H | -1.514042000 | -0.222875000 | -2.244799000 |
| H | -0.944601000 | 1.454144000 | -2.182390000 |
| H | -2.806105000 | 5.032342000 | 0.579136000 |
| H | -1.731138000 | 3.823598000 | 1.256344000 |
| H | -2.168726000 | 3.749244000 | -0.448141000 |
| H | -4.343852000 | 3.499306000 | 1.729067000 |
| H | -5.813543000 | 1.723711000 | 1.605950000 |
| H | -6.206764000 | 1.588624000 | -0.090757000 |
| H | 3.191736000 | 0.313329000 | 1.638950000 |
| H | 4.175383000 | -0.296087000 | -2.218598000 |
| H | 6.622905000 | 0.053843000 | 0.359994000 |
| H | 5.287677000 | -0.255685000 | 1.465797000 |
| H | 5.328187000 | 1.669156000 | -0.899729000 |
| H | 4.756937000 | 1.995805000 | 0.739104000 |

| | | | |
|---|---|---|---|
| H | 3.442792000 | 1.652548000 | -3.230137000 |
| H | 2.023151000 | 3.523961000 | -4.023837000 |
| H | 0.512377000 | 4.678195000 | -2.461628000 |
| H | 0.371838000 | 3.981102000 | -0.088287000 |
| H | 1.938128000 | -1.209616000 | -1.891126000 |
| H | 3.123502000 | -2.506571000 | -1.812076000 |
| H | 1.573813000 | -2.773606000 | 0.119252000 |
| H | 1.339951000 | -0.974168000 | 1.750999000 |
| H | -0.057682000 | 0.517680000 | 0.818538000 |
| H | 1.243903000 | -3.068315000 | 2.370480000 |
| H | 2.183226000 | -3.990474000 | 3.540861000 |
| H | 1.992633000 | -2.263775000 | 3.750293000 |
| H | 4.311046000 | -3.112979000 | 2.710686000 |
| H | 4.822227000 | -3.164632000 | -0.504707000 |
| H | 5.624881000 | -2.532459000 | 0.911753000 |
| H | 2.646149000 | 1.326444000 | 3.499466000 |
| H | 1.157945000 | 2.038838000 | 4.153082000 |
| H | 1.103146000 | 0.510615000 | 3.263322000 |
| O | 0.200472000 | 0.114421000 | -1.160977000 |
| O | 0.713271000 | 3.326475000 | 2.085556000 |

Compound: **7a**

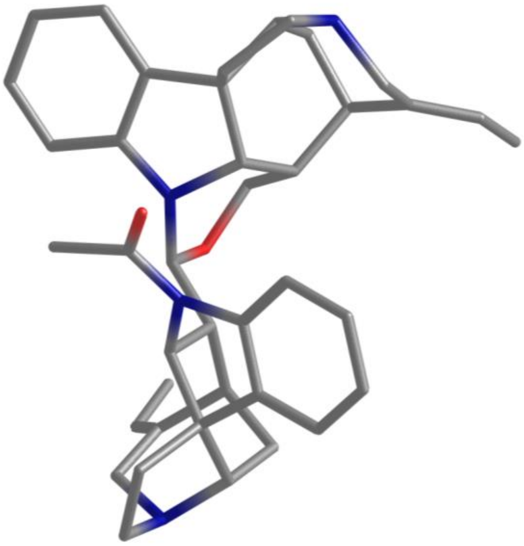

symmetry c1

| | | | |
|---|---|---|---|
| N | 0.718196000 | -1.147380000 | -0.448133000 |
| C | 1.572529000 | -0.037159000 | 0.033329000 |
| C | 4.182378000 | 0.173217000 | -0.100845000 |
| N | 4.345705000 | 1.625049000 | -0.182881000 |
| C | 4.037162000 | 1.888615000 | -1.590690000 |
| C | 2.894391000 | 0.911642000 | -1.985694000 |
| C | 2.862308000 | -0.148116000 | -0.849269000 |
| C | 2.784276000 | -1.569950000 | -1.346006000 |
| C | 3.727261000 | -2.323715000 | -2.014439000 |
| C | 3.422821000 | -3.635465000 | -2.379311000 |
| C | 2.181088000 | -4.168721000 | -2.067121000 |
| C | 1.216776000 | -3.415212000 | -1.401401000 |
| C | 1.530123000 | -2.107745000 | -1.057100000 |
| C | 4.252567000 | -0.351421000 | 1.312672000 |
| C | 3.121622000 | 0.282616000 | 2.120761000 |
| C | 1.753127000 | -0.169196000 | 1.574053000 |
| C | 1.465139000 | -1.613390000 | 1.981232000 |
| C | 3.235922000 | 2.204148000 | 4.584044000 |
| C | 3.386794000 | 2.572747000 | 3.140772000 |
| C | 3.310545000 | 1.780106000 | 2.074411000 |
| C | 3.415425000 | 2.361866000 | 0.687853000 |
| N | -1.288709000 | 0.739760000 | -1.576325000 |
| C | -2.139159000 | -0.064946000 | -0.677052000 |
| C | -4.127065000 | 0.636308000 | 0.867384000 |
| N | -5.032530000 | -0.478525000 | 0.583363000 |
| C | -5.596531000 | -0.099938000 | -0.715763000 |
| C | -4.471811000 | 0.642400000 | -1.499045000 |
| C | -3.358820000 | 0.882258000 | -0.445107000 |

| | | | |
|---|---|---|---|
| C | -2.692514000 | 2.231431000 | -0.561736000 |
| C | -3.112226000 | 3.472711000 | -0.126668000 |
| C | -2.316971000 | 4.586345000 | -0.392664000 |
| C | -1.126410000 | 4.445556000 | -1.090646000 |
| C | -0.693442000 | 3.199281000 | -1.539386000 |
| C | -1.493157000 | 2.103253000 | -1.261180000 |
| C | -3.250061000 | 0.395543000 | 2.069417000 |
| C | -2.360835000 | -0.808655000 | 1.778741000 |
| C | -1.397026000 | -0.436712000 | 0.626014000 |
| C | -0.355740000 | -1.549240000 | 0.412146000 |
| C | -2.198932000 | -3.592334000 | 3.200561000 |
| C | -3.195891000 | -3.154245000 | 2.174386000 |
| C | -3.268633000 | -1.993292000 | 1.524623000 |
| C | -4.363819000 | -1.790048000 | 0.500055000 |
| C | -0.680120000 | 0.319171000 | -2.728114000 |
| C | -0.853581000 | -1.118308000 | -3.151597000 |
| H | 1.063122000 | 0.899959000 | -0.186859000 |
| H | 4.992363000 | -0.259701000 | -0.692659000 |
| H | 3.773265000 | 2.934288000 | -1.733755000 |
| H | 4.927042000 | 1.682894000 | -2.184414000 |
| H | 3.098830000 | 0.442551000 | -2.945761000 |
| H | 1.933338000 | 1.409880000 | -2.081413000 |
| H | 4.696212000 | -1.905382000 | -2.254982000 |
| H | 4.155271000 | -4.236582000 | -2.897361000 |
| H | 1.952730000 | -5.189211000 | -2.340365000 |
| H | 0.257808000 | -3.851597000 | -1.161855000 |
| H | 5.215749000 | -0.093156000 | 1.750911000 |
| H | 4.174184000 | -1.438525000 | 1.301459000 |
| H | 3.184145000 | -0.056530000 | 3.152668000 |
| H | 0.991985000 | 0.447334000 | 2.057701000 |
| H | 2.171998000 | -2.297373000 | 1.504349000 |
| H | 1.564481000 | -1.725364000 | 3.059631000 |
| H | 4.159844000 | 2.401654000 | 5.129048000 |
| H | 2.462476000 | 2.816683000 | 5.048149000 |
| H | 2.970498000 | 1.162191000 | 4.735222000 |
| H | 3.571084000 | 3.626918000 | 2.957757000 |
| H | 2.408333000 | 2.392236000 | 0.244423000 |
| H | 3.753568000 | 3.395320000 | 0.745522000 |
| H | -2.443994000 | -0.963096000 | -1.199506000 |
| H | -4.762435000 | 1.509645000 | 1.036538000 |
| H | -6.433882000 | 0.576099000 | -0.549220000 |
| H | -5.974198000 | -0.976490000 | -1.236680000 |
| H | -4.838645000 | 1.588292000 | -1.890940000 |
| H | -4.096690000 | 0.066769000 | -2.344312000 |

| | | | |
|---|---|---|---|
| H | -4.045183000 | 3.586114000 | 0.409081000 |
| H | -2.629686000 | 5.563105000 | -0.053482000 |
| H | -0.518389000 | 5.315897000 | -1.291801000 |
| H | 0.224702000 | 3.090835000 | -2.092242000 |
| H | -3.870373000 | 0.209095000 | 2.945240000 |
| H | -2.643405000 | 1.281152000 | 2.265161000 |
| H | -1.741061000 | -1.014788000 | 2.643636000 |
| H | -0.874690000 | 0.464114000 | 0.959362000 |
| H | -0.865241000 | -2.404086000 | -0.039653000 |
| H | -1.656650000 | -4.470290000 | 2.846214000 |
| H | -1.464628000 | -2.829653000 | 3.433900000 |
| H | -2.711296000 | -3.887324000 | 4.116984000 |
| H | -3.957598000 | -3.892562000 | 1.940566000 |
| H | -3.950423000 | -1.947770000 | -0.504678000 |
| H | -5.123081000 | -2.559836000 | 0.630871000 |
| H | 0.002877000 | -1.387388000 | -3.762232000 |
| H | -0.940133000 | -1.820097000 | -2.331863000 |
| H | -1.751769000 | -1.183912000 | -3.767494000 |
| O | -0.062916000 | 1.097517000 | -3.433747000 |
| O | 0.136899000 | -2.009151000 | 1.672155000 |

Compound: **7b**

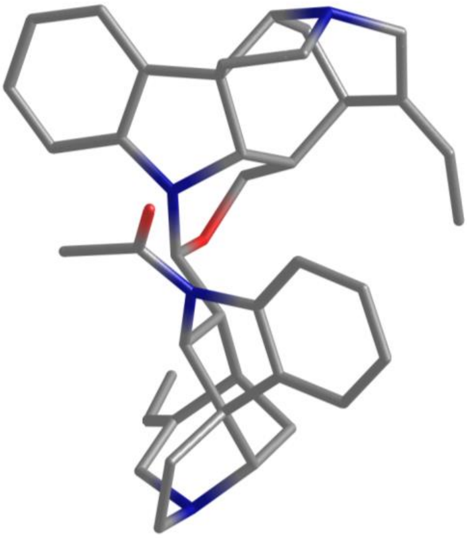

symmetry c1

| | | | |
|---|---|---|---|
| N | 0.861990000 | -1.141609000 | -0.426999000 |
| C | 1.640706000 | -0.006342000 | 0.127680000 |
| C | 4.235250000 | 0.331722000 | 0.220053000 |
| N | 4.305876000 | 1.804508000 | 0.078160000 |
| C | 3.388100000 | 2.214977000 | -0.993130000 |
| C | 3.076449000 | 0.930069000 | -1.747343000 |
| C | 3.012571000 | -0.103999000 | -0.610878000 |
| C | 3.029990000 | -1.530461000 | -1.087622000 |
| C | 4.057121000 | -2.271912000 | -1.634974000 |
| C | 3.816881000 | -3.589881000 | -2.024262000 |
| C | 2.554722000 | -4.140171000 | -1.858359000 |
| C | 1.507531000 | -3.399006000 | -1.315172000 |
| C | 1.758266000 | -2.085952000 | -0.943172000 |
| C | 4.174871000 | -0.099355000 | 1.675239000 |
| C | 2.919865000 | 0.498313000 | 2.309640000 |
| C | 1.669854000 | -0.123622000 | 1.669936000 |
| C | 1.465637000 | -1.582283000 | 2.066235000 |
| C | 0.886064000 | 2.462121000 | 3.553170000 |
| C | 2.031322000 | 2.835613000 | 2.660144000 |
| C | 2.939786000 | 2.014162000 | 2.140365000 |
| C | 4.102965000 | 2.533814000 | 1.327693000 |
| N | -1.158367000 | 0.640125000 | -1.666547000 |
| C | -2.032769000 | -0.163875000 | -0.792114000 |
| C | -4.074185000 | 0.529733000 | 0.682974000 |
| N | -4.964527000 | -0.585899000 | 0.357677000 |
| C | -5.480898000 | -0.200390000 | -0.959282000 |
| C | -4.331770000 | 0.554831000 | -1.693657000 |
| C | -3.258001000 | 0.785090000 | -0.598395000 |

| | | | |
|---|---|---|---|
| C | -2.580262000 | 2.131964000 | -0.681796000 |
| C | -2.998060000 | 3.371332000 | -0.239922000 |
| C | -2.181694000 | 4.479177000 | -0.465155000 |
| C | -0.972534000 | 4.335249000 | -1.130051000 |
| C | -0.540914000 | 3.090874000 | -1.585873000 |
| C | -1.361763000 | 2.001576000 | -1.347572000 |
| C | -3.245224000 | 0.281415000 | 1.917202000 |
| C | -2.334967000 | -0.912360000 | 1.648703000 |
| C | -1.332837000 | -0.516210000 | 0.538135000 |
| C | -0.245072000 | -1.592899000 | 0.369909000 |
| C | -2.183669000 | -3.719465000 | 3.024435000 |
| C | -3.155690000 | -3.273128000 | 1.978248000 |
| C | -3.222200000 | -2.101798000 | 1.346628000 |
| C | -4.290186000 | -1.895869000 | 0.292600000 |
| C | -0.494367000 | 0.213057000 | -2.781689000 |
| C | -0.636990000 | -1.232502000 | -3.188053000 |
| H | 1.148112000 | 0.928379000 | -0.148063000 |
| H | 5.117183000 | -0.110764000 | -0.249741000 |
| H | 2.466951000 | 2.640723000 | -0.574412000 |
| H | 3.842169000 | 2.975027000 | -1.628086000 |
| H | 3.899335000 | 0.669317000 | -2.414507000 |
| H | 2.160002000 | 0.980912000 | -2.331273000 |
| H | 5.041570000 | -1.840158000 | -1.759598000 |
| H | 4.614112000 | -4.182720000 | -2.447577000 |
| H | 2.375203000 | -5.165197000 | -2.150450000 |
| H | 0.534549000 | -3.850931000 | -1.186231000 |
| H | 5.070808000 | 0.229040000 | 2.200088000 |
| H | 4.164274000 | -1.187414000 | 1.726513000 |
| H | 2.894879000 | 0.260004000 | 3.376085000 |
| H | 0.807039000 | 0.413736000 | 2.059156000 |
| H | 2.253497000 | -2.218972000 | 1.656710000 |
| H | 1.479509000 | -1.683696000 | 3.151123000 |
| H | 0.792726000 | 3.180255000 | 4.366647000 |
| H | -0.061612000 | 2.476661000 | 3.009265000 |
| H | 1.007927000 | 1.472683000 | 3.990019000 |
| H | 2.130897000 | 3.894248000 | 2.442324000 |
| H | 3.963083000 | 3.587782000 | 1.093241000 |
| H | 5.015675000 | 2.467020000 | 1.924195000 |
| H | -2.317777000 | -1.066453000 | -1.318527000 |
| H | -4.718032000 | 1.400461000 | 0.833352000 |
| H | -6.328085000 | 0.469440000 | -0.819716000 |
| H | -5.833302000 | -1.074955000 | -1.500771000 |
| H | -4.687874000 | 1.504546000 | -2.086286000 |
| H | -3.924994000 | -0.008699000 | -2.532387000 |

| | | | |
|---|---|---|---|
| H | -3.944263000 | 3.487839000 | 0.271362000 |
| H | -2.492779000 | 5.454266000 | -0.119676000 |
| H | -0.348717000 | 5.201304000 | -1.298980000 |
| H | 0.392721000 | 2.975973000 | -2.111278000 |
| H | -3.899948000 | 0.080144000 | 2.764201000 |
| H | -2.654272000 | 1.169154000 | 2.148579000 |
| H | -1.747647000 | -1.123129000 | 2.535316000 |
| H | -0.858247000 | 0.404622000 | 0.886018000 |
| H | -0.701898000 | -2.456008000 | -0.119884000 |
| H | -1.618135000 | -4.581702000 | 2.667693000 |
| H | -1.467401000 | -2.952125000 | 3.295462000 |
| H | -2.718682000 | -4.041537000 | 3.918484000 |
| H | -3.902111000 | -4.015139000 | 1.709348000 |
| H | -3.850189000 | -2.051988000 | -0.700744000 |
| H | -5.051081000 | -2.667420000 | 0.401883000 |
| H | -1.547815000 | -1.337820000 | -3.779302000 |
| H | 0.213108000 | -1.483989000 | -3.814045000 |
| H | -0.682527000 | -1.921518000 | -2.353645000 |
| O | 0.149645000 | 0.988364000 | -3.467987000 |
| O | 0.197088000 | -2.068708000 | 1.642858000 |

Compound: **7c**

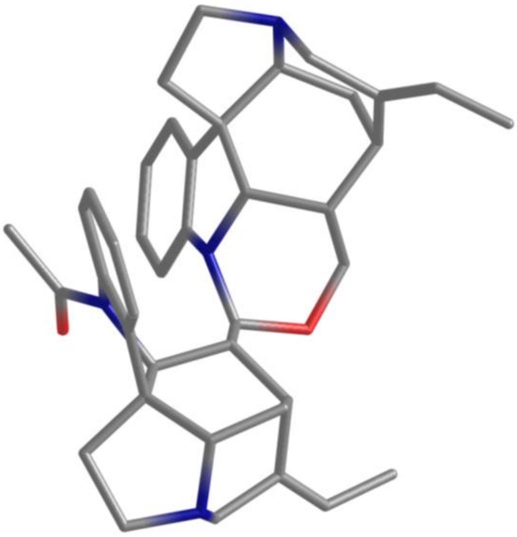

symmetry c1

| | | | |
|---|---|---|---|
| N | 0.765855000 | 0.281580000 | 1.128755000 |
| C | 1.600499000 | 0.012067000 | -0.062044000 |
| C | 4.202283000 | -0.252878000 | -0.202346000 |
| N | 4.331797000 | -1.181300000 | -1.326683000 |
| C | 4.017468000 | -2.466841000 | -0.701044000 |
| C | 2.891840000 | -2.190676000 | 0.336305000 |
| C | 2.892031000 | -0.647109000 | 0.525603000 |
| C | 2.840753000 | -0.213119000 | 1.969064000 |
| C | 3.802515000 | -0.312179000 | 2.955008000 |
| C | 3.514096000 | 0.149897000 | 4.238230000 |
| C | 2.266170000 | 0.694407000 | 4.511450000 |
| C | 1.284776000 | 0.788826000 | 3.529623000 |
| C | 1.587152000 | 0.326923000 | 2.256037000 |
| C | 4.278558000 | 1.196351000 | -0.618671000 |
| C | 3.132251000 | 1.485671000 | -1.587467000 |
| C | 1.774813000 | 1.327949000 | -0.873848000 |
| C | 1.514667000 | 2.516482000 | 0.050758000 |
| C | 3.209260000 | 2.333553000 | -4.595745000 |
| C | 3.354312000 | 0.950406000 | -4.041277000 |
| C | 3.292735000 | 0.560648000 | -2.770164000 |
| C | 3.387901000 | -0.902935000 | -2.421552000 |
| N | -1.532652000 | -1.591209000 | 0.834005000 |
| C | -2.260642000 | -0.334136000 | 0.582255000 |
| C | -3.995209000 | 0.316002000 | -1.239110000 |
| N | -4.936482000 | 1.055768000 | -0.395326000 |
| C | -5.684424000 | -0.020385000 | 0.259687000 |
| C | -4.680855000 | -1.187390000 | 0.497920000 |
| C | -3.428535000 | -0.796839000 | -0.332515000 |
| C | -2.789293000 | -1.970038000 | -1.034705000 |

| | | | |
|---|---|---|---|
| C | -3.148533000 | -2.603292000 | -2.206522000 |
| C | -2.402180000 | -3.695226000 | -2.647771000 |
| C | -1.311496000 | -4.132606000 | -1.913632000 |
| C | -0.937656000 | -3.502034000 | -0.727863000 |
| C | -1.695278000 | -2.426456000 | -0.291668000 |
| C | -2.955838000 | 1.208336000 | -1.872924000 |
| C | -2.123140000 | 1.847187000 | -0.763074000 |
| C | -1.338063000 | 0.716077000 | -0.063204000 |
| C | -0.300950000 | 1.227947000 | 0.958564000 |
| C | -1.789545000 | 4.860310000 | 0.015456000 |
| C | -2.914172000 | 3.945668000 | 0.385695000 |
| C | -3.065784000 | 2.652675000 | 0.103290000 |
| C | -4.292581000 | 1.925341000 | 0.609013000 |
| C | -1.079939000 | -1.878165000 | 2.093641000 |
| C | -0.407818000 | -3.204000000 | 2.318871000 |
| H | 1.084747000 | -0.716836000 | -0.684496000 |
| H | 5.024615000 | -0.481355000 | 0.479699000 |
| H | 3.735877000 | -3.200168000 | -1.453462000 |
| H | 4.908363000 | -2.832074000 | -0.191710000 |
| H | 3.098389000 | -2.698686000 | 1.275979000 |
| H | 1.920861000 | -2.534211000 | -0.018393000 |
| H | 4.771836000 | -0.742901000 | 2.738375000 |
| H | 4.259267000 | 0.085275000 | 5.017319000 |
| H | 2.046335000 | 1.051386000 | 5.507810000 |
| H | 0.312098000 | 1.194536000 | 3.762627000 |
| H | 5.234819000 | 1.387286000 | -1.104043000 |
| H | 4.223258000 | 1.830735000 | 0.265812000 |
| H | 3.201867000 | 2.517439000 | -1.925254000 |
| H | 0.995101000 | 1.353995000 | -1.640041000 |
| H | 2.243700000 | 2.535087000 | 0.865755000 |
| H | 1.608039000 | 3.449292000 | -0.504778000 |
| H | 2.425928000 | 2.352923000 | -5.353912000 |
| H | 2.960827000 | 3.074194000 | -3.841624000 |
| H | 4.129817000 | 2.647276000 | -5.089522000 |
| H | 3.520217000 | 0.177414000 | -4.785451000 |
| H | 2.380647000 | -1.267151000 | -2.169138000 |
| H | 3.706975000 | -1.470313000 | -3.294519000 |
| H | -2.611173000 | 0.025640000 | 1.541714000 |
| H | -4.594756000 | -0.168152000 | -2.014752000 |
| H | -6.478387000 | -0.350496000 | -0.408941000 |
| H | -6.146893000 | 0.339819000 | 1.175475000 |
| H | -5.097980000 | -2.128627000 | 0.146627000 |
| H | -4.426024000 | -1.316033000 | 1.549072000 |
| H | -3.999688000 | -2.259701000 | -2.778967000 |

| | | | |
|---|---|---|---|
| H | -2.669711000 | -4.195521000 | -3.566765000 |
| H | -0.729029000 | -4.971753000 | -2.265643000 |
| H | -0.074089000 | -3.850569000 | -0.185437000 |
| H | -3.450003000 | 1.974700000 | -2.468966000 |
| H | -2.320204000 | 0.620007000 | -2.537045000 |
| H | -1.387982000 | 2.512733000 | -1.202709000 |
| H | -0.802210000 | 0.209110000 | -0.870104000 |
| H | -0.798663000 | 1.364554000 | 1.918055000 |
| H | -1.043314000 | 4.384598000 | -0.611091000 |
| H | -2.177688000 | 5.740309000 | -0.498630000 |
| H | -1.282117000 | 5.214538000 | 0.913859000 |
| H | -3.705976000 | 4.414908000 | 0.962523000 |
| H | -4.025853000 | 1.348247000 | 1.502995000 |
| H | -5.031223000 | 2.655900000 | 0.935696000 |
| H | 0.582552000 | -3.191035000 | 1.863754000 |
| H | -0.299112000 | -3.341429000 | 3.388615000 |
| H | -0.974338000 | -4.026900000 | 1.889760000 |
| O | -1.214940000 | -1.076142000 | 3.000990000 |
| O | 0.202158000 | 2.512344000 | 0.586843000 |

Compound: **7d**

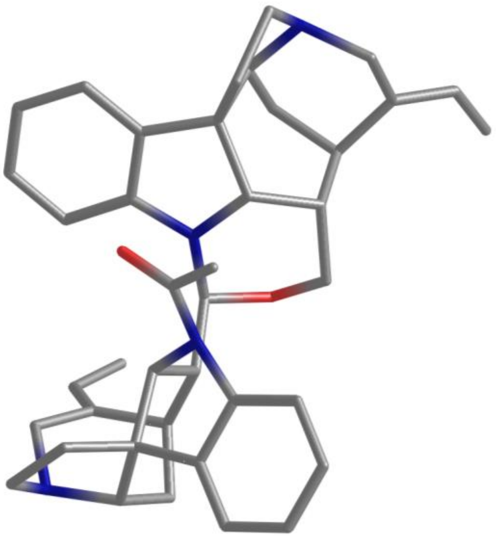

symmetry c1

| | | | |
|---|---|---|---|
| N | 0.777507000 | -0.674332000 | 0.017542000 |
| C | 1.751317000 | 0.366471000 | -0.324527000 |
| C | 4.068455000 | -0.807997000 | -0.524656000 |
| N | 5.116546000 | 0.155347000 | -0.868501000 |
| C | 4.844861000 | 0.407489000 | -2.285860000 |
| C | 3.309117000 | 0.526450000 | -2.406187000 |
| C | 2.763441000 | -0.357779000 | -1.266403000 |
| C | 1.994897000 | -1.590666000 | -1.671093000 |
| C | 2.289693000 | -2.537145000 | -2.624248000 |
| C | 1.486747000 | -3.677121000 | -2.733003000 |
| C | 0.420032000 | -3.850254000 | -1.865566000 |
| C | 0.107844000 | -2.898900000 | -0.893856000 |
| C | 0.899911000 | -1.760033000 | -0.818963000 |
| C | 3.850289000 | -0.992933000 | 0.959369000 |
| C | 3.555710000 | 0.374684000 | 1.579101000 |
| C | 2.316073000 | 1.052626000 | 0.934157000 |
| C | 1.178331000 | 1.192568000 | 1.934991000 |
| C | 5.372959000 | 1.684554000 | 3.782077000 |
| C | 5.562057000 | 1.740978000 | 2.297767000 |
| C | 4.788262000 | 1.213814000 | 1.352776000 |
| C | 5.097413000 | 1.414149000 | -0.106299000 |
| N | -1.433138000 | 1.264504000 | -0.984551000 |
| C | -2.048825000 | 0.095889000 | -0.309004000 |
| C | -4.393875000 | -0.152394000 | 0.843930000 |
| N | -4.728600000 | -1.500477000 | 0.386729000 |
| C | -5.160789000 | -1.264080000 | -0.995145000 |
| C | -4.304865000 | -0.081668000 | -1.545589000 |
| C | -3.567987000 | 0.473675000 | -0.299656000 |
| C | -3.483436000 | 1.979892000 | -0.238655000 |

| | | | |
|---|---|---|---|
| C | -4.430048000 | 2.909377000 | 0.148093000 |
| C | -4.091248000 | 4.260967000 | 0.153554000 |
| C | -2.816059000 | 4.665096000 | -0.212776000 |
| C | -1.855378000 | 3.735860000 | -0.605044000 |
| C | -2.215470000 | 2.400709000 | -0.633542000 |
| C | -3.717846000 | -0.132083000 | 2.192453000 |
| C | -2.387556000 | -0.862254000 | 2.059996000 |
| C | -1.467966000 | -0.064768000 | 1.104320000 |
| C | -0.043107000 | -0.677143000 | 1.196025000 |
| C | -1.214274000 | -3.450799000 | 3.343313000 |
| C | -2.203916000 | -3.368939000 | 2.222892000 |
| C | -2.685468000 | -2.280308000 | 1.624258000 |
| C | -3.590939000 | -2.435691000 | 0.422207000 |
| C | -0.827027000 | 1.106022000 | -2.217043000 |
| C | -0.441984000 | 2.356730000 | -2.966711000 |
| H | 1.247155000 | 1.147583000 | -0.903808000 |
| H | 4.368720000 | -1.755350000 | -0.975942000 |
| H | 5.376516000 | 1.292056000 | -2.630456000 |
| H | 5.199962000 | -0.447113000 | -2.863168000 |
| H | 2.941712000 | 0.207054000 | -3.378970000 |
| H | 2.990674000 | 1.560141000 | -2.265080000 |
| H | 3.141019000 | -2.404604000 | -3.281398000 |
| H | 1.702113000 | -4.422647000 | -3.484063000 |
| H | -0.193610000 | -4.737068000 | -1.944658000 |
| H | -0.737498000 | -3.043964000 | -0.234824000 |
| H | 4.738514000 | -1.420013000 | 1.423635000 |
| H | 3.021338000 | -1.684217000 | 1.117386000 |
| H | 3.368666000 | 0.245959000 | 2.641858000 |
| H | 2.587265000 | 2.068548000 | 0.646106000 |
| H | 1.533334000 | 1.638991000 | 2.861806000 |
| H | 0.397664000 | 1.840548000 | 1.519943000 |
| H | 4.451769000 | 1.189474000 | 4.074761000 |
| H | 6.203614000 | 1.157950000 | 4.253651000 |
| H | 5.362679000 | 2.691100000 | 4.201328000 |
| H | 6.442009000 | 2.284987000 | 1.968176000 |
| H | 4.352797000 | 2.107766000 | -0.524423000 |
| H | 6.067670000 | 1.895412000 | -0.222040000 |
| H | -1.835726000 | -0.771942000 | -0.923388000 |
| H | -5.338741000 | 0.395179000 | 0.893700000 |
| H | -6.212749000 | -0.982762000 | -0.987607000 |
| H | -5.062000000 | -2.172331000 | -1.584509000 |
| H | -4.946729000 | 0.679260000 | -1.984668000 |
| H | -3.596525000 | -0.395331000 | -2.311056000 |
| H | -5.421944000 | 2.598469000 | 0.447060000 |

| | | | |
|---|---|---|---|
| H | -4.820991000 | 4.996514000 | 0.458818000 |
| H | -2.555775000 | 5.713225000 | -0.185016000 |
| H | -0.860027000 | 4.057858000 | -0.868713000 |
| H | -4.355102000 | -0.618548000 | 2.930005000 |
| H | -3.557658000 | 0.899500000 | 2.509092000 |
| H | -1.887209000 | -0.877692000 | 3.025686000 |
| H | -1.418777000 | 0.940053000 | 1.530091000 |
| H | -0.157763000 | -1.725308000 | 1.477176000 |
| H | -1.642651000 | -3.976646000 | 4.197039000 |
| H | -0.343123000 | -4.026480000 | 3.025864000 |
| H | -0.863107000 | -2.479977000 | 3.681847000 |
| H | -2.541919000 | -4.329758000 | 1.845947000 |
| H | -2.982350000 | -2.330680000 | -0.486474000 |
| H | -3.987126000 | -3.450176000 | 0.399834000 |
| H | -1.300294000 | 3.006876000 | -3.121583000 |
| H | 0.309125000 | 2.919789000 | -2.415194000 |
| H | -0.030756000 | 2.051997000 | -3.922200000 |
| O | -0.584623000 | 0.006354000 | -2.669485000 |
| O | 0.642566000 | -0.073763000 | 2.277632000 |

Compound: **8a**

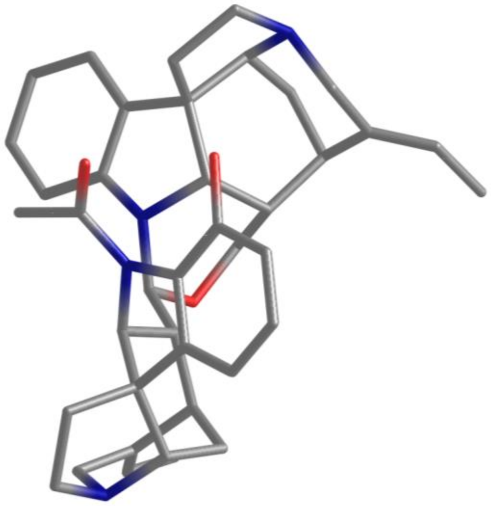

symmetry c1

| | | | |
|---|---|---|---|
| N | 0.716670000 | -1.157699000 | -0.529547000 |
| C | 1.594766000 | -0.110387000 | 0.045286000 |
| C | 4.206318000 | 0.073261000 | -0.066960000 |
| N | 4.377884000 | 1.526101000 | -0.042644000 |
| C | 4.072258000 | 1.897996000 | -1.426432000 |
| C | 2.938995000 | 0.945712000 | -1.903475000 |
| C | 2.886576000 | -0.182944000 | -0.838359000 |
| C | 2.785298000 | -1.562843000 | -1.436045000 |
| C | 3.718775000 | -2.285667000 | -2.151051000 |
| C | 3.385513000 | -3.554765000 | -2.624267000 |
| C | 2.124495000 | -4.075938000 | -2.374729000 |
| C | 1.170395000 | -3.352031000 | -1.662951000 |
| C | 1.514196000 | -2.087455000 | -1.206594000 |
| C | 4.268869000 | -0.553931000 | 1.304686000 |
| C | 3.138551000 | 0.026022000 | 2.153445000 |
| C | 1.768689000 | -0.371537000 | 1.568853000 |
| C | 1.469966000 | -1.842023000 | 1.853967000 |
| C | 3.259704000 | 1.755018000 | 4.754519000 |
| C | 3.419505000 | 2.229976000 | 3.343612000 |
| C | 3.339718000 | 1.521297000 | 2.219918000 |
| C | 3.454596000 | 2.206050000 | 0.881826000 |
| N | -1.299836000 | 0.909622000 | -1.417600000 |
| C | -2.126655000 | -0.049329000 | -0.647411000 |
| C | -4.198411000 | 0.352160000 | 0.886930000 |
| N | -5.008059000 | -0.787485000 | 0.454176000 |
| C | -5.554507000 | -0.319010000 | -0.823377000 |
| C | -4.473715000 | 0.598760000 | -1.473818000 |
| C | -3.412012000 | 0.791130000 | -0.361568000 |
| C | -2.836137000 | 2.183701000 | -0.281636000 |

| | | | |
|---|---|---|---|
| C | -3.345500000 | 3.312798000 | 0.329310000 |
| C | -2.584291000 | 4.479374000 | 0.298406000 |
| C | -1.353328000 | 4.509272000 | -0.333403000 |
| C | -0.831189000 | 3.374252000 | -0.956667000 |
| C | -1.603077000 | 2.215057000 | -0.923113000 |
| C | -3.334825000 | 0.059120000 | 2.086751000 |
| C | -2.365673000 | -1.057211000 | 1.716478000 |
| C | -1.397865000 | -0.541805000 | 0.621916000 |
| C | -0.354336000 | -1.625611000 | 0.301177000 |
| C | -2.118383000 | -3.925837000 | 2.949066000 |
| C | -3.088475000 | -3.465662000 | 1.907260000 |
| C | -3.193854000 | -2.266061000 | 1.337746000 |
| C | -4.248757000 | -2.038453000 | 0.278642000 |
| C | -0.696708000 | 0.683838000 | -2.606599000 |
| C | -0.670131000 | -0.692903000 | -3.209495000 |
| H | 1.108916000 | 0.854165000 | -0.097843000 |
| H | 5.014840000 | -0.320895000 | -0.687513000 |
| H | 3.795432000 | 2.948218000 | -1.486839000 |
| H | 4.964807000 | 1.747963000 | -2.032626000 |
| H | 3.163757000 | 0.537708000 | -2.886692000 |
| H | 1.982923000 | 1.454052000 | -1.975843000 |
| H | 4.701625000 | -1.875519000 | -2.344561000 |
| H | 4.109901000 | -4.132674000 | -3.178764000 |
| H | 1.872863000 | -5.063690000 | -2.734134000 |
| H | 0.196483000 | -3.779557000 | -1.473673000 |
| H | 5.231665000 | -0.333016000 | 1.763702000 |
| H | 4.185675000 | -1.636985000 | 1.214333000 |
| H | 3.193263000 | -0.390741000 | 3.156855000 |
| H | 1.010554000 | 0.208463000 | 2.099904000 |
| H | 2.172538000 | -2.489393000 | 1.322943000 |
| H | 1.565875000 | -2.044311000 | 2.919555000 |
| H | 4.183353000 | 1.901946000 | 5.315770000 |
| H | 2.490315000 | 2.338233000 | 5.261204000 |
| H | 2.983984000 | 0.707227000 | 4.826165000 |
| H | 3.613965000 | 3.293178000 | 3.241387000 |
| H | 3.807728000 | 3.226814000 | 1.019462000 |
| H | 2.451061000 | 2.287863000 | 0.438738000 |
| H | -2.359370000 | -0.888719000 | -1.289128000 |
| H | -4.902339000 | 1.155104000 | 1.119730000 |
| H | -5.831713000 | -1.163142000 | -1.450137000 |
| H | -6.454986000 | 0.261003000 | -0.627619000 |
| H | -4.905913000 | 1.555495000 | -1.757840000 |
| H | -4.034881000 | 0.163355000 | -2.370830000 |
| H | -4.303788000 | 3.295872000 | 0.828156000 |

| | | | |
|---|---|---|---|
| H | -2.954145000 | 5.374606000 | 0.777119000 |
| H | -0.758798000 | 5.411061000 | -0.354603000 |
| H | -3.958437000 | -0.243890000 | 2.926717000 |
| H | -2.792064000 | 0.960957000 | 2.374675000 |
| H | -1.751747000 | -1.300901000 | 2.575610000 |
| H | -0.889241000 | 0.323046000 | 1.056188000 |
| H | -0.871957000 | -2.431223000 | -0.225539000 |
| H | -2.656592000 | -4.312859000 | 3.815017000 |
| H | -1.512637000 | -4.746050000 | 2.561059000 |
| H | -1.439297000 | -3.145494000 | 3.273603000 |
| H | -3.797697000 | -4.222502000 | 1.584641000 |
| H | -3.776972000 | -2.069590000 | -0.712942000 |
| H | -4.957901000 | -2.864858000 | 0.293772000 |
| H | 0.347894000 | -0.880094000 | -3.544787000 |
| H | -0.970200000 | -1.496733000 | -2.551343000 |
| H | -1.314530000 | -0.674506000 | -4.087988000 |
| O | -0.179375000 | 1.608856000 | -3.241063000 |
| O | 0.139370000 | -2.201344000 | 1.510666000 |
| O | 0.389473000 | 3.436497000 | -1.526712000 |
| H | 0.367281000 | 2.794825000 | -2.279955000 |

Compound: **8b**

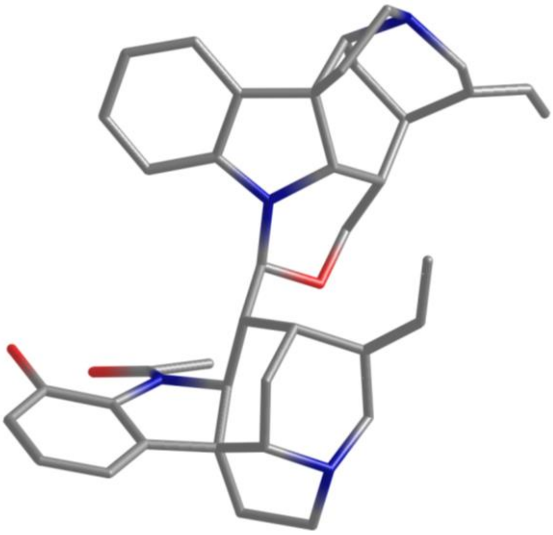

symmetry c1

| | | | |
|---|---|---|---|
| N | -0.967351000 | 0.746943000 | -0.148345000 |
| C | -2.251004000 | 0.070108000 | -0.395928000 |
| C | -4.499687000 | 1.400506000 | -0.090008000 |
| N | -5.527415000 | 0.609994000 | -0.771183000 |
| C | -5.281896000 | 0.946810000 | -2.174483000 |
| C | -3.742919000 | 0.971256000 | -2.345228000 |
| C | -3.191539000 | 1.225715000 | -0.914442000 |
| C | -2.319044000 | 2.451948000 | -0.811698000 |
| C | -2.620808000 | 3.765753000 | -1.094091000 |
| C | -1.646146000 | 4.750452000 | -0.910871000 |
| C | -0.388117000 | 4.395967000 | -0.448594000 |
| C | -0.063602000 | 3.069973000 | -0.165566000 |
| C | -1.048737000 | 2.104478000 | -0.349348000 |
| C | -4.329458000 | 1.068530000 | 1.374732000 |
| C | -4.037908000 | -0.424829000 | 1.510735000 |
| C | -2.688374000 | -0.777780000 | 0.846548000 |
| C | -1.588950000 | -0.726597000 | 1.897865000 |
| C | -5.905847000 | -2.529945000 | 2.912127000 |
| C | -5.973267000 | -2.053706000 | 1.494488000 |
| C | -5.181577000 | -1.177468000 | 0.882919000 |
| C | -5.368721000 | -0.836973000 | -0.566958000 |
| N | 3.089657000 | 0.481342000 | 1.232477000 |
| C | 2.478092000 | -0.711355000 | 0.600001000 |
| C | 3.221252000 | -2.151262000 | -1.406249000 |
| N | 2.769621000 | -3.434710000 | -0.860200000 |
| C | 3.800661000 | -3.760553000 | 0.133112000 |
| C | 4.392261000 | -2.413186000 | 0.650230000 |
| C | 3.666403000 | -1.333826000 | -0.180713000 |
| C | 4.497227000 | -0.112003000 | -0.484146000 |

| | | | |
|---|---|---|---|
| C | 5.468991000 | 0.066515000 | -1.449268000 |
| C | 6.067566000 | 1.319646000 | -1.556971000 |
| C | 5.699582000 | 2.357803000 | -0.719046000 |
| C | 4.716640000 | 2.189940000 | 0.258932000 |
| C | 4.135222000 | 0.928104000 | 0.366121000 |
| C | 2.200365000 | -1.454325000 | -2.278697000 |
| C | 0.931540000 | -1.238358000 | -1.447966000 |
| C | 1.298290000 | -0.273155000 | -0.294392000 |
| C | 0.120312000 | 0.147212000 | 0.579383000 |
| C | -1.624736000 | -2.681156000 | -2.590484000 |
| C | -0.618817000 | -3.224971000 | -1.623372000 |
| C | 0.450693000 | -2.628297000 | -1.100037000 |
| C | 1.416853000 | -3.425547000 | -0.265571000 |
| C | 2.974677000 | 0.834156000 | 2.535093000 |
| C | 2.081432000 | 0.017692000 | 3.431095000 |
| H | -2.114483000 | -0.633790000 | -1.212669000 |
| H | -4.799038000 | 2.445171000 | -0.193796000 |
| H | -5.776441000 | 0.236698000 | -2.833690000 |
| H | -5.696465000 | 1.935865000 | -2.368666000 |
| H | -3.431082000 | 1.751995000 | -3.034557000 |
| H | -3.373540000 | 0.026153000 | -2.740013000 |
| H | -3.604279000 | 4.035367000 | -1.459903000 |
| H | -1.871387000 | 5.783009000 | -1.132250000 |
| H | 0.366075000 | 5.158008000 | -0.310929000 |
| H | 0.931172000 | 2.813416000 | 0.172442000 |
| H | -5.232849000 | 1.324749000 | 1.926156000 |
| H | -3.509394000 | 1.667112000 | 1.778760000 |
| H | -3.962793000 | -0.678921000 | 2.566707000 |
| H | -2.736016000 | -1.815860000 | 0.511880000 |
| H | -1.573550000 | 0.243814000 | 2.409751000 |
| H | -1.741984000 | -1.504979000 | 2.641854000 |
| H | -6.824305000 | -2.276743000 | 3.442840000 |
| H | -5.816381000 | -3.616237000 | 2.942319000 |
| H | -5.071389000 | -2.109268000 | 3.465145000 |
| H | -6.771889000 | -2.490572000 | 0.902870000 |
| H | -4.498260000 | -1.219917000 | -1.127719000 |
| H | -6.247427000 | -1.336957000 | -0.970599000 |
| H | 2.131457000 | -1.374443000 | 1.381744000 |
| H | 4.119891000 | -2.363624000 | -1.992054000 |
| H | 3.375168000 | -4.369602000 | 0.927220000 |
| H | 4.586760000 | -4.343095000 | -0.344938000 |
| H | 5.462558000 | -2.370827000 | 0.459302000 |
| H | 4.246823000 | -2.263808000 | 1.719325000 |
| H | 5.753153000 | -0.738961000 | -2.111439000 |

| | | | |
|---|---|---|---|
| H | 6.825260000 | 1.490577000 | -2.308034000 |
| H | 6.155529000 | 3.333225000 | -0.807406000 |
| H | 1.978005000 | -2.063583000 | -3.153149000 |
| H | 2.611062000 | -0.502971000 | -2.620718000 |
| H | 0.183472000 | -0.722281000 | -2.046154000 |
| H | 1.625055000 | 0.642754000 | -0.797985000 |
| H | 0.472538000 | 0.910983000 | 1.290920000 |
| H | -1.370819000 | -1.693953000 | -2.967007000 |
| H | -1.703795000 | -3.350383000 | -3.447600000 |
| H | -2.620315000 | -2.634742000 | -2.142255000 |
| H | -0.775860000 | -4.264273000 | -1.352819000 |
| H | 1.423625000 | -3.036905000 | 0.759180000 |
| H | 1.079893000 | -4.458971000 | -0.200135000 |
| H | 2.616349000 | -0.889374000 | 3.716748000 |
| H | 1.888389000 | 0.600554000 | 4.324434000 |
| H | 1.148254000 | -0.283158000 | 2.965983000 |
| O | 3.611456000 | 1.778429000 | 3.003828000 |
| O | -0.331614000 | -0.973241000 | 1.294372000 |
| O | 4.356078000 | 3.246791000 | 1.011670000 |
| H | 4.022868000 | 2.884290000 | 1.867175000 |

Compound: **8c**

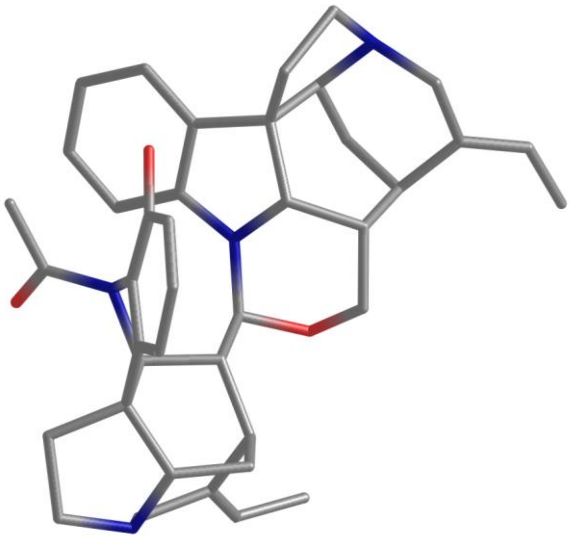

symmetry c1

| | | | |
|---|---|---|---|
| N | -0.855606000 | 0.119688000 | -0.849336000 |
| C | -1.695069000 | -0.027618000 | 0.344248000 |
| C | -4.043232000 | -0.646450000 | -0.596507000 |
| N | -5.033008000 | -0.759918000 | 0.474182000 |
| C | -4.673032000 | -2.037409000 | 1.090990000 |
| C | -3.130141000 | -2.055278000 | 1.159333000 |
| C | -2.677824000 | -1.178049000 | -0.027010000 |
| C | -1.937491000 | -1.854457000 | -1.155556000 |
| C | -2.210795000 | -3.048656000 | -1.791911000 |
| C | -1.520543000 | -3.378201000 | -2.959344000 |
| C | -0.587863000 | -2.488408000 | -3.476668000 |
| C | -0.296624000 | -1.283417000 | -2.844422000 |
| C | -0.963191000 | -0.981592000 | -1.662907000 |
| C | -3.923577000 | 0.738772000 | -1.185128000 |
| C | -3.605963000 | 1.714266000 | -0.050092000 |
| C | -2.315961000 | 1.328793000 | 0.729776000 |
| C | -1.248673000 | 2.412989000 | 0.599433000 |
| C | -5.474581000 | 4.125454000 | 0.703178000 |
| C | -5.595208000 | 2.705524000 | 1.162642000 |
| C | -4.797349000 | 1.680991000 | 0.874374000 |
| C | -5.030518000 | 0.320362000 | 1.474147000 |
| N | 1.757686000 | -1.365228000 | -0.293751000 |
| C | 2.318789000 | 0.002533000 | -0.342735000 |
| C | 3.955405000 | 1.269234000 | 1.258877000 |
| N | 4.894443000 | 1.779020000 | 0.258327000 |
| C | 5.729463000 | 0.604284000 | -0.006281000 |
| C | 4.790447000 | -0.635979000 | 0.067049000 |
| C | 3.482691000 | -0.090396000 | 0.704258000 |

| | | | |
|---|---|---|---|
| C | 2.852305000 | -1.028973000 | 1.711709000 |
| C | 3.102616000 | -1.213398000 | 3.056579000 |
| C | 2.293405000 | -2.105822000 | 3.762385000 |
| C | 1.242029000 | -2.764420000 | 3.147763000 |
| C | 0.979149000 | -2.567347000 | 1.791188000 |
| C | 1.833626000 | -1.740982000 | 1.082639000 |
| C | 2.856308000 | 2.255924000 | 1.564209000 |
| C | 2.004430000 | 2.424381000 | 0.308572000 |
| C | 1.289912000 | 1.070705000 | 0.052289000 |
| C | 0.092243000 | 1.201965000 | -0.923084000 |
| C | 1.389104000 | 4.820454000 | -1.599389000 |
| C | 2.631803000 | 3.988050000 | -1.563222000 |
| C | 2.910562000 | 2.934251000 | -0.796495000 |
| C | 4.244940000 | 2.235804000 | -0.986827000 |
| C | 2.106945000 | -2.238962000 | -1.318338000 |
| C | 1.782151000 | -3.693786000 | -1.120818000 |
| H | -1.074040000 | -0.367586000 | 1.186957000 |
| H | -4.349863000 | -1.354713000 | -1.368373000 |
| H | -5.149943000 | -2.147369000 | 2.062551000 |
| H | -5.026727000 | -2.844245000 | 0.447636000 |
| H | -2.728797000 | -3.064024000 | 1.101888000 |
| H | -2.775692000 | -1.627620000 | 2.097851000 |
| H | -2.967365000 | -3.714817000 | -1.394980000 |
| H | -1.716658000 | -4.313504000 | -3.461853000 |
| H | -0.055689000 | -2.740028000 | -4.383423000 |
| H | 0.461994000 | -0.629618000 | -3.247328000 |
| H | -4.860287000 | 1.028897000 | -1.659932000 |
| H | -3.143598000 | 0.744966000 | -1.945666000 |
| H | -3.474262000 | 2.707781000 | -0.469770000 |
| H | -2.555673000 | 1.281227000 | 1.792099000 |
| H | -1.700132000 | 3.398148000 | 0.705109000 |
| H | -0.506154000 | 2.305165000 | 1.394986000 |
| H | -4.585980000 | 4.304936000 | 0.105147000 |
| H | -6.344122000 | 4.410643000 | 0.109758000 |
| H | -5.446040000 | 4.799369000 | 1.559903000 |
| H | -6.440510000 | 2.504475000 | 1.813693000 |
| H | -5.985982000 | 0.299907000 | 1.996587000 |
| H | -4.256223000 | 0.139942000 | 2.234095000 |
| H | 2.668817000 | 0.165562000 | -1.353690000 |
| H | 4.543004000 | 1.070646000 | 2.159244000 |
| H | 6.233257000 | 0.701509000 | -0.964841000 |
| H | 6.491906000 | 0.538103000 | 0.768599000 |
| H | 5.228181000 | -1.413181000 | 0.689941000 |
| H | 4.596394000 | -1.068436000 | -0.912298000 |

| | | | |
|---|---|---|---|
| H | 3.892171000 | -0.674354000 | 3.560683000 |
| H | 2.468073000 | -2.266728000 | 4.816356000 |
| H | 0.590960000 | -3.420879000 | 3.706537000 |
| H | 3.298416000 | 3.206192000 | 1.861562000 |
| H | 2.244930000 | 1.893135000 | 2.392051000 |
| H | 1.244890000 | 3.174305000 | 0.494747000 |
| H | 0.883247000 | 0.762031000 | 1.018874000 |
| H | 0.455349000 | 1.280011000 | -1.948490000 |
| H | 0.944456000 | 4.785167000 | -2.595185000 |
| H | 0.633548000 | 4.491758000 | -0.894542000 |
| H | 1.631585000 | 5.865482000 | -1.402603000 |
| H | 3.401479000 | 4.297864000 | -2.264331000 |
| H | 4.113976000 | 1.393197000 | -1.675866000 |
| H | 4.929804000 | 2.920493000 | -1.484947000 |
| H | 2.012542000 | -4.045236000 | -0.119018000 |
| H | 0.716746000 | -3.843475000 | -1.307897000 |
| H | 2.339121000 | -4.262251000 | -1.857265000 |
| O | 2.603630000 | -1.837709000 | -2.351092000 |
| O | -0.620600000 | 2.402713000 | -0.672741000 |
| O | -0.096002000 | -3.180870000 | 1.237906000 |
| H | -0.286068000 | -2.811652000 | 0.363498000 |

Compound: **9a**

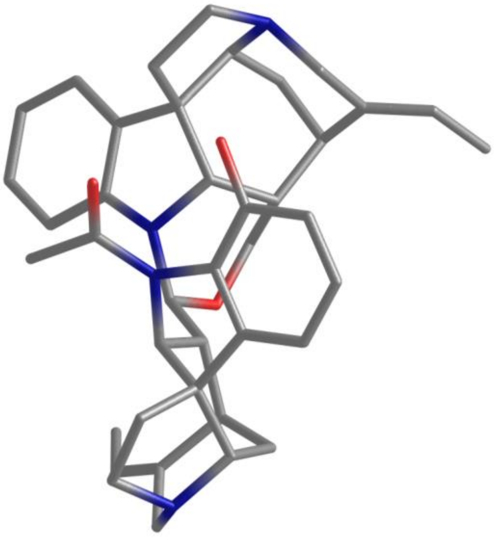

symmetry c1

| | | | |
|---|---|---|---|
| N | -0.607313000 | 1.300173000 | -0.238083000 |
| C | -1.544411000 | 0.188394000 | 0.067162000 |
| C | -4.154844000 | 0.128633000 | -0.050663000 |
| N | -4.339952000 | -1.280764000 | -0.403644000 |
| C | -4.022169000 | -1.302847000 | -1.836126000 |
| C | -2.958049000 | -0.194943000 | -2.082082000 |
| C | -2.837719000 | 0.560496000 | -0.735068000 |
| C | -2.672567000 | 2.044060000 | -0.931175000 |
| C | -3.580780000 | 2.981519000 | -1.379496000 |
| C | -3.178910000 | 4.309015000 | -1.525379000 |
| C | -1.873307000 | 4.670742000 | -1.229684000 |
| C | -0.946137000 | 3.731290000 | -0.783698000 |
| C | -1.361213000 | 2.414755000 | -0.639275000 |
| C | -4.204677000 | 0.381493000 | 1.436544000 |
| C | -3.086403000 | -0.422502000 | 2.098027000 |
| C | -1.706927000 | 0.062506000 | 1.608529000 |
| C | -1.360102000 | 1.404797000 | 2.245917000 |
| C | -3.290376000 | -2.734784000 | 4.194546000 |
| C | -3.451640000 | -2.840471000 | 2.709637000 |
| C | -3.337246000 | -1.879533000 | 1.796035000 |
| C | -3.456254000 | -2.204038000 | 0.330037000 |
| N | 1.250995000 | -0.656784000 | -1.491967000 |
| C | 2.181127000 | 0.004729000 | -0.549208000 |
| C | 4.128203000 | -0.963369000 | 0.885838000 |
| N | 5.156441000 | 0.022993000 | 0.494492000 |
| C | 5.176543000 | 0.136092000 | -0.970352000 |
| C | 4.302322000 | -1.011838000 | -1.466631000 |

| | | | |
|---|---|---|---|
| C | 3.246568000 | -1.112957000 | -0.354922000 |
| C | 2.398830000 | -2.352173000 | -0.451985000 |
| C | 2.619826000 | -3.621514000 | 0.041563000 |
| C | 1.659918000 | -4.600664000 | -0.209501000 |
| C | 0.528308000 | -4.310936000 | -0.950097000 |
| C | 0.299635000 | -3.029966000 | -1.459406000 |
| C | 1.253389000 | -2.052833000 | -1.184497000 |
| C | 3.370091000 | -0.534378000 | 2.130052000 |
| C | 2.611693000 | 0.758296000 | 1.824443000 |
| C | 1.514847000 | 0.400257000 | 0.775101000 |
| C | 0.474727000 | 1.525071000 | 0.679832000 |
| C | 2.280825000 | 3.949919000 | 1.865753000 |
| C | 3.455302000 | 3.144455000 | 1.406079000 |
| C | 3.631887000 | 1.822650000 | 1.407976000 |
| C | 5.044592000 | 1.294848000 | 1.193233000 |
| C | 0.799928000 | -0.124783000 | -2.648410000 |
| C | 1.126017000 | 1.310133000 | -2.959509000 |
| H | -1.116823000 | -0.731741000 | -0.327283000 |
| H | -4.960847000 | 0.678048000 | -0.544689000 |
| H | -3.674122000 | -2.290656000 | -2.129162000 |
| H | -4.925571000 | -1.079585000 | -2.402331000 |
| H | -3.299712000 | 0.490417000 | -2.855239000 |
| H | -2.000701000 | -0.588740000 | -2.402750000 |
| H | -4.596375000 | 2.692808000 | -1.617742000 |
| H | -3.882812000 | 5.053325000 | -1.867042000 |
| H | -1.563985000 | 5.700255000 | -1.341516000 |
| H | 0.063777000 | 4.039061000 | -0.555256000 |
| H | -5.171057000 | 0.070426000 | 1.831571000 |
| H | -4.096761000 | 1.449441000 | 1.627123000 |
| H | -3.118085000 | -0.264404000 | 3.173934000 |
| H | -0.969398000 | -0.657486000 | 1.969358000 |
| H | -2.035570000 | 2.187713000 | 1.891986000 |
| H | -1.455139000 | 1.342543000 | 3.328523000 |
| H | -2.988532000 | -1.745180000 | 4.524146000 |
| H | -4.221723000 | -2.991360000 | 4.700983000 |
| H | -2.539975000 | -3.445648000 | 4.541715000 |
| H | -3.679721000 | -3.838105000 | 2.346989000 |
| H | -2.451006000 | -2.214418000 | -0.113442000 |
| H | -3.849428000 | -3.212380000 | 0.206724000 |
| H | 2.614875000 | 0.883049000 | -1.019700000 |
| H | 4.610802000 | -1.927465000 | 1.066023000 |
| H | 6.193461000 | 0.077007000 | -1.356290000 |
| H | 4.762412000 | 1.098853000 | -1.291122000 |
| H | 4.865543000 | -1.943802000 | -1.507596000 |

| | | | |
|---|---|---|---|
| H | 3.869499000 | -0.832276000 | -2.449849000 |
| H | 3.507550000 | -3.853390000 | 0.611894000 |
| H | 1.798548000 | -5.600832000 | 0.174818000 |
| H | -0.214779000 | -5.068629000 | -1.153005000 |
| H | 4.063108000 | -0.399773000 | 2.959366000 |
| H | 2.675018000 | -1.322971000 | 2.418294000 |
| H | 2.087360000 | 1.098256000 | 2.714671000 |
| H | 1.004540000 | -0.485323000 | 1.163178000 |
| H | 0.995217000 | 2.426132000 | 0.354280000 |
| H | 2.618973000 | 4.688965000 | 2.593032000 |
| H | 1.838837000 | 4.515897000 | 1.042953000 |
| H | 1.503433000 | 3.347702000 | 2.324486000 |
| H | 4.312580000 | 3.738614000 | 1.101291000 |
| H | 5.645244000 | 2.032605000 | 0.664728000 |
| H | 5.501133000 | 1.185480000 | 2.180442000 |
| H | 2.156746000 | 1.374227000 | -3.311205000 |
| H | 0.464658000 | 1.635824000 | -3.754869000 |
| H | 1.010433000 | 1.959894000 | -2.098709000 |
| O | 0.164648000 | -0.801681000 | -3.461722000 |
| O | -0.015082000 | 1.786594000 | 2.000679000 |
| O | -0.829095000 | -2.796993000 | -2.158678000 |
| H | -0.621659000 | -2.052781000 | -2.776373000 |

Compound: **9b**

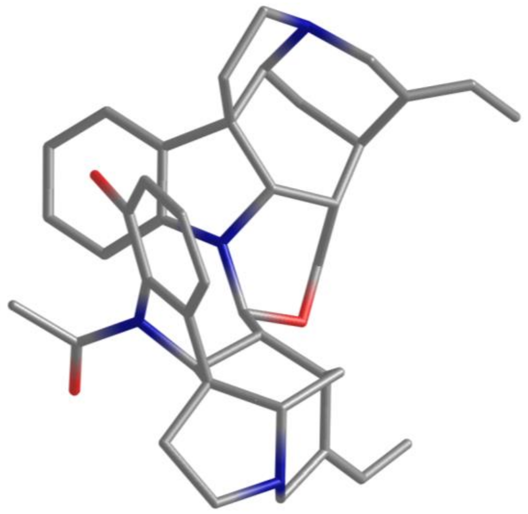

symmetry c1

| | | | |
|---|---|---|---|
| N | -0.716832000 | -0.207914000 | -0.865132000 |
| C | -1.761349000 | 0.147082000 | 0.108130000 |
| C | -4.196712000 | -0.825635000 | 0.051513000 |
| N | -4.666820000 | -0.620859000 | 1.422285000 |
| C | -4.018196000 | -1.722641000 | 2.135901000 |
| C | -2.601742000 | -1.877990000 | 1.517489000 |
| C | -2.677669000 | -1.128271000 | 0.157881000 |
| C | -2.183455000 | -1.940291000 | -1.012571000 |
| C | -2.664516000 | -3.132471000 | -1.511047000 |
| C | -2.034864000 | -3.724879000 | -2.607093000 |
| C | -0.935787000 | -3.105199000 | -3.183112000 |
| C | -0.429519000 | -1.905146000 | -2.685312000 |
| C | -1.063163000 | -1.331135000 | -1.587311000 |
| C | -4.525250000 | 0.311842000 | -0.887380000 |
| C | -3.931112000 | 1.601951000 | -0.324393000 |
| C | -2.391077000 | 1.525053000 | -0.293985000 |
| C | -1.802907000 | 1.949395000 | -1.635165000 |
| C | -5.526445000 | 4.115549000 | 0.673451000 |
| C | -5.202560000 | 2.876967000 | 1.449495000 |
| C | -4.511841000 | 1.812463000 | 1.050640000 |
| C | -4.243696000 | 0.668760000 | 1.988179000 |
| N | 2.099453000 | -1.332409000 | -0.436655000 |
| C | 2.491569000 | 0.089592000 | -0.360623000 |
| C | 3.645206000 | 1.507469000 | 1.468661000 |
| N | 4.620216000 | 2.183065000 | 0.610137000 |
| C | 5.653958000 | 1.160062000 | 0.433984000 |
| C | 4.926571000 | -0.217382000 | 0.398989000 |
| C | 3.476911000 | 0.103304000 | 0.843913000 |

| | | | |
|---|---|---|---|
| C | 2.844997000 | -0.965133000 | 1.713392000 |
| C | 2.916374000 | -1.188380000 | 3.073500000 |
| C | 2.139454000 | -2.213745000 | 3.616880000 |
| C | 1.282328000 | -2.963723000 | 2.828324000 |
| C | 1.195380000 | -2.728677000 | 1.455604000 |
| C | 2.037839000 | -1.773000000 | 0.912974000 |
| C | 2.376546000 | 2.314886000 | 1.623852000 |
| C | 1.692314000 | 2.416866000 | 0.257607000 |
| C | 1.274238000 | 0.979271000 | -0.114998000 |
| C | 0.244626000 | 0.787214000 | -1.250761000 |
| C | 1.004344000 | 4.851355000 | -1.571314000 |
| C | 2.337359000 | 4.192352000 | -1.404829000 |
| C | 2.654550000 | 3.127465000 | -0.669980000 |
| C | 4.086563000 | 2.623444000 | -0.695795000 |
| C | 2.502388000 | -2.088255000 | -1.514822000 |
| C | 2.502334000 | -3.586206000 | -1.365424000 |
| H | -1.299582000 | 0.272807000 | 1.087349000 |
| H | -4.674982000 | -1.741216000 | -0.303194000 |
| H | -4.000116000 | -1.527034000 | 3.205726000 |
| H | -4.596987000 | -2.630488000 | 1.969010000 |
| H | -2.351325000 | -2.925554000 | 1.370247000 |
| H | -1.826298000 | -1.454283000 | 2.154179000 |
| H | -3.519475000 | -3.612046000 | -1.051094000 |
| H | -2.402149000 | -4.659261000 | -3.004153000 |
| H | -0.449135000 | -3.560221000 | -4.034576000 |
| H | 0.437550000 | -1.452162000 | -3.141637000 |
| H | -5.605082000 | 0.410853000 | -0.988637000 |
| H | -4.116362000 | 0.087909000 | -1.874592000 |
| H | -4.203499000 | 2.433553000 | -0.971239000 |
| H | -2.035586000 | 2.262348000 | 0.429346000 |
| H | -2.110925000 | 1.272170000 | -2.438798000 |
| H | -2.135780000 | 2.953308000 | -1.891779000 |
| H | -5.094378000 | 4.121899000 | -0.322672000 |
| H | -6.606284000 | 4.231697000 | 0.574187000 |
| H | -5.164481000 | 4.997766000 | 1.202284000 |
| H | -5.581939000 | 2.863660000 | 2.466661000 |
| H | -3.167455000 | 0.657547000 | 2.228610000 |
| H | -4.771295000 | 0.816048000 | 2.928974000 |
| H | 2.959119000 | 0.339215000 | -1.304140000 |
| H | 4.126293000 | 1.378370000 | 2.442363000 |
| H | 6.329157000 | 1.195281000 | 1.287926000 |
| H | 6.237827000 | 1.357574000 | -0.461571000 |
| H | 5.396481000 | -0.916887000 | 1.086961000 |
| H | 4.939153000 | -0.672755000 | -0.590887000 |

| | | | |
|---|---|---|---|
| H | 3.541693000 | -0.578253000 | 3.709932000 |
| H | 2.177558000 | -2.406904000 | 4.679172000 |
| H | 0.643881000 | -3.720389000 | 3.260929000 |
| H | 2.629635000 | 3.304799000 | 2.001990000 |
| H | 1.714985000 | 1.835959000 | 2.346761000 |
| H | 0.784674000 | 3.003905000 | 0.349703000 |
| H | 0.785090000 | 0.599667000 | 0.785881000 |
| H | 0.749123000 | 0.432893000 | -2.153524000 |
| H | 0.231298000 | 4.410060000 | -0.951822000 |
| H | 1.080586000 | 5.914901000 | -1.342645000 |
| H | 0.674611000 | 4.772611000 | -2.608460000 |
| H | 3.142405000 | 4.654365000 | -1.969127000 |
| H | 4.168634000 | 1.815567000 | -1.432041000 |
| H | 4.730824000 | 3.421663000 | -1.061992000 |
| H | 3.216192000 | -3.984765000 | -2.078473000 |
| H | 2.749974000 | -3.910738000 | -0.358933000 |
| H | 1.515343000 | -3.973452000 | -1.619782000 |
| O | 2.820106000 | -1.557852000 | -2.565123000 |
| O | -0.392809000 | 2.023057000 | -1.547495000 |
| O | 0.295768000 | -3.432141000 | 0.722198000 |
| H | 0.110461000 | -2.974770000 | -0.110166000 |

Compound: **10a**

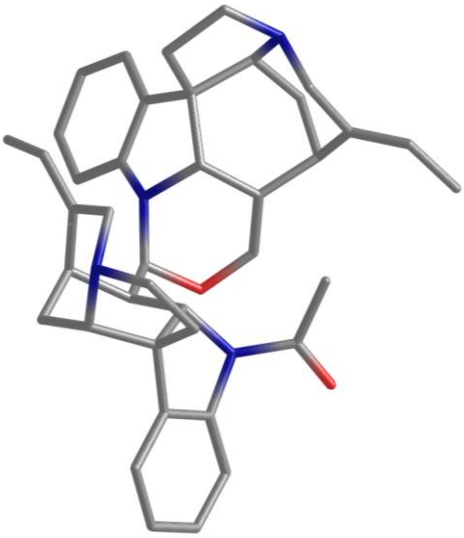

symmetry c1

| | | | |
|---|---|---|---|
| N | -1.165019000 | -1.076360000 | -0.850954000 |
| C | -1.642989000 | 0.039553000 | -0.022584000 |
| C | -4.080545000 | 0.891824000 | 0.368648000 |
| N | -3.958887000 | 1.412869000 | 1.730600000 |
| C | -4.010755000 | 0.193451000 | 2.540780000 |
| C | -3.289014000 | -0.920851000 | 1.727249000 |
| C | -3.126963000 | -0.329534000 | 0.302718000 |
| C | -3.444488000 | -1.285563000 | -0.819330000 |
| C | -4.658498000 | -1.817360000 | -1.202573000 |
| C | -4.706883000 | -2.713839000 | -2.270131000 |
| C | -3.538328000 | -3.060615000 | -2.932014000 |
| C | -2.305430000 | -2.538640000 | -2.548165000 |
| C | -2.271541000 | -1.653800000 | -1.480184000 |
| C | -3.854550000 | 1.944042000 | -0.693872000 |
| C | -2.424194000 | 2.481667000 | -0.541353000 |
| C | -1.493648000 | 1.319308000 | -0.811458000 |
| C | -0.671423000 | 1.339509000 | -1.858247000 |
| C | -1.672541000 | 5.441737000 | 0.151264000 |
| C | -2.080091000 | 4.364341000 | 1.107150000 |
| C | -2.351656000 | 3.088116000 | 0.845689000 |
| C | -2.704158000 | 2.145585000 | 1.967504000 |
| N | 2.401898000 | 1.174877000 | -0.650755000 |
| C | 1.913155000 | 0.057461000 | 0.181418000 |
| C | 3.239578000 | -1.857248000 | 1.401794000 |
| N | 2.525652000 | -1.830430000 | 2.678883000 |
| C | 3.105031000 | -0.657848000 | 3.341925000 |
| C | 3.441565000 | 0.376909000 | 2.225306000 |

| | | | |
|---|---|---|---|
| C | 3.228330000 | -0.400320000 | 0.901772000 |
| C | 4.245860000 | -0.089444000 | -0.170883000 |
| C | 5.532334000 | -0.560021000 | -0.342314000 |
| C | 6.282780000 | -0.094108000 | -1.421498000 |
| C | 5.743362000 | 0.829574000 | -2.304167000 |
| C | 4.446476000 | 1.313889000 | -2.140700000 |
| C | 3.717889000 | 0.839365000 | -1.066193000 |
| C | 2.680583000 | -2.866029000 | 0.427912000 |
| C | 1.257699000 | -2.433631000 | 0.077285000 |
| C | 1.349360000 | -1.087745000 | -0.689246000 |
| C | 0.071720000 | -0.897202000 | -1.535719000 |
| C | -1.384490000 | -4.028349000 | 0.610513000 |
| C | -0.598522000 | -3.221414000 | 1.592378000 |
| C | 0.481518000 | -2.471486000 | 1.380760000 |
| C | 1.065665000 | -1.693774000 | 2.538752000 |
| C | 1.991918000 | 2.485032000 | -0.636886000 |
| C | 1.000561000 | 2.951491000 | 0.400123000 |
| H | -1.055599000 | 0.064517000 | 0.898137000 |
| H | -5.096576000 | 0.498711000 | 0.280049000 |
| H | -3.564780000 | 0.366857000 | 3.517511000 |
| H | -5.054578000 | -0.077670000 | 2.693012000 |
| H | -3.890381000 | -1.827598000 | 1.702710000 |
| H | -2.320546000 | -1.185167000 | 2.147900000 |
| H | -5.567154000 | -1.545822000 | -0.680647000 |
| H | -5.651537000 | -3.132976000 | -2.583258000 |
| H | -3.578728000 | -3.750586000 | -3.762973000 |
| H | -1.408680000 | -2.835498000 | -3.072303000 |
| H | -4.579615000 | 2.747956000 | -0.571881000 |
| H | -3.986724000 | 1.501931000 | -1.681633000 |
| H | -2.238986000 | 3.244936000 | -1.292723000 |
| H | -0.565015000 | 2.208583000 | -2.495091000 |
| H | -0.734040000 | 5.894844000 | 0.473918000 |
| H | -1.537491000 | 5.086574000 | -0.866474000 |
| H | -2.415528000 | 6.239837000 | 0.134640000 |
| H | -2.147778000 | 4.678787000 | 2.144099000 |
| H | -1.866485000 | 1.448245000 | 2.123305000 |
| H | -2.810308000 | 2.703571000 | 2.896548000 |
| H | 1.180038000 | 0.428068000 | 0.882222000 |
| H | 4.277772000 | -2.106019000 | 1.637880000 |
| H | 2.420948000 | -0.270232000 | 4.093014000 |
| H | 4.020324000 | -0.959421000 | 3.848795000 |
| H | 4.473188000 | 0.710520000 | 2.311787000 |
| H | 2.810661000 | 1.264128000 | 2.267845000 |
| H | 5.959654000 | -1.274917000 | 0.347927000 |

| | | | |
|---|---|---|---|
| H | 7.288880000 | -0.457748000 | -1.571722000 |
| H | 6.334152000 | 1.180171000 | -3.138217000 |
| H | 4.022894000 | 2.033335000 | -2.820430000 |
| H | 2.680783000 | -3.854526000 | 0.885071000 |
| H | 3.305383000 | -2.902022000 | -0.465061000 |
| H | 0.822402000 | -3.155682000 | -0.610615000 |
| H | 2.120418000 | -1.246379000 | -1.449293000 |
| H | 0.126911000 | -1.681760000 | -2.286977000 |
| H | -2.406066000 | -3.646370000 | 0.544654000 |
| H | -0.971391000 | -4.009472000 | -0.393057000 |
| H | -1.452951000 | -5.065767000 | 0.939524000 |
| H | -0.983807000 | -3.248741000 | 2.607493000 |
| H | 0.784210000 | -0.635137000 | 2.442771000 |
| H | 0.605477000 | -2.035366000 | 3.465099000 |
| H | 1.579244000 | 3.464851000 | 1.169827000 |
| H | 0.395165000 | 2.185210000 | 0.866607000 |
| H | 0.343888000 | 3.678645000 | -0.065402000 |
| O | 2.486972000 | 3.299572000 | -1.394679000 |
| O | 0.112876000 | 0.320889000 | -2.291514000 |

Compound: **10b**

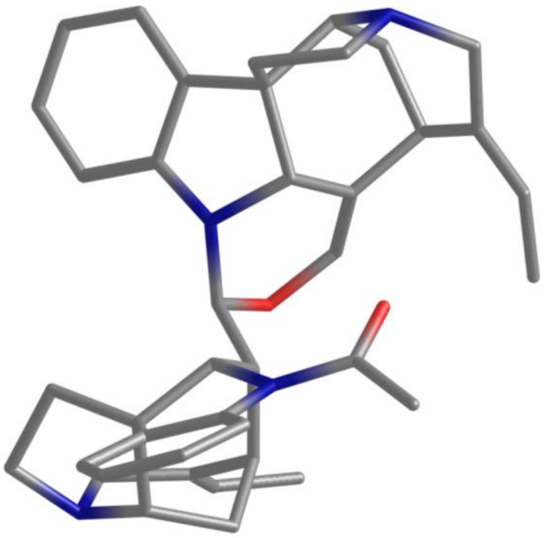

symmetry c1

| | | | |
|---|---|---|---|
| N | 0.943178000 | -0.946340000 | -0.609434000 |
| C | 1.904930000 | 0.104774000 | -0.224140000 |
| C | 4.362312000 | 0.525764000 | -0.969575000 |
| N | 4.344040000 | 2.006935000 | -0.980740000 |
| C | 3.127147000 | 2.454574000 | -1.675512000 |
| C | 2.599555000 | 1.211239000 | -2.385803000 |
| C | 2.941376000 | 0.099746000 | -1.383223000 |
| C | 2.797654000 | -1.298881000 | -1.919859000 |
| C | 3.592860000 | -2.003754000 | -2.800087000 |
| C | 3.218431000 | -3.293905000 | -3.176799000 |
| C | 2.055295000 | -3.852491000 | -2.668608000 |
| C | 1.239880000 | -3.148538000 | -1.785400000 |
| C | 1.621681000 | -1.865646000 | -1.422366000 |
| C | 4.814950000 | -0.026392000 | 0.373060000 |
| C | 3.816581000 | 0.423115000 | 1.447827000 |
| C | 2.506464000 | -0.236316000 | 1.110983000 |
| C | 1.952607000 | -1.170704000 | 1.873990000 |
| C | 2.193598000 | 2.093524000 | 3.434038000 |
| C | 2.990723000 | 2.646547000 | 2.294427000 |
| C | 3.721112000 | 1.947692000 | 1.432033000 |
| C | 4.525959000 | 2.614252000 | 0.339152000 |
| N | -1.962944000 | 1.813485000 | 0.069726000 |
| C | -1.622203000 | 0.384209000 | -0.233387000 |
| C | -3.838688000 | -0.802859000 | 0.477818000 |
| N | -3.909249000 | -2.247597000 | 0.255951000 |
| C | -3.958612000 | -2.346630000 | -1.203345000 |
| C | -2.891433000 | -1.355450000 | -1.702720000 |

| | | | |
|---|---|---|---|
| C | -2.985628000 | -0.192772000 | -0.700474000 |
| C | -3.679253000 | 1.031100000 | -1.224028000 |
| C | -4.772886000 | 1.116831000 | -2.062638000 |
| C | -5.276110000 | 2.368612000 | -2.400747000 |
| C | -4.666635000 | 3.510403000 | -1.903355000 |
| C | -3.561719000 | 3.434596000 | -1.061062000 |
| C | -3.087682000 | 2.177539000 | -0.704776000 |
| C | -3.309605000 | -0.415408000 | 1.840484000 |
| C | -1.897142000 | -0.982956000 | 1.992938000 |
| C | -0.930762000 | -0.364885000 | 0.927949000 |
| C | 0.086448000 | -1.414370000 | 0.434807000 |
| C | -0.754717000 | -2.977389000 | 4.089338000 |
| C | -1.625020000 | -3.306249000 | 2.918532000 |
| C | -2.065030000 | -2.486181000 | 1.967551000 |
| C | -2.805493000 | -3.063044000 | 0.785445000 |
| C | -1.140210000 | 2.678917000 | 0.739129000 |
| C | -1.733187000 | 3.953158000 | 1.291036000 |
| H | 1.395218000 | 1.065536000 | -0.160197000 |
| H | 5.044344000 | 0.175048000 | -1.749361000 |
| H | 2.383773000 | 2.822663000 | -0.958547000 |
| H | 3.349893000 | 3.265945000 | -2.368057000 |
| H | 3.137669000 | 1.035836000 | -3.318128000 |
| H | 1.534000000 | 1.263828000 | -2.605171000 |
| H | 4.499839000 | -1.564534000 | -3.194837000 |
| H | 3.836150000 | -3.859589000 | -3.858637000 |
| H | 1.773272000 | -4.855398000 | -2.956828000 |
| H | 0.339943000 | -3.606880000 | -1.400257000 |
| H | 5.822989000 | 0.322061000 | 0.596605000 |
| H | 4.846162000 | -1.114605000 | 0.324892000 |
| H | 4.144272000 | 0.078899000 | 2.430843000 |
| H | 2.373314000 | -1.481179000 | 2.822259000 |
| H | 2.503415000 | 1.086202000 | 3.701924000 |
| H | 2.290007000 | 2.729191000 | 4.313789000 |
| H | 1.135930000 | 2.058975000 | 3.164832000 |
| H | 2.956171000 | 3.724364000 | 2.167771000 |
| H | 4.267579000 | 3.669840000 | 0.269313000 |
| H | 5.587379000 | 2.572349000 | 0.595114000 |
| H | -0.940695000 | 0.392556000 | -1.086842000 |
| H | -4.856097000 | -0.427125000 | 0.356869000 |
| H | -3.792890000 | -3.370439000 | -1.530810000 |
| H | -4.948320000 | -2.039047000 | -1.545090000 |
| H | -3.052506000 | -1.029103000 | -2.727973000 |
| H | -1.899824000 | -1.810043000 | -1.663392000 |
| H | -5.225896000 | 0.218538000 | -2.461615000 |

| | | | |
|---|---|---|---|
| H | -6.126881000 | 2.451422000 | -3.060837000 |
| H | -5.039911000 | 4.484458000 | -2.185091000 |
| H | -3.090254000 | 4.344476000 | -0.733369000 |
| H | -3.953542000 | -0.824727000 | 2.618315000 |
| H | -3.320257000 | 0.669908000 | 1.939853000 |
| H | -1.514695000 | -0.705108000 | 2.969351000 |
| H | -0.326604000 | 0.373267000 | 1.448622000 |
| H | -0.420394000 | -2.300584000 | 0.072583000 |
| H | -0.417604000 | -1.945428000 | 4.088747000 |
| H | -1.270276000 | -3.178487000 | 5.029469000 |
| H | 0.133485000 | -3.610345000 | 4.072485000 |
| H | -1.893596000 | -4.355000000 | 2.828525000 |
| H | -2.077016000 | -3.256674000 | -0.013266000 |
| H | -3.202223000 | -4.041157000 | 1.058539000 |
| H | -2.809675000 | 3.905890000 | 1.419507000 |
| H | -1.250933000 | 4.123059000 | 2.249634000 |
| H | -1.482208000 | 4.792741000 | 0.644978000 |
| O | 0.030572000 | 2.417703000 | 0.956624000 |
| O | 0.825311000 | -1.876157000 | 1.577022000 |

Compound: **11-*tub***

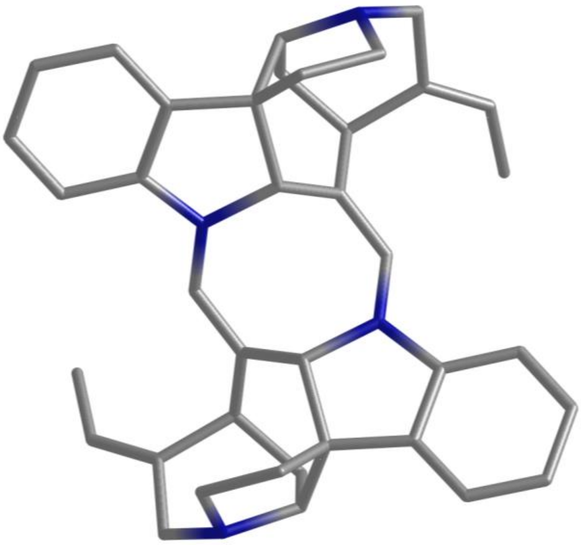

symmetry c2

| | | | |
|---|---|---|---|
| N | 1.328419000 | 1.098337000 | -0.225435000 |
| N | -1.950942000 | 4.159464000 | -0.370753000 |
| N | -1.328419000 | -1.098337000 | -0.225435000 |
| N | 1.950942000 | -4.159464000 | -0.370753000 |
| C | -0.062789000 | 1.496756000 | -0.558365000 |
| C | 0.062789000 | 3.002528000 | -0.973624000 |
| C | -1.063084000 | 1.183100000 | 0.530771000 |
| C | 1.676137000 | 0.003999000 | 0.545875000 |
| C | 0.062789000 | -1.496756000 | -0.558365000 |
| C | -0.062789000 | -3.002528000 | -0.973624000 |
| C | 1.063084000 | -1.183100000 | 0.530771000 |
| C | -1.676137000 | -0.003999000 | 0.545875000 |
| C | -0.552290000 | 3.968611000 | 0.064699000 |
| C | -1.390370000 | 2.219148000 | 1.603385000 |
| C | -0.441395000 | 3.410740000 | 1.476538000 |
| C | -0.794746000 | 3.299194000 | -2.206183000 |
| C | 0.552290000 | -3.968611000 | 0.064699000 |
| C | 1.390370000 | -2.219148000 | 1.603385000 |
| C | 0.441395000 | -3.410740000 | 1.476538000 |
| C | 0.794746000 | -3.299194000 | -2.206183000 |
| C | 1.549137000 | 3.196852000 | -1.104222000 |
| C | 2.223389000 | 2.078649000 | -0.612117000 |
| C | -2.186792000 | 3.401917000 | -1.599284000 |
| C | -2.818602000 | 2.727012000 | 1.487760000 |
| C | -2.947915000 | 4.003523000 | 0.671850000 |
| C | -1.549137000 | -3.196852000 | -1.104222000 |
| C | -2.223389000 | -2.078649000 | -0.612117000 |

| | | | |
|---|---|---|---|
| C | 2.186792000 | -3.401917000 | -1.599284000 |
| C | 2.818602000 | -2.727012000 | 1.487760000 |
| C | 2.947915000 | -4.003523000 | 0.671850000 |
| C | 2.258192000 | 4.291944000 | -1.549622000 |
| C | 3.610991000 | 2.034412000 | -0.566539000 |
| C | -3.876701000 | 2.190879000 | 2.088470000 |
| C | 3.653563000 | 4.267768000 | -1.508275000 |
| C | 4.312926000 | 3.149733000 | -1.016981000 |
| C | -3.913354000 | 1.027994000 | 3.033291000 |
| C | -2.258192000 | -4.291944000 | -1.549622000 |
| C | -3.610991000 | -2.034412000 | -0.566539000 |
| C | 3.876701000 | -2.190879000 | 2.088470000 |
| C | -3.653563000 | -4.267768000 | -1.508275000 |
| C | -4.312926000 | -3.149733000 | -1.016981000 |
| C | 3.913354000 | -1.027994000 | 3.033291000 |
| H | -0.383766000 | 0.951190000 | -1.447269000 |
| H | -0.013359000 | 4.917392000 | -0.000195000 |
| H | -1.252251000 | 1.759172000 | 2.580157000 |
| H | -0.695874000 | 4.168486000 | 2.216489000 |
| H | 0.592119000 | 3.112454000 | 1.662131000 |
| H | -0.706591000 | 2.536592000 | -2.976407000 |
| H | 0.383766000 | -0.951190000 | -1.447269000 |
| H | 0.013359000 | -4.917392000 | -0.000195000 |
| H | 1.252251000 | -1.759172000 | 2.580157000 |
| H | 0.695874000 | -4.168486000 | 2.216489000 |
| H | -0.592119000 | -3.112454000 | 1.662131000 |
| H | 0.706591000 | -2.536592000 | -2.976407000 |
| H | -0.503013000 | 4.260142000 | -2.631433000 |
| H | -2.894648000 | 3.922501000 | -2.243499000 |
| H | -2.588116000 | 2.398967000 | -1.396309000 |
| H | -2.860196000 | 4.854167000 | 1.353303000 |
| H | -3.938521000 | 4.070245000 | 0.223234000 |
| H | 1.738893000 | 5.164716000 | -1.924831000 |
| H | 0.503013000 | -4.260142000 | -2.631433000 |
| H | 2.894648000 | -3.922501000 | -2.243499000 |
| H | 2.588116000 | -2.398967000 | -1.396309000 |
| H | 2.860196000 | -4.854167000 | 1.353303000 |
| H | 3.938521000 | -4.070245000 | 0.223234000 |
| H | -1.738893000 | -5.164716000 | -1.924831000 |
| H | 4.131111000 | 1.154737000 | -0.214603000 |
| H | -4.839127000 | 2.658494000 | 1.899061000 |
| H | 4.219492000 | 5.116921000 | -1.861469000 |
| H | 5.393512000 | 3.134950000 | -0.993499000 |
| H | -2.920480000 | 0.673096000 | 3.300148000 |

| | | | |
|---|---|---|---|
| H | -4.432438000 | 1.302666000 | 3.951813000 |
| H | -4.464090000 | 0.189266000 | 2.602956000 |
| H | -4.131111000 | -1.154737000 | -0.214603000 |
| H | 4.839127000 | -2.658494000 | 1.899061000 |
| H | -4.219492000 | -5.116921000 | -1.861469000 |
| H | -5.393512000 | -3.134950000 | -0.993499000 |
| H | 2.920480000 | -0.673096000 | 3.300148000 |
| H | 4.432438000 | -1.302666000 | 3.951813000 |
| H | 4.464090000 | -0.189266000 | 2.602956000 |
| H | 2.499887000 | 0.184760000 | 1.228969000 |
| H | -2.499887000 | -0.184760000 | 1.228969000 |

Compound: **11-*crown***

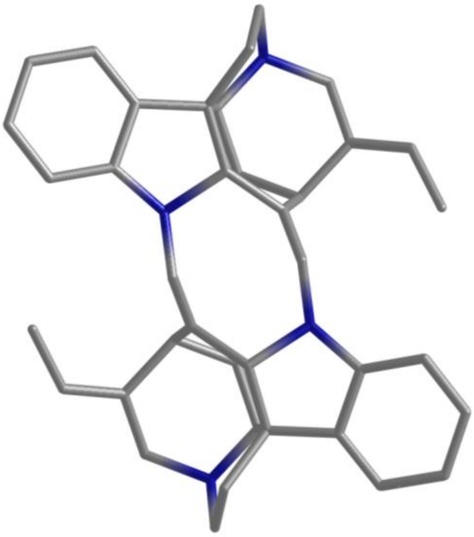

symmetry c2

| | | | |
|---|---|---|---|
| N | -1.043969000 | -1.572792000 | -0.693162000 |
| N | 1.892376000 | -3.918359000 | 1.633949000 |
| C | 0.421170000 | -1.948270000 | -0.925959000 |
| C | 0.477478000 | -3.328152000 | -0.278930000 |
| C | 1.262445000 | -0.828133000 | -0.381924000 |
| C | -1.305254000 | -0.260669000 | -1.166480000 |
| C | 0.634481000 | -3.236809000 | 1.297452000 |
| C | 1.663584000 | -0.962445000 | 1.067781000 |
| C | 0.634481000 | -1.806554000 | 1.837021000 |
| C | 1.679533000 | -4.165676000 | -0.729936000 |
| C | -0.886644000 | -3.849708000 | -0.681271000 |
| C | -1.733497000 | -2.760619000 | -0.945656000 |
| C | 2.119133000 | -4.872347000 | 0.554001000 |
| C | 3.014195000 | -1.692482000 | 1.145449000 |
| C | 3.001644000 | -3.001489000 | 1.907046000 |
| C | -1.352140000 | -5.138687000 | -0.807896000 |
| C | -3.033741000 | -2.959870000 | -1.377741000 |
| C | 4.158754000 | -1.194031000 | 0.687704000 |
| C | -2.670939000 | -5.352482000 | -1.219846000 |
| C | -3.490307000 | -4.272583000 | -1.505881000 |
| C | 4.356703000 | 0.152930000 | 0.072922000 |
| H | 0.563781000 | -2.045682000 | -2.010224000 |
| H | -1.124155000 | -0.116135000 | -2.231977000 |
| H | -0.190339000 | -3.796242000 | 1.740927000 |
| H | 1.779685000 | 0.026039000 | 1.510355000 |
| H | 0.886339000 | -1.826818000 | 2.896859000 |
| H | -0.358242000 | -1.383699000 | 1.744365000 |

| | | | |
|---|---|---|---|
| H | 2.475768000 | -3.504125000 | -1.077110000 |
| H | 1.427510000 | -4.848326000 | -1.538962000 |
| H | 1.488551000 | -5.747748000 | 0.728186000 |
| H | 3.154060000 | -5.205501000 | 0.541237000 |
| H | 2.972553000 | -2.755480000 | 2.972209000 |
| H | 3.937828000 | -3.531633000 | 1.743551000 |
| H | -0.700916000 | -5.979887000 | -0.606396000 |
| H | -3.674599000 | -2.126089000 | -1.619913000 |
| H | 5.047832000 | -1.807956000 | 0.791640000 |
| H | -3.045626000 | -6.359440000 | -1.329862000 |
| H | -4.502479000 | -4.444755000 | -1.844074000 |
| H | 3.561906000 | 0.844452000 | 0.342303000 |
| H | 5.305805000 | 0.582176000 | 0.391395000 |
| H | 4.380525000 | 0.085567000 | -1.017149000 |
| N | 1.043969000 | 1.572792000 | -0.693162000 |
| N | -1.892376000 | 3.918359000 | 1.633949000 |
| C | -0.421170000 | 1.948270000 | -0.925959000 |
| C | -0.477478000 | 3.328152000 | -0.278930000 |
| C | -1.262445000 | 0.828133000 | -0.381924000 |
| C | 1.305254000 | 0.260669000 | -1.166480000 |
| C | -0.634481000 | 3.236809000 | 1.297452000 |
| C | -1.663584000 | 0.962445000 | 1.067781000 |
| C | -0.634481000 | 1.806554000 | 1.837021000 |
| C | -1.679533000 | 4.165676000 | -0.729936000 |
| C | 0.886644000 | 3.849708000 | -0.681271000 |
| C | 1.733497000 | 2.760619000 | -0.945656000 |
| C | -2.119133000 | 4.872347000 | 0.554001000 |
| C | -3.014195000 | 1.692482000 | 1.145449000 |
| C | -3.001644000 | 3.001489000 | 1.907046000 |
| C | 1.352140000 | 5.138687000 | -0.807896000 |
| C | 3.033741000 | 2.959870000 | -1.377741000 |
| C | -4.158754000 | 1.194031000 | 0.687704000 |
| C | 2.670939000 | 5.352482000 | -1.219846000 |
| C | 3.490307000 | 4.272583000 | -1.505881000 |
| C | -4.356703000 | -0.152930000 | 0.072922000 |
| H | -0.563781000 | 2.045682000 | -2.010224000 |
| H | 1.124155000 | 0.116135000 | -2.231977000 |
| H | 0.190339000 | 3.796242000 | 1.740927000 |
| H | -1.779685000 | -0.026039000 | 1.510355000 |
| H | -0.886339000 | 1.826818000 | 2.896859000 |
| H | 0.358242000 | 1.383699000 | 1.744365000 |
| H | -2.475768000 | 3.504125000 | -1.077110000 |
| H | -1.427510000 | 4.848326000 | -1.538962000 |
| H | -1.488551000 | 5.747748000 | 0.728186000 |

| | | | |
|---|---|---|---|
| H | -3.154060000 | 5.205501000 | 0.541237000 |
| H | -2.972553000 | 2.755480000 | 2.972209000 |
| H | -3.937828000 | 3.531633000 | 1.743551000 |
| H | 0.700916000 | 5.979887000 | -0.606396000 |
| H | 3.674599000 | 2.126089000 | -1.619913000 |
| H | -5.047832000 | 1.807956000 | 0.791640000 |
| H | 3.045626000 | 6.359440000 | -1.329862000 |
| H | 4.502479000 | 4.444755000 | -1.844074000 |
| H | -3.561906000 | -0.844452000 | 0.342303000 |
| H | -5.305805000 | -0.582176000 | 0.391395000 |
| H | -4.380525000 | -0.085567000 | -1.017149000 |

Compound: **12-*boat-like***

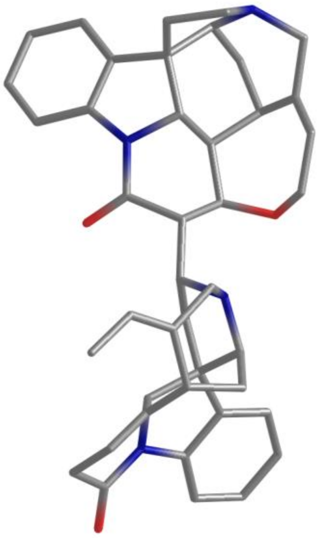

symmetry c1

| | | | |
|---|---|---|---|
| N | 2.644005000 | 1.381567000 | -0.271495000 |
| C | 3.356385000 | 0.157844000 | 0.137847000 |
| C | 5.706150000 | -0.773665000 | -0.369254000 |
| N | 6.002466000 | -1.372859000 | 0.956989000 |
| C | 5.703959000 | -0.379622000 | 1.999714000 |
| C | 5.494333000 | 0.933869000 | 1.254430000 |
| C | 4.850563000 | 0.477887000 | -0.065867000 |
| C | 4.783961000 | 1.588893000 | -1.080984000 |
| C | 5.785346000 | 2.142594000 | -1.853541000 |
| C | 5.491593000 | 3.239506000 | -2.661090000 |
| C | 4.211026000 | 3.773515000 | -2.674661000 |
| C | 3.189821000 | 3.232036000 | -1.897427000 |
| C | 3.495569000 | 2.132380000 | -1.111966000 |
| C | 5.045966000 | -1.797758000 | -1.279537000 |
| C | 3.696781000 | -2.197390000 | -0.670760000 |
| C | 2.817111000 | -0.954923000 | -0.756889000 |
| C | 1.313157000 | -1.177097000 | -0.490827000 |
| C | 1.431730000 | -2.648159000 | 1.436713000 |
| C | 2.910844000 | -2.789758000 | 1.655199000 |
| C | 3.896122000 | -2.626792000 | 0.778848000 |
| C | 5.347328000 | -2.668829000 | 1.193528000 |
| C | 1.277317000 | 1.311114000 | -0.375193000 |
| C | 0.704925000 | 0.042139000 | 0.252213000 |
| N | -4.903391000 | 0.475174000 | -1.131392000 |
| C | -3.784537000 | 0.647158000 | -0.175047000 |
| C | -2.629442000 | -1.307522000 | 1.117497000 |
| N | -1.314722000 | -0.722954000 | 1.476026000 |

| | | | |
|---|---|---|---|
| C | -0.821757000 | 0.076822000 | 0.342826000 |
| C | -1.593682000 | -0.475914000 | -0.843834000 |
| C | -2.992041000 | -0.693855000 | -0.253374000 |
| C | -3.859466000 | -1.557728000 | -1.127040000 |
| C | -3.686482000 | -2.883385000 | -1.475104000 |
| C | -4.589233000 | -3.477109000 | -2.353040000 |
| C | -5.641728000 | -2.736693000 | -2.873719000 |
| C | -5.826161000 | -1.398978000 | -2.537279000 |
| C | -4.921378000 | -0.828602000 | -1.652438000 |
| C | -3.689524000 | -1.060140000 | 2.182273000 |
| C | -3.809925000 | 0.447240000 | 2.429856000 |
| C | -4.342028000 | 1.080866000 | 1.169724000 |
| C | -5.305884000 | 1.990842000 | 1.124494000 |
| C | -3.304966000 | 3.296393000 | 3.472330000 |
| C | -2.253032000 | 2.251150000 | 3.261362000 |
| C | -2.448846000 | 1.006062000 | 2.838141000 |
| C | -1.317294000 | 0.013125000 | 2.735926000 |
| C | -5.921965000 | 1.375553000 | -1.195081000 |
| C | -5.773433000 | 2.514483000 | -0.207181000 |
| H | 3.156498000 | -0.057909000 | 1.184507000 |
| H | 6.641138000 | -0.430292000 | -0.818833000 |
| H | 6.513680000 | -0.318970000 | 2.725683000 |
| H | 4.793744000 | -0.654106000 | 2.544545000 |
| H | 4.873712000 | 1.643482000 | 1.799005000 |
| H | 6.448341000 | 1.412706000 | 1.033237000 |
| H | 6.786561000 | 1.733351000 | -1.830282000 |
| H | 6.264792000 | 3.678488000 | -3.274379000 |
| H | 3.995401000 | 4.627399000 | -3.300836000 |
| H | 2.194772000 | 3.643377000 | -1.907408000 |
| H | 5.705468000 | -2.655360000 | -1.399402000 |
| H | 4.897643000 | -1.370365000 | -2.270723000 |
| H | 3.245685000 | -3.006100000 | -1.245141000 |
| H | 2.897071000 | -0.573415000 | -1.778793000 |
| H | 0.825634000 | -1.247645000 | -1.460597000 |
| H | 0.937799000 | -3.574442000 | 1.734036000 |
| H | 1.030681000 | -1.876097000 | 2.102397000 |
| H | 3.170286000 | -3.047860000 | 2.678006000 |
| H | 5.882509000 | -3.443182000 | 0.643243000 |
| H | 5.443149000 | -2.907438000 | 2.250718000 |
| H | 1.082220000 | 0.027067000 | 1.276576000 |
| H | -3.144976000 | 1.454195000 | -0.546167000 |
| H | -2.509973000 | -2.383365000 | 0.970644000 |
| H | -1.104139000 | 1.129975000 | 0.475530000 |
| H | -1.588833000 | 0.186885000 | -1.707265000 |

| | | | |
|---|---|---|---|
| H | -1.199924000 | -1.447910000 | -1.139508000 |
| H | -2.856306000 | -3.452111000 | -1.076743000 |
| H | -4.466882000 | -4.512960000 | -2.633410000 |
| H | -6.335628000 | -3.203594000 | -3.558122000 |
| H | -6.640168000 | -0.822314000 | -2.940684000 |
| H | -3.431708000 | -1.583512000 | 3.101505000 |
| H | -4.642013000 | -1.464295000 | 1.837034000 |
| H | -4.530192000 | 0.641552000 | 3.225823000 |
| H | -5.781202000 | 2.358827000 | 2.023140000 |
| H | -3.438186000 | 3.911629000 | 2.580357000 |
| H | -4.271302000 | 2.856511000 | 3.709865000 |
| H | -3.023184000 | 3.960245000 | 4.287040000 |
| H | -1.234209000 | 2.543826000 | 3.494354000 |
| H | -0.360632000 | 0.523094000 | 2.851450000 |
| H | -1.383025000 | -0.705931000 | 3.555515000 |
| H | -5.041396000 | 3.221334000 | -0.613108000 |
| H | -6.727882000 | 3.025725000 | -0.147834000 |
| O | 0.600665000 | 2.160112000 | -0.920469000 |
| O | 1.016884000 | -2.420526000 | 0.107600000 |
| O | -6.874628000 | 1.266376000 | -1.944205000 |

Compound: **12-*chair-like***

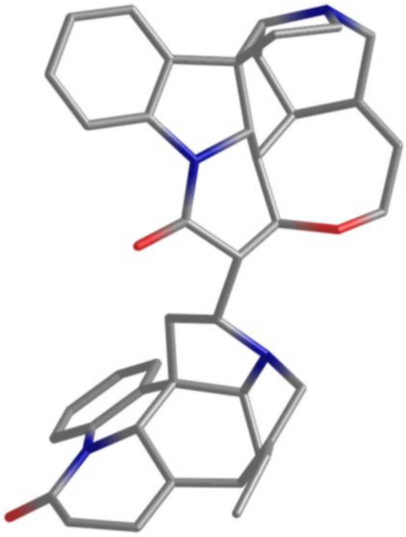

symmetry c1

| | | | |
|---|---|---|---|
| N | -2.496496000 | 0.770115000 | -1.351853000 |
| C | -3.335378000 | -0.109307000 | -0.510769000 |
| C | -5.454343000 | 0.459953000 | 0.884832000 |
| N | -6.204746000 | -0.819913000 | 0.811757000 |
| C | -6.150372000 | -1.322856000 | -0.568332000 |
| C | -5.718839000 | -0.123979000 | -1.398085000 |
| C | -4.720989000 | 0.587619000 | -0.468652000 |
| C | -4.402176000 | 1.978274000 | -0.942475000 |
| C | -5.204584000 | 3.102700000 | -0.947865000 |
| C | -4.727486000 | 4.274670000 | -1.528485000 |
| C | -3.466071000 | 4.300083000 | -2.106059000 |
| C | -2.647328000 | 3.174132000 | -2.115129000 |
| C | -3.131809000 | 2.019836000 | -1.516025000 |
| C | -4.531938000 | 0.497869000 | 2.094987000 |
| C | -3.521686000 | -0.647522000 | 1.973601000 |
| C | -2.592923000 | -0.271015000 | 0.813960000 |
| C | -1.374206000 | -1.152578000 | 0.541120000 |
| C | -2.165313000 | -3.222286000 | 1.444772000 |
| C | -3.658582000 | -3.115845000 | 1.585699000 |
| C | -4.274078000 | -1.959066000 | 1.796644000 |
| C | -5.774383000 | -1.818423000 | 1.793961000 |
| C | -1.142176000 | 0.584769000 | -1.393105000 |
| C | -0.627569000 | -0.690870000 | -0.730212000 |
| N | 4.687639000 | 1.398184000 | 0.178606000 |
| C | 3.764294000 | 0.392531000 | -0.396778000 |
| C | 2.552338000 | -1.451013000 | 0.988818000 |
| N | 1.421976000 | -1.810678000 | 0.100967000 |

| | | | |
|---|---|---|---|
| C | 0.890086000 | -0.598471000 | -0.527087000 |
| C | 1.355706000 | 0.513451000 | 0.402102000 |
| C | 2.775714000 | 0.058739000 | 0.763681000 |
| C | 3.353560000 | 0.802250000 | 1.935871000 |
| C | 2.928816000 | 0.809608000 | 3.250319000 |
| C | 3.584461000 | 1.622108000 | 4.171241000 |
| C | 4.646452000 | 2.417640000 | 3.763519000 |
| C | 5.084356000 | 2.424396000 | 2.442645000 |
| C | 4.423981000 | 1.599631000 | 1.542451000 |
| C | 3.794098000 | -2.290633000 | 0.720715000 |
| C | 4.169692000 | -2.167476000 | -0.759257000 |
| C | 4.576364000 | -0.736555000 | -1.009224000 |
| C | 5.641267000 | -0.360016000 | -1.704690000 |
| C | 4.275897000 | -2.608991000 | -3.797738000 |
| C | 3.057841000 | -2.773964000 | -2.941369000 |
| C | 2.991422000 | -2.614011000 | -1.623206000 |
| C | 1.721363000 | -2.874026000 | -0.850077000 |
| C | 5.804059000 | 1.793865000 | -0.491160000 |
| C | 5.968081000 | 1.104192000 | -1.830383000 |
| H | -3.422360000 | -1.089966000 | -0.981371000 |
| H | -6.168552000 | 1.283842000 | 0.948892000 |
| H | -7.116776000 | -1.719829000 | -0.875906000 |
| H | -5.413946000 | -2.130229000 | -0.661285000 |
| H | -5.277704000 | -0.395048000 | -2.355532000 |
| H | -6.562928000 | 0.540787000 | -1.583239000 |
| H | -6.194810000 | 3.072810000 | -0.513025000 |
| H | -5.343223000 | 5.162061000 | -1.536277000 |
| H | -3.105591000 | 5.211198000 | -2.561846000 |
| H | -1.670683000 | 3.194720000 | -2.565554000 |
| H | -5.117378000 | 0.418151000 | 3.008947000 |
| H | -4.017437000 | 1.458134000 | 2.129628000 |
| H | -2.917566000 | -0.711384000 | 2.883023000 |
| H | -2.188179000 | 0.714141000 | 1.072340000 |
| H | -0.692355000 | -1.102145000 | 1.395577000 |
| H | -1.658609000 | -2.853810000 | 2.344243000 |
| H | -1.868842000 | -4.258239000 | 1.301021000 |
| H | -4.245470000 | -4.017987000 | 1.459320000 |
| H | -6.134878000 | -1.527439000 | 2.781334000 |
| H | -6.248374000 | -2.767234000 | 1.550152000 |
| H | -0.821214000 | -1.479281000 | -1.464085000 |
| H | 3.204861000 | 0.874390000 | -1.204559000 |
| H | 2.247502000 | -1.585777000 | 2.029713000 |
| H | 1.341056000 | -0.438302000 | -1.514607000 |
| H | 1.328847000 | 1.494762000 | -0.062774000 |

| | | | |
|---|---|---|---|
| H | 0.747822000 | 0.540643000 | 1.309329000 |
| H | 2.093656000 | 0.195671000 | 3.561494000 |
| H | 3.263222000 | 1.636990000 | 5.202332000 |
| H | 5.147416000 | 3.049195000 | 4.483244000 |
| H | 5.906655000 | 3.040774000 | 2.123555000 |
| H | 3.612443000 | -3.330187000 | 0.988108000 |
| H | 4.609746000 | -1.932871000 | 1.350548000 |
| H | 5.032883000 | -2.796460000 | -0.981134000 |
| H | 6.299099000 | -1.080689000 | -2.170190000 |
| H | 4.352830000 | -1.592332000 | -4.188181000 |
| H | 5.190464000 | -2.813208000 | -3.244687000 |
| H | 4.236043000 | -3.283457000 | -4.650575000 |
| H | 2.147364000 | -3.067169000 | -3.454139000 |
| H | 0.875752000 | -2.989843000 | -1.526749000 |
| H | 1.806170000 | -3.814745000 | -0.302231000 |
| H | 5.286869000 | 1.583891000 | -2.541871000 |
| H | 6.981647000 | 1.281976000 | -2.171982000 |
| O | -0.393532000 | 1.368128000 | -1.948145000 |
| O | -1.690539000 | -2.513201000 | 0.311361000 |
| O | 6.607846000 | 2.595454000 | -0.052714000 |

Compound: **13-*boat-like***

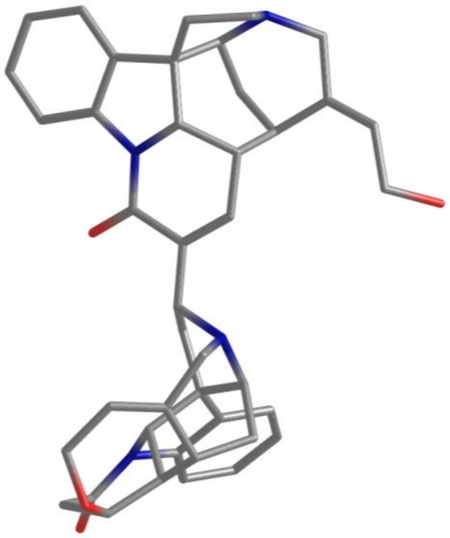

symmetry c1

| | | | |
|---|---|---|---|
| N | 2.622664000 | -1.597567000 | 0.092560000 |
| C | 3.500807000 | -0.733009000 | -0.722632000 |
| C | 5.385886000 | 0.045894000 | 0.853509000 |
| N | 6.229646000 | 0.988247000 | 0.086014000 |
| C | 6.300391000 | 0.564636000 | -1.312062000 |
| C | 6.007872000 | -0.927017000 | -1.247155000 |
| C | 4.924034000 | -1.021091000 | -0.172463000 |
| C | 4.733848000 | -2.368508000 | 0.473965000 |
| C | 5.687499000 | -3.251814000 | 0.938885000 |
| C | 5.276744000 | -4.420806000 | 1.573970000 |
| C | 3.924098000 | -4.681887000 | 1.743514000 |
| C | 2.949626000 | -3.799195000 | 1.286957000 |
| C | 3.378128000 | -2.643912000 | 0.647207000 |
| C | 4.198292000 | 0.737709000 | 1.514465000 |
| C | 3.491347000 | 1.598553000 | 0.465892000 |
| C | 3.023044000 | 0.717947000 | -0.747048000 |
| C | 1.529626000 | 0.751588000 | -0.884319000 |
| C | 2.652764000 | 4.247333000 | -0.876843000 |
| C | 4.058297000 | 3.815611000 | -0.593948000 |
| C | 4.439671000 | 2.695551000 | 0.012810000 |
| C | 5.900275000 | 2.390968000 | 0.280330000 |
| C | 1.269989000 | -1.467191000 | 0.133713000 |
| C | 0.723769000 | -0.232709000 | -0.496181000 |
| N | -4.600501000 | -0.614821000 | 1.235610000 |
| C | -3.797311000 | -0.379700000 | 0.021233000 |
| C | -2.369456000 | 1.641009000 | -0.753148000 |
| N | -1.261919000 | 0.921479000 | -1.430445000 |

| | | | |
|---|---|---|---|
| C | -0.781967000 | -0.154099000 | -0.557996000 |
| C | -1.430484000 | 0.126800000 | 0.802248000 |
| C | -2.791978000 | 0.726531000 | 0.419376000 |
| C | -3.481762000 | 1.354435000 | 1.602499000 |
| C | -3.205030000 | 2.538174000 | 2.257213000 |
| C | -3.929653000 | 2.866639000 | 3.400728000 |
| C | -4.905959000 | 2.004263000 | 3.878998000 |
| C | -5.194551000 | 0.804021000 | 3.234596000 |
| C | -4.475194000 | 0.500939000 | 2.089073000 |
| C | -3.474772000 | 1.966281000 | -1.745866000 |
| C | -4.064147000 | 0.648285000 | -2.263579000 |
| C | -4.785296000 | 0.022681000 | -1.073210000 |
| C | -5.732839000 | -1.146204000 | -1.393946000 |
| C | -4.242687000 | -2.432269000 | -2.829976000 |
| C | -3.040483000 | -1.549689000 | -2.989834000 |
| C | -2.935159000 | -0.244500000 | -2.769578000 |
| C | -1.591814000 | 0.442551000 | -2.782863000 |
| C | -5.712803000 | -1.410867000 | 1.126067000 |
| C | -5.797100000 | -2.132696000 | -0.205725000 |
| H | 3.452559000 | -1.110380000 | -1.749042000 |
| H | 5.992260000 | -0.451772000 | 1.613524000 |
| H | 7.282783000 | 0.782459000 | -1.729601000 |
| H | 5.559060000 | 1.078108000 | -1.938559000 |
| H | 5.696601000 | -1.352294000 | -2.198575000 |
| H | 6.896599000 | -1.459090000 | -0.908346000 |
| H | 6.740877000 | -3.037515000 | 0.815818000 |
| H | 6.011807000 | -5.123959000 | 1.937095000 |
| H | 3.614185000 | -5.590793000 | 2.239144000 |
| H | 1.901202000 | -4.001070000 | 1.418318000 |
| H | 4.530920000 | 1.364330000 | 2.340915000 |
| H | 3.526456000 | -0.009152000 | 1.936440000 |
| H | 2.606986000 | 2.051372000 | 0.912055000 |
| H | 3.435920000 | 1.162449000 | -1.651303000 |
| H | 1.078125000 | 1.649613000 | -1.288395000 |
| H | 1.930985000 | 3.618300000 | -0.351043000 |
| H | 2.437972000 | 4.166398000 | -1.943317000 |
| H | 4.817778000 | 4.519785000 | -0.920922000 |
| H | 6.141076000 | 2.662676000 | 1.310556000 |
| H | 6.539386000 | 2.997246000 | -0.359750000 |
| H | -3.271348000 | -1.293000000 | -0.251861000 |
| H | -1.976866000 | 2.565394000 | -0.322473000 |
| H | -1.144817000 | -1.126100000 | -0.912935000 |
| H | -1.507648000 | -0.764737000 | 1.419253000 |
| H | -0.851961000 | 0.877008000 | 1.344720000 |

| | | | |
|---|---|---|---|
| H | -2.432333000 | 3.201175000 | 1.891332000 |
| H | -3.726307000 | 3.791796000 | 3.919741000 |
| H | -5.457987000 | 2.264928000 | 4.770674000 |
| H | -5.951620000 | 0.133336000 | 3.603346000 |
| H | -3.072492000 | 2.576287000 | -2.552320000 |
| H | -4.248081000 | 2.557426000 | -1.256803000 |
| H | -4.778690000 | 0.840979000 | -3.064143000 |
| H | -5.409519000 | 0.804094000 | -0.630206000 |
| H | -6.729683000 | -0.734633000 | -1.537400000 |
| H | -4.041027000 | -3.155101000 | -2.030440000 |
| H | -4.372619000 | -3.023524000 | -3.736788000 |
| H | -2.150014000 | -2.092952000 | -3.292234000 |
| H | -0.804581000 | -0.239657000 | -3.098799000 |
| H | -1.581924000 | 1.285165000 | -3.473665000 |
| H | -4.955725000 | -2.824394000 | -0.255271000 |
| H | -6.713519000 | -2.712317000 | -0.225636000 |
| H | 2.670556000 | 5.740165000 | 0.372123000 |
| O | 0.540131000 | -2.284472000 | 0.675143000 |
| O | -6.549687000 | -1.516208000 | 1.999766000 |
| O | 2.448802000 | 5.615834000 | -0.553921000 |
| O | -5.477736000 | -1.782255000 | -2.631569000 |

Compound: **13-*chair-like***

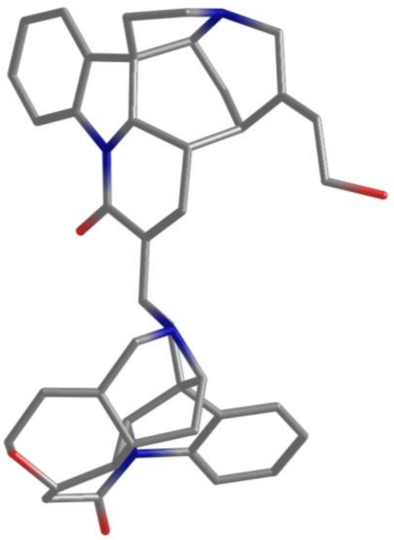

symmetry c1

| | | | |
|---|---|---|---|
| N | 2.589011000 | -1.581004000 | 0.175323000 |
| C | 3.473202000 | -0.752808000 | -0.670200000 |
| C | 5.367675000 | 0.061262000 | 0.876757000 |
| N | 6.217972000 | 0.970817000 | 0.077393000 |
| C | 6.282155000 | 0.500832000 | -1.306160000 |
| C | 5.977568000 | -0.985396000 | -1.191602000 |
| C | 4.894924000 | -1.034906000 | -0.112840000 |
| C | 4.693952000 | -2.358206000 | 0.578597000 |
| C | 5.640424000 | -3.234243000 | 1.071154000 |
| C | 5.220166000 | -4.376911000 | 1.746670000 |
| C | 3.865522000 | -4.619215000 | 1.927822000 |
| C | 2.898256000 | -3.743472000 | 1.443455000 |
| C | 3.336084000 | -2.614675000 | 0.764018000 |
| C | 4.187150000 | 0.784331000 | 1.516513000 |
| C | 3.485797000 | 1.616352000 | 0.441391000 |
| C | 3.008635000 | 0.700786000 | -0.742058000 |
| C | 1.515369000 | 0.743454000 | -0.879103000 |
| C | 2.666249000 | 4.229766000 | -0.980022000 |
| C | 4.068801000 | 3.794437000 | -0.687866000 |
| C | 4.442206000 | 2.690318000 | -0.047849000 |
| C | 5.900775000 | 2.381851000 | 0.226243000 |
| C | 1.237625000 | -1.435745000 | 0.217203000 |
| C | 0.701168000 | -0.219123000 | -0.455402000 |
| N | -4.688220000 | -0.519073000 | 1.254918000 |
| C | -3.795021000 | -0.419446000 | 0.079894000 |
| C | -2.434083000 | 1.619182000 | -0.787041000 |
| N | -1.274396000 | 0.934439000 | -1.408584000 |

| | | | |
|---|---|---|---|
| C | -0.803582000 | -0.129078000 | -0.518160000 |
| C | -1.448644000 | 0.198545000 | 0.830446000 |
| C | -2.824660000 | 0.743080000 | 0.421551000 |
| C | -3.528430000 | 1.434788000 | 1.556982000 |
| C | -3.232017000 | 2.640174000 | 2.163149000 |
| C | -3.979782000 | 3.047913000 | 3.264520000 |
| C | -5.000189000 | 2.241553000 | 3.749274000 |
| C | -5.307888000 | 1.021608000 | 3.153841000 |
| C | -4.562065000 | 0.638348000 | 2.048300000 |
| C | -3.558644000 | 1.831622000 | -1.789030000 |
| C | -4.014272000 | 0.465705000 | -2.309001000 |
| C | -4.707760000 | -0.230980000 | -1.133369000 |
| C | -5.420650000 | -1.564794000 | -1.386484000 |
| C | -4.211459000 | -2.273350000 | -3.334940000 |
| C | -2.915747000 | -1.511117000 | -3.361352000 |
| C | -2.816007000 | -0.279928000 | -2.877135000 |
| C | -1.510384000 | 0.465347000 | -2.776469000 |
| C | -5.819991000 | -1.284105000 | 1.163494000 |
| C | -5.859019000 | -2.193497000 | -0.046669000 |
| H | 3.419710000 | -1.163840000 | -1.683357000 |
| H | 5.971309000 | -0.416266000 | 1.651720000 |
| H | 7.265342000 | 0.696773000 | -1.732615000 |
| H | 5.543574000 | 0.999454000 | -1.947707000 |
| H | 5.661140000 | -1.439292000 | -2.127994000 |
| H | 6.862528000 | -1.513252000 | -0.836749000 |
| H | 6.695502000 | -3.034354000 | 0.938590000 |
| H | 5.949420000 | -5.074185000 | 2.132181000 |
| H | 3.548234000 | -5.507803000 | 2.454639000 |
| H | 1.848267000 | -3.930967000 | 1.583309000 |
| H | 4.526520000 | 1.434765000 | 2.321523000 |
| H | 3.509887000 | 0.057282000 | 1.963570000 |
| H | 2.605655000 | 2.090387000 | 0.873498000 |
| H | 3.424596000 | 1.111655000 | -1.660657000 |
| H | 1.071895000 | 1.631843000 | -1.312517000 |
| H | 1.940400000 | 3.624941000 | -0.431903000 |
| H | 2.446949000 | 4.116430000 | -2.042628000 |
| H | 4.833454000 | 4.481430000 | -1.038467000 |
| H | 6.146076000 | 2.685058000 | 1.246602000 |
| H | 6.543621000 | 2.961499000 | -0.434441000 |
| H | -3.239718000 | -1.351773000 | -0.036997000 |
| H | -2.107155000 | 2.586905000 | -0.399799000 |
| H | -1.176502000 | -1.107006000 | -0.850038000 |
| H | -1.501545000 | -0.663643000 | 1.489640000 |
| H | -0.883596000 | 0.988474000 | 1.329194000 |

| | | | |
|---|---|---|---|
| H | -2.425152000 | 3.258434000 | 1.792439000 |
| H | -3.760801000 | 3.989636000 | 3.746029000 |
| H | -5.571476000 | 2.562301000 | 4.608677000 |
| H | -6.097633000 | 0.394979000 | 3.530012000 |
| H | -3.216644000 | 2.471653000 | -2.600269000 |
| H | -4.384173000 | 2.349574000 | -1.301028000 |
| H | -4.752740000 | 0.598169000 | -3.104563000 |
| H | -5.499868000 | 0.457136000 | -0.819128000 |
| H | -6.295778000 | -1.375114000 | -2.015829000 |
| H | -4.100326000 | -3.234939000 | -3.829095000 |
| H | -5.001100000 | -1.720052000 | -3.856019000 |
| H | -2.036104000 | -2.012388000 | -3.746837000 |
| H | -0.677443000 | -0.172372000 | -3.068716000 |
| H | -1.507166000 | 1.321694000 | -3.452408000 |
| H | -5.161712000 | -3.010008000 | 0.150624000 |
| H | -6.855133000 | -2.618733000 | -0.103774000 |
| H | 2.698733000 | 5.761257000 | 0.220817000 |
| O | 0.502673000 | -2.224249000 | 0.792516000 |
| O | -6.708767000 | -1.261011000 | 1.992840000 |
| O | 2.476120000 | 5.609898000 | -0.700996000 |
| O | -4.626536000 | -2.557585000 | -2.007359000 |

Compound: **14a**

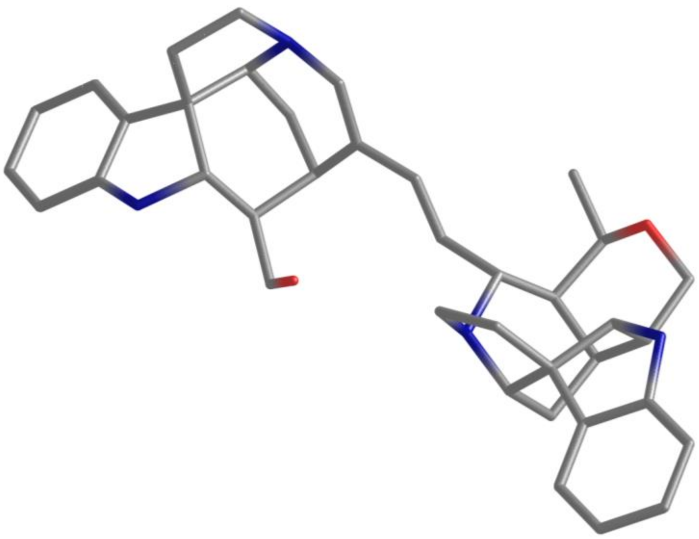

symmetry c1

| | | | |
|---|---|---|---|
| N | -4.871800000 | -2.191676000 | 0.152680000 |
| C | -4.112010000 | -0.955404000 | 0.406792000 |
| C | -4.859806000 | 1.510625000 | 0.577402000 |
| N | -4.053683000 | 1.810429000 | 1.761917000 |
| C | -4.739742000 | 1.178960000 | 2.898332000 |
| C | -5.557130000 | 0.006132000 | 2.293821000 |
| C | -5.250688000 | 0.040461000 | 0.776157000 |
| C | -6.389158000 | -0.494514000 | -0.054804000 |
| C | -7.579110000 | 0.082277000 | -0.445158000 |
| C | -8.495127000 | -0.675590000 | -1.177371000 |
| C | -8.205442000 | -1.995610000 | -1.494439000 |
| C | -7.009373000 | -2.590940000 | -1.099689000 |
| C | -6.105996000 | -1.825279000 | -0.378386000 |
| C | -4.103980000 | 1.814275000 | -0.697705000 |
| C | -2.843545000 | 0.949599000 | -0.720924000 |
| C | -3.295284000 | -0.525991000 | -0.824131000 |
| C | -2.173605000 | -1.523029000 | -1.119796000 |
| C | 0.224647000 | 0.924065000 | -0.480287000 |
| C | -0.586034000 | 1.221100000 | 0.538779000 |
| C | -2.038005000 | 1.269615000 | 0.520662000 |
| C | -2.689493000 | 1.613107000 | 1.649483000 |
| N | 5.957130000 | 0.405910000 | 1.283985000 |
| C | 4.681319000 | 0.566408000 | 0.562376000 |
| C | 3.316932000 | -1.106582000 | -0.871741000 |
| N | 1.980147000 | -0.680196000 | -0.454797000 |
| C | 1.841154000 | -1.319182000 | 0.860732000 |
| C | 3.259948000 | -1.359660000 | 1.499716000 |

| | | | |
|---|---|---|---|
| C | 4.212724000 | -0.905989000 | 0.365130000 |
| C | 5.530741000 | -1.638614000 | 0.348683000 |
| C | 5.846248000 | -2.917431000 | -0.062858000 |
| C | 7.161012000 | -3.369045000 | 0.060555000 |
| C | 8.132809000 | -2.536137000 | 0.598702000 |
| C | 7.824523000 | -1.247088000 | 1.025762000 |
| C | 6.514416000 | -0.810916000 | 0.894538000 |
| C | 3.792142000 | -0.411052000 | -2.121388000 |
| C | 3.837879000 | 1.073864000 | -1.832930000 |
| C | 4.873403000 | 1.370865000 | -0.739168000 |
| C | 4.835875000 | 2.863486000 | -0.434078000 |
| C | 1.331690000 | 3.827328000 | -0.719674000 |
| C | 2.586951000 | 3.088289000 | -1.131040000 |
| C | 2.436503000 | 1.595825000 | -1.476452000 |
| C | 1.719123000 | 0.782534000 | -0.377117000 |
| H | -4.378571000 | -2.944467000 | -0.301657000 |
| H | -3.433989000 | -1.103256000 | 1.245206000 |
| H | -5.767213000 | 2.117839000 | 0.636846000 |
| H | -5.404792000 | 1.899168000 | 3.371264000 |
| H | -4.011413000 | 0.853833000 | 3.635988000 |
| H | -5.297798000 | -0.957433000 | 2.726571000 |
| H | -6.621092000 | 0.167287000 | 2.455058000 |
| H | -7.804628000 | 1.110020000 | -0.190303000 |
| H | -9.427752000 | -0.235177000 | -1.497661000 |
| H | -8.918825000 | -2.575830000 | -2.062865000 |
| H | -6.791889000 | -3.619187000 | -1.352459000 |
| H | -3.846049000 | 2.872401000 | -0.726138000 |
| H | -4.741127000 | 1.596870000 | -1.555245000 |
| H | -2.259947000 | 1.188680000 | -1.610801000 |
| H | -3.963714000 | -0.571428000 | -1.691852000 |
| H | -1.572199000 | -1.151091000 | -1.954390000 |
| H | -2.607519000 | -2.467598000 | -1.449435000 |
| H | -0.179260000 | 0.690114000 | -1.460519000 |
| H | -0.129203000 | 1.450941000 | 1.499128000 |
| H | -2.137492000 | 1.791130000 | 2.565163000 |
| H | 6.573052000 | 1.206011000 | 1.279959000 |
| H | 3.983792000 | 1.104471000 | 1.200731000 |
| H | 3.249455000 | -2.183928000 | -1.048787000 |
| H | 1.117703000 | -0.785382000 | 1.473172000 |
| H | 1.466877000 | -2.332675000 | 0.719752000 |
| H | 3.502888000 | -2.371141000 | 1.818439000 |
| H | 3.349313000 | -0.712231000 | 2.369965000 |
| H | 5.086014000 | -3.566481000 | -0.478399000 |
| H | 7.423158000 | -4.365157000 | -0.264263000 |

| | | | |
|---|---|---|---|
| H | 9.149934000 | -2.891239000 | 0.688185000 |
| H | 8.585762000 | -0.605043000 | 1.446312000 |
| H | 3.110941000 | -0.626430000 | -2.945144000 |
| H | 4.781049000 | -0.783830000 | -2.393234000 |
| H | 4.157670000 | 1.614632000 | -2.727606000 |
| H | 5.857349000 | 1.116772000 | -1.144304000 |
| H | 5.476002000 | 3.121352000 | 0.409785000 |
| H | 5.176765000 | 3.432783000 | -1.306587000 |
| H | 1.544261000 | 4.893070000 | -0.663685000 |
| H | 0.530602000 | 3.661269000 | -1.437722000 |
| H | 0.989259000 | 3.491069000 | 0.255535000 |
| H | 2.992625000 | 3.565759000 | -2.033464000 |
| H | 1.832137000 | 1.530174000 | -2.386257000 |
| H | 2.034830000 | 1.177564000 | 0.596687000 |
| O | -1.352990000 | -1.837422000 | -0.012165000 |
| O | 3.529228000 | 3.271728000 | -0.080826000 |
| H | -0.728499000 | -1.114481000 | 0.115544000 |

Compound: **14b**

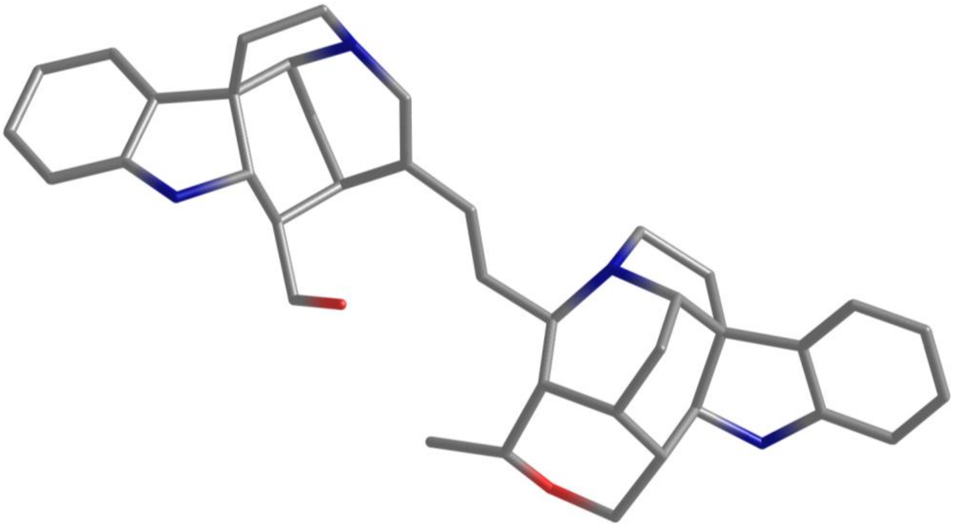

symmetry c1

| | | | |
|---|---|---|---|
| N | 5.466516000 | 1.120202000 | 1.278906000 |
| C | 4.434204000 | 0.188231000 | 0.793378000 |
| C | 4.575663000 | -1.882369000 | -0.738897000 |
| N | 3.695771000 | -2.707990000 | 0.089252000 |
| C | 4.481298000 | -3.103717000 | 1.267416000 |
| C | 5.579271000 | -2.017512000 | 1.419654000 |
| C | 5.300589000 | -1.001273000 | 0.285875000 |
| C | 6.547122000 | -0.284346000 | -0.166031000 |
| C | 7.574542000 | -0.682060000 | -0.995130000 |
| C | 8.654942000 | 0.178125000 | -1.200492000 |
| C | 8.688649000 | 1.411955000 | -0.565416000 |
| C | 7.659708000 | 1.821353000 | 0.279817000 |
| C | 6.589877000 | 0.960178000 | 0.470078000 |
| C | 3.796300000 | -1.138573000 | -1.801862000 |
| C | 2.775543000 | -0.241066000 | -1.101309000 |
| C | 3.567351000 | 0.822958000 | -0.306639000 |
| C | 2.708221000 | 1.959778000 | 0.239653000 |
| C | -0.246754000 | 0.172020000 | -0.502137000 |
| C | 0.467211000 | -0.864917000 | -0.048040000 |
| C | 1.883564000 | -1.126297000 | -0.255015000 |
| C | 2.414226000 | -2.234910000 | 0.300818000 |
| N | -6.191486000 | 0.824448000 | -1.217245000 |
| C | -4.804210000 | 0.750065000 | -0.723796000 |
| C | -3.603653000 | -1.029458000 | 0.718993000 |
| N | -2.307443000 | -0.975659000 | 0.049361000 |
| C | -2.539706000 | -1.767001000 | -1.165502000 |
| C | -4.029128000 | -1.551745000 | -1.567151000 |
| C | -4.643130000 | -0.760751000 | -0.384630000 |

| | | | |
|---|---|---|---|
| C | -6.064778000 | -1.148727000 | -0.065368000 |
| C | -6.575056000 | -2.260513000 | 0.573243000 |
| C | -7.957384000 | -2.392057000 | 0.714397000 |
| C | -8.802434000 | -1.413056000 | 0.209350000 |
| C | -8.299706000 | -0.290838000 | -0.444187000 |
| C | -6.924014000 | -0.172405000 | -0.574984000 |
| C | -3.682209000 | -0.113788000 | 1.912415000 |
| C | -3.451922000 | 1.297093000 | 1.419231000 |
| C | -4.581115000 | 1.709878000 | 0.462306000 |
| C | -4.285969000 | 3.110857000 | -0.057138000 |
| C | -0.667823000 | 3.296459000 | -0.401864000 |
| C | -1.957587000 | 2.879906000 | 0.267992000 |
| C | -2.053009000 | 1.433376000 | 0.788925000 |
| C | -1.739388000 | 0.347096000 | -0.275900000 |
| H | 5.171019000 | 2.063990000 | 1.474818000 |
| H | 3.793703000 | -0.117720000 | 1.618686000 |
| H | 5.310541000 | -2.547354000 | -1.200907000 |
| H | 4.934035000 | -4.078969000 | 1.098443000 |
| H | 3.833088000 | -3.175775000 | 2.136327000 |
| H | 5.565681000 | -1.535420000 | 2.394480000 |
| H | 6.564659000 | -2.457244000 | 1.279289000 |
| H | 7.547769000 | -1.647539000 | -1.484113000 |
| H | 9.463018000 | -0.115220000 | -1.854160000 |
| H | 9.527490000 | 2.073496000 | -0.730850000 |
| H | 7.693999000 | 2.784874000 | 0.768694000 |
| H | 3.297222000 | -1.852639000 | -2.455932000 |
| H | 4.484654000 | -0.547410000 | -2.406261000 |
| H | 2.173905000 | 0.275868000 | -1.850545000 |
| H | 4.247040000 | 1.290134000 | -1.028599000 |
| H | 2.080554000 | 2.343046000 | -0.571924000 |
| H | 3.343108000 | 2.787702000 | 0.555861000 |
| H | 0.236875000 | 0.926446000 | -1.108566000 |
| H | -0.062772000 | -1.612423000 | 0.531841000 |
| H | 1.811561000 | -2.859790000 | 0.949552000 |
| H | -6.606021000 | 1.744593000 | -1.242018000 |
| H | -4.129884000 | 1.033250000 | -1.529310000 |
| H | -3.737467000 | -2.067558000 | 1.038704000 |
| H | -1.839113000 | -1.478931000 | -1.944618000 |
| H | -2.366915000 | -2.819616000 | -0.940576000 |
| H | -4.533786000 | -2.508531000 | -1.684996000 |
| H | -4.138281000 | -1.007037000 | -2.503105000 |
| H | -5.914446000 | -3.024227000 | 0.963491000 |
| H | -8.369808000 | -3.253615000 | 1.218496000 |
| H | -9.871917000 | -1.519622000 | 0.325532000 |

| | | | |
|---|---|---|---|
| H | -8.963946000 | 0.466519000 | -0.836407000 |
| H | -2.926976000 | -0.398279000 | 2.645664000 |
| H | -4.662913000 | -0.205313000 | 2.382050000 |
| H | -3.482194000 | 1.992228000 | 2.262350000 |
| H | -5.506319000 | 1.735625000 | 1.045725000 |
| H | -4.994638000 | 3.418316000 | -0.826337000 |
| H | -4.339812000 | 3.835021000 | 0.763907000 |
| H | -0.677070000 | 4.372726000 | -0.562006000 |
| H | 0.183259000 | 3.039077000 | 0.225704000 |
| H | -0.563840000 | 2.816142000 | -1.372829000 |
| H | -2.103178000 | 3.518548000 | 1.149706000 |
| H | -1.324294000 | 1.327285000 | 1.597788000 |
| H | -2.153001000 | 0.714895000 | -1.225900000 |
| O | 1.925083000 | 1.624898000 | 1.369033000 |
| O | -3.004731000 | 3.162129000 | -0.652010000 |
| H | 1.263256000 | 0.975537000 | 1.102442000 |

Compound: **14c**

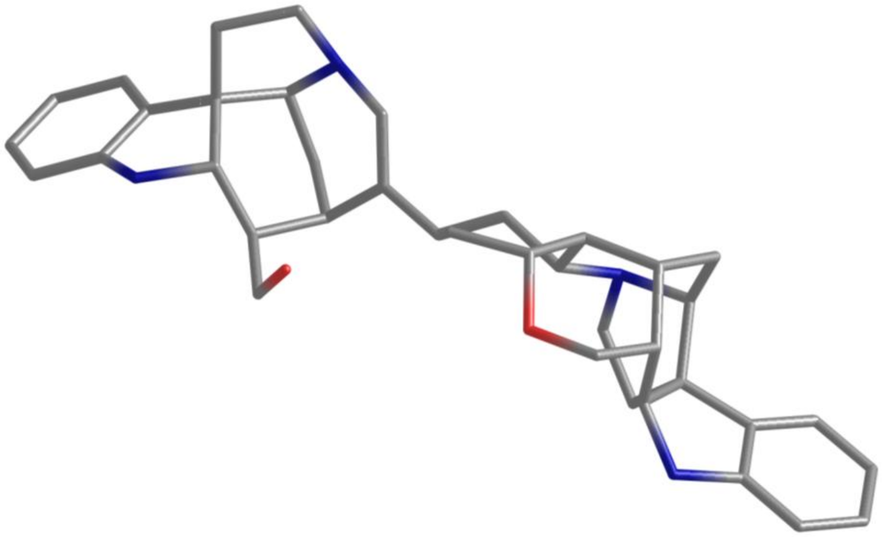

symmetry c1

| | | | |
|---|---|---|---|
| N | 5.752881000 | 1.102566000 | -1.365615000 |
| C | 4.655448000 | 0.640299000 | -0.497886000 |
| C | 4.574083000 | -1.075634000 | 1.407678000 |
| N | 3.703425000 | -0.228422000 | 2.229036000 |
| C | 4.528193000 | 0.886785000 | 2.724730000 |
| C | 5.757480000 | 0.943935000 | 1.782669000 |
| C | 5.431681000 | -0.056334000 | 0.653724000 |
| C | 6.658007000 | -0.550547000 | -0.065896000 |
| C | 7.597541000 | -1.497902000 | 0.283270000 |
| C | 8.682541000 | -1.724381000 | -0.565079000 |
| C | 8.808967000 | -0.993425000 | -1.738653000 |
| C | 7.870017000 | -0.029306000 | -2.097948000 |
| C | 6.794349000 | 0.182625000 | -1.248883000 |
| C | 3.789001000 | -2.019917000 | 0.524247000 |
| C | 2.843299000 | -1.184046000 | -0.333562000 |
| C | 3.687665000 | -0.283153000 | -1.262722000 |
| C | 2.830728000 | 0.524523000 | -2.241331000 |
| C | -0.499529000 | -0.054678000 | 1.078866000 |
| C | 0.517472000 | -0.242168000 | 0.238591000 |
| C | 1.924775000 | -0.417728000 | 0.597707000 |
| C | 2.440784000 | 0.063576000 | 1.745165000 |
| N | -5.748074000 | 0.395508000 | -1.814525000 |
| C | -4.613589000 | 0.454565000 | -0.874821000 |
| C | -4.115614000 | -0.923746000 | 1.267682000 |
| N | -2.665980000 | -1.044431000 | 1.116318000 |
| C | -2.547082000 | -2.121743000 | 0.127441000 |
| C | -3.750094000 | -1.970784000 | -0.849157000 |

| | | | |
|---|---|---|---|
| C | -4.680533000 | -0.932939000 | -0.166941000 |
| C | -6.148473000 | -1.258693000 | -0.285383000 |
| C | -6.916401000 | -2.204438000 | 0.363006000 |
| C | -8.269412000 | -2.322672000 | 0.041792000 |
| C | -8.827018000 | -1.497468000 | -0.925684000 |
| C | -8.060525000 | -0.545753000 | -1.593084000 |
| C | -6.717719000 | -0.438299000 | -1.261902000 |
| C | -4.517983000 | 0.260578000 | 2.107903000 |
| C | -4.016619000 | 1.507327000 | 1.413168000 |
| C | -4.723732000 | 1.675372000 | 0.060932000 |
| C | -4.152545000 | 2.908386000 | -0.628245000 |
| C | -0.607798000 | 2.941361000 | 0.207301000 |
| C | -2.080142000 | 2.743829000 | 0.502997000 |
| C | -2.483763000 | 1.476307000 | 1.279709000 |
| C | -1.929795000 | 0.166865000 | 0.671713000 |
| H | 5.499069000 | 1.390458000 | -2.298554000 |
| H | 4.104525000 | 1.498342000 | -0.117106000 |
| H | 5.223034000 | -1.639364000 | 2.083625000 |
| H | 4.839597000 | 0.701413000 | 3.751103000 |
| H | 3.948377000 | 1.806072000 | 2.707299000 |
| H | 5.948117000 | 1.941874000 | 1.394311000 |
| H | 6.651702000 | 0.611583000 | 2.307094000 |
| H | 7.497343000 | -2.062552000 | 1.201464000 |
| H | 9.422305000 | -2.468700000 | -0.310085000 |
| H | 9.650702000 | -1.176182000 | -2.391880000 |
| H | 7.976736000 | 0.534176000 | -3.014252000 |
| H | 3.226163000 | -2.723330000 | 1.136437000 |
| H | 4.485918000 | -2.587639000 | -0.093708000 |
| H | 2.244477000 | -1.838650000 | -0.968460000 |
| H | 4.294972000 | -0.962796000 | -1.870197000 |
| H | 2.068443000 | -0.133858000 | -2.670718000 |
| H | 3.439776000 | 0.884890000 | -3.070288000 |
| H | -0.336817000 | -0.110589000 | 2.151008000 |
| H | 0.296320000 | -0.258413000 | -0.828379000 |
| H | 1.851902000 | 0.708017000 | 2.388421000 |
| H | -6.067949000 | 1.279462000 | -2.182550000 |
| H | -3.692266000 | 0.543738000 | -1.445916000 |
| H | -4.446776000 | -1.848674000 | 1.748567000 |
| H | -1.580721000 | -2.080730000 | -0.367802000 |
| H | -2.612594000 | -3.078048000 | 0.645474000 |
| H | -4.262828000 | -2.922291000 | -0.973247000 |
| H | -3.447110000 | -1.632158000 | -1.838254000 |
| H | -6.478900000 | -2.849333000 | 1.114436000 |
| H | -8.883070000 | -3.053883000 | 0.547002000 |

| | | | |
|---|---|---|---|
| H | -9.876582000 | -1.591682000 | -1.166936000 |
| H | -8.500860000 | 0.091078000 | -2.347496000 |
| H | -4.084819000 | 0.167729000 | 3.104214000 |
| H | -5.604035000 | 0.285851000 | 2.211476000 |
| H | -4.262635000 | 2.388697000 | 2.011375000 |
| H | -5.785008000 | 1.843307000 | 0.266761000 |
| H | -4.540674000 | 3.026839000 | -1.639952000 |
| H | -4.408864000 | 3.808585000 | -0.057603000 |
| H | -0.445552000 | 3.954527000 | -0.154867000 |
| H | -0.001315000 | 2.778582000 | 1.096968000 |
| H | -0.285044000 | 2.248315000 | -0.565743000 |
| H | -2.429648000 | 3.588252000 | 1.112606000 |
| H | -2.075222000 | 1.569542000 | 2.290630000 |
| H | -1.963342000 | 0.260703000 | -0.421303000 |
| O | 2.231252000 | 1.680590000 | -1.680777000 |
| O | -2.748271000 | 2.806649000 | -0.751497000 |
| H | 1.841548000 | 1.448892000 | -0.831954000 |

Compound: **14d**

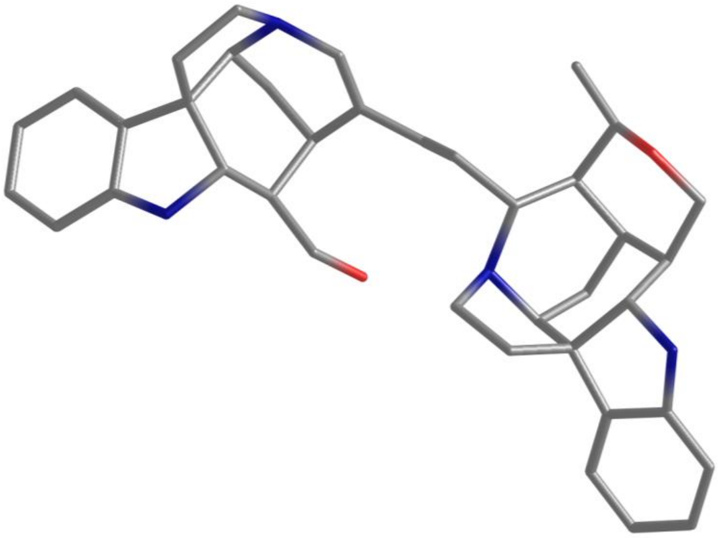

symmetry c1

| | | | |
|---|---|---|---|
| N | 4.494435000 | -1.884834000 | -1.127039000 |
| C | 3.885497000 | -0.624993000 | -0.658830000 |
| C | 4.913214000 | 1.425755000 | 0.540555000 |
| N | 4.171909000 | 2.436322000 | -0.219235000 |
| C | 4.784386000 | 2.464661000 | -1.556687000 |
| C | 5.458880000 | 1.079028000 | -1.752454000 |
| C | 5.134588000 | 0.287013000 | -0.463485000 |
| C | 6.193655000 | -0.727766000 | -0.118089000 |
| C | 7.438776000 | -0.578885000 | 0.457438000 |
| C | 8.250659000 | -1.701705000 | 0.623465000 |
| C | 7.805111000 | -2.948017000 | 0.203784000 |
| C | 6.553884000 | -3.108620000 | -0.385842000 |
| C | 5.755938000 | -1.985085000 | -0.540497000 |
| C | 4.189513000 | 1.010957000 | 1.801592000 |
| C | 2.827391000 | 0.460830000 | 1.402030000 |
| C | 3.018075000 | -0.841080000 | 0.598760000 |
| C | 1.688356000 | -1.461759000 | 0.155712000 |
| C | -0.236161000 | 1.505887000 | -0.355653000 |
| C | 0.627452000 | 1.427660000 | 0.650346000 |
| C | 2.087168000 | 1.500434000 | 0.591199000 |
| C | 2.777632000 | 2.353875000 | -0.175350000 |
| N | -5.966132000 | -0.078293000 | -1.260356000 |
| C | -4.666838000 | 0.369920000 | -0.727768000 |
| C | -2.922918000 | -0.877130000 | 0.739388000 |
| N | -1.697818000 | -0.339159000 | 0.137491000 |
| C | -1.585916000 | -1.130385000 | -1.101358000 |
| C | -3.033497000 | -1.423506000 | -1.585550000 |

| | | | |
|---|---|---|---|
| C | -3.941920000 | -0.973807000 | -0.411199000 |
| C | -5.111709000 | -1.892798000 | -0.158651000 |
| C | -5.165953000 | -3.140664000 | 0.428469000 |
| C | -6.391926000 | -3.801519000 | 0.513654000 |
| C | -7.538318000 | -3.204885000 | 0.005740000 |
| C | -7.494306000 | -1.950464000 | -0.596575000 |
| C | -6.269075000 | -1.304616000 | -0.672371000 |
| C | -3.378652000 | -0.079533000 | 1.934923000 |
| C | -3.679455000 | 1.328698000 | 1.466781000 |
| C | -4.849977000 | 1.319586000 | 0.473596000 |
| C | -5.064427000 | 2.743321000 | -0.026919000 |
| C | -1.718508000 | 4.165640000 | -0.326194000 |
| C | -2.813445000 | 3.366682000 | 0.347545000 |
| C | -2.414809000 | 1.981946000 | 0.886578000 |
| C | -1.694741000 | 1.123775000 | -0.174383000 |
| H | 3.911180000 | -2.706149000 | -1.070152000 |
| H | 3.253024000 | -0.212571000 | -1.446609000 |
| H | 5.889309000 | 1.854982000 | 0.783079000 |
| H | 5.531781000 | 3.254873000 | -1.603145000 |
| H | 4.026188000 | 2.674593000 | -2.306556000 |
| H | 5.107062000 | 0.557270000 | -2.639934000 |
| H | 6.536870000 | 1.197052000 | -1.843817000 |
| H | 7.785359000 | 0.394502000 | 0.780297000 |
| H | 9.223976000 | -1.602077000 | 1.080749000 |
| H | 8.438126000 | -3.813809000 | 0.339050000 |
| H | 6.214111000 | -4.082455000 | -0.709470000 |
| H | 4.075877000 | 1.869157000 | 2.462939000 |
| H | 4.781571000 | 0.257079000 | 2.322283000 |
| H | 2.242127000 | 0.215318000 | 2.287631000 |
| H | 3.537338000 | -1.548506000 | 1.254028000 |
| H | 1.874520000 | -2.450417000 | -0.275834000 |
| H | 1.262199000 | -0.840919000 | -0.635796000 |
| H | 0.114055000 | 1.719860000 | -1.359381000 |
| H | 0.235711000 | 1.133354000 | 1.622082000 |
| H | 2.269568000 | 3.088043000 | -0.789706000 |
| H | -6.702986000 | 0.611798000 | -1.269959000 |
| H | -4.138568000 | 0.908789000 | -1.510926000 |
| H | -2.680274000 | -1.899554000 | 1.043827000 |
| H | -0.983926000 | -0.602052000 | -1.836966000 |
| H | -1.077165000 | -2.066232000 | -0.867081000 |
| H | -3.158924000 | -2.486029000 | -1.781451000 |
| H | -3.284960000 | -0.890733000 | -2.500454000 |
| H | -4.270894000 | -3.605456000 | 0.821861000 |
| H | -6.449724000 | -4.775226000 | 0.977234000 |

| | | | |
|---|---|---|---|
| H | -8.485404000 | -3.720878000 | 0.079103000 |
| H | -8.390301000 | -1.493039000 | -0.992238000 |
| H | -2.596197000 | -0.078580000 | 2.694966000 |
| H | -4.267191000 | -0.543436000 | 2.365955000 |
| H | -3.990149000 | 1.941303000 | 2.317112000 |
| H | -5.740491000 | 0.997211000 | 1.020987000 |
| H | -5.821394000 | 2.789330000 | -0.809928000 |
| H | -5.389294000 | 3.386106000 | 0.799311000 |
| H | -2.092167000 | 5.159346000 | -0.564682000 |
| H | -0.849860000 | 4.254950000 | 0.322687000 |
| H | -1.407711000 | 3.686726000 | -1.253155000 |
| H | -3.183733000 | 3.936154000 | 1.210573000 |
| H | -1.726197000 | 2.142485000 | 1.718861000 |
| H | -2.205774000 | 1.292553000 | -1.127386000 |
| O | 0.763230000 | -1.597430000 | 1.215560000 |
| O | -3.875875000 | 3.259379000 | -0.592849000 |
| H | -0.038015000 | -1.089256000 | 0.990096000 |

Compound: **15a**

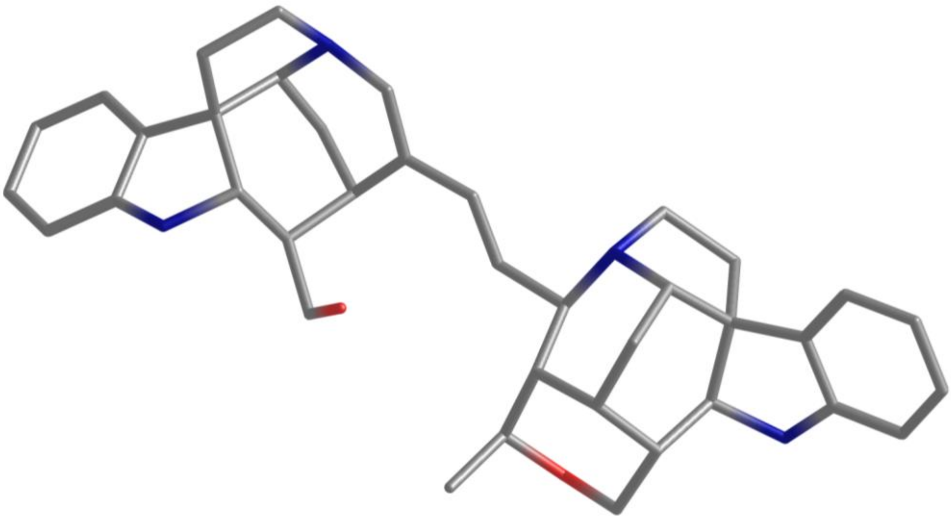

symmetry c1

| | | | |
|---|---|---|---|
| N | 5.230329000 | 1.182257000 | 1.278416000 |
| C | 4.302898000 | 0.161631000 | 0.760639000 |
| C | 4.682896000 | -1.893414000 | -0.750658000 |
| N | 3.850442000 | -2.792080000 | 0.051933000 |
| C | 4.626256000 | -3.107790000 | 1.261116000 |
| C | 5.616953000 | -1.927291000 | 1.442423000 |
| C | 5.289203000 | -0.946197000 | 0.291287000 |
| C | 6.481486000 | -0.121421000 | -0.122074000 |
| C | 7.568035000 | -0.425464000 | -0.914681000 |
| C | 8.574175000 | 0.527552000 | -1.084907000 |
| C | 8.476599000 | 1.758925000 | -0.451829000 |
| C | 7.386754000 | 2.074683000 | 0.356301000 |
| C | 6.391446000 | 1.121997000 | 0.511208000 |
| C | 3.879003000 | -1.224914000 | -1.844402000 |
| C | 2.758815000 | -0.418301000 | -1.187284000 |
| C | 3.420765000 | 0.717025000 | -0.370721000 |
| C | 2.451073000 | 1.779786000 | 0.148916000 |
| C | -0.255121000 | -0.199731000 | -0.663813000 |
| C | 0.491466000 | -1.191462000 | -0.167196000 |
| C | 1.921512000 | -1.374297000 | -0.363065000 |
| C | 2.526265000 | -2.428192000 | 0.220724000 |
| N | -6.094026000 | 0.877660000 | -1.276640000 |
| C | -4.697680000 | 0.727204000 | -0.826946000 |
| C | -3.638043000 | -1.016101000 | 0.798750000 |
| N | -2.337509000 | -1.196340000 | 0.151697000 |
| C | -2.671696000 | -2.106335000 | -0.951920000 |
| C | -4.095487000 | -1.710482000 | -1.443000000 |
| C | -4.643261000 | -0.758826000 | -0.344454000 |

| | | | |
|---|---|---|---|
| C | -6.086349000 | -1.009406000 | 0.016883000 |
| C | -6.664792000 | -2.031452000 | 0.742219000 |
| C | -8.048855000 | -2.046511000 | 0.918005000 |
| C | -8.829139000 | -1.042413000 | 0.360695000 |
| C | -8.258541000 | -0.012220000 | -0.381647000 |
| C | -6.880893000 | -0.009589000 | -0.546616000 |
| C | -3.622592000 | 0.037527000 | 1.876253000 |
| C | -3.252245000 | 1.356113000 | 1.230836000 |
| C | -4.335365000 | 1.787845000 | 0.235589000 |
| C | -3.870086000 | 3.073279000 | -0.440257000 |
| C | -1.168094000 | 3.692679000 | 0.758114000 |
| C | -1.546354000 | 2.549540000 | -0.172639000 |
| C | -1.867323000 | 1.247829000 | 0.578411000 |
| C | -1.718023000 | 0.030714000 | -0.369287000 |
| H | 4.846832000 | 2.095511000 | 1.466187000 |
| H | 3.664720000 | -0.199361000 | 1.565054000 |
| H | 5.489788000 | -2.492367000 | -1.182096000 |
| H | 3.955764000 | -3.232215000 | 2.107131000 |
| H | 5.168929000 | -4.040436000 | 1.117681000 |
| H | 6.641958000 | -2.278464000 | 1.341323000 |
| H | 5.524946000 | -1.443198000 | 2.412124000 |
| H | 7.644869000 | -1.389709000 | -1.401101000 |
| H | 9.427430000 | 0.306553000 | -1.709082000 |
| H | 9.258218000 | 2.492984000 | -0.589865000 |
| H | 7.317694000 | 3.037171000 | 0.843556000 |
| H | 3.467095000 | -1.981990000 | -2.510784000 |
| H | 4.533557000 | -0.578081000 | -2.429337000 |
| H | 2.144112000 | 0.037068000 | -1.964567000 |
| H | 4.080738000 | 1.240559000 | -1.072045000 |
| H | 1.794072000 | 2.093672000 | -0.669739000 |
| H | 3.007473000 | 2.666484000 | 0.453332000 |
| H | 0.201713000 | 0.531682000 | -1.319858000 |
| H | -0.001948000 | -1.926514000 | 0.460647000 |
| H | 1.959737000 | -3.092423000 | 0.863236000 |
| H | -6.435057000 | 1.824739000 | -1.353974000 |
| H | -4.044344000 | 0.867393000 | -1.684968000 |
| H | -3.893970000 | -1.985873000 | 1.234795000 |
| H | -1.916633000 | -2.049943000 | -1.731666000 |
| H | -2.682362000 | -3.126826000 | -0.569688000 |
| H | -4.726433000 | -2.591855000 | -1.536714000 |
| H | -4.078072000 | -1.217658000 | -2.413427000 |
| H | -6.055502000 | -2.815445000 | 1.173792000 |
| H | -8.513144000 | -2.837351000 | 1.488669000 |
| H | -9.900547000 | -1.057353000 | 0.504209000 |

| | | | |
|---|---|---|---|
| H | -8.872550000 | 0.763815000 | -0.816922000 |
| H | -2.901744000 | -0.234080000 | 2.647949000 |
| H | -4.608790000 | 0.100032000 | 2.339769000 |
| H | -3.195491000 | 2.131204000 | 1.997672000 |
| H | -5.240662000 | 2.001047000 | 0.811830000 |
| H | -4.549227000 | 3.379903000 | -1.236270000 |
| H | -3.826938000 | 3.886457000 | 0.290927000 |
| H | -1.911791000 | 3.872236000 | 1.531820000 |
| H | -0.225640000 | 3.449490000 | 1.247536000 |
| H | -1.039706000 | 4.609252000 | 0.184810000 |
| H | -0.703331000 | 2.382675000 | -0.842222000 |
| H | -1.138299000 | 1.130227000 | 1.384131000 |
| H | -2.182509000 | 0.316890000 | -1.323033000 |
| O | 1.696414000 | 1.393732000 | 1.281177000 |
| O | -2.609575000 | 2.895815000 | -1.060193000 |
| H | 1.101701000 | 0.682119000 | 1.017626000 |

Compound: **15b**

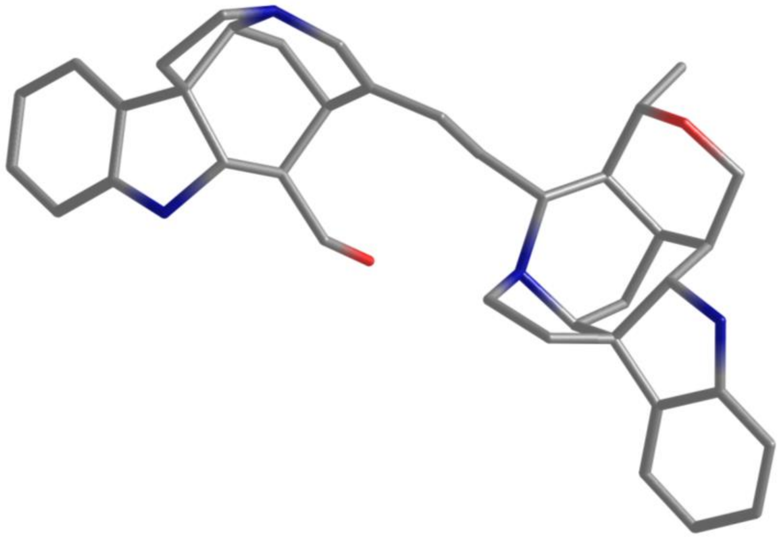

symmetry c1

| | | | |
|---|---|---|---|
| N | -4.704107000 | -2.156816000 | 0.514698000 |
| C | -4.040742000 | -0.844406000 | 0.641985000 |
| C | -4.929249000 | 1.557089000 | 0.309551000 |
| N | -4.237042000 | 2.107167000 | 1.477460000 |
| C | -4.968680000 | 1.622202000 | 2.657940000 |
| C | -5.700461000 | 0.332015000 | 2.199872000 |
| C | -5.259902000 | 0.121639000 | 0.732628000 |
| C | -6.287523000 | -0.615979000 | -0.085140000 |
| C | -7.465563000 | -0.188562000 | -0.661991000 |
| C | -8.263753000 | -1.110292000 | -1.340401000 |
| C | -7.872431000 | -2.440068000 | -1.420847000 |
| C | -6.689516000 | -2.883501000 | -0.835222000 |
| C | -5.903541000 | -1.955749000 | -0.168850000 |
| C | -4.099825000 | 1.667107000 | -0.949162000 |
| C | -2.784820000 | 0.929179000 | -0.722302000 |
| C | -3.083424000 | -0.576536000 | -0.537152000 |
| C | -1.824187000 | -1.418848000 | -0.344783000 |
| C | 0.194505000 | 1.223793000 | -0.388289000 |
| C | -0.646164000 | 1.527307000 | 0.601068000 |
| C | -2.098191000 | 1.528920000 | 0.486179000 |
| C | -2.855163000 | 1.993513000 | 1.497187000 |
| N | 5.773821000 | 0.055707000 | 1.476743000 |
| C | 4.538411000 | 0.392203000 | 0.744414000 |
| C | 3.100534000 | -1.023542000 | -0.892578000 |
| N | 1.779876000 | -0.539782000 | -0.469146000 |
| C | 1.557703000 | -1.271547000 | 0.791193000 |
| C | 2.950321000 | -1.503460000 | 1.440562000 |

| | | | |
|---|---|---|---|
| C | 3.965705000 | -1.011389000 | 0.381072000 |
| C | 5.225011000 | -1.839299000 | 0.317759000 |
| C | 5.456633000 | -3.087741000 | -0.222652000 |
| C | 6.728651000 | -3.651512000 | -0.117375000 |
| C | 7.742491000 | -2.958983000 | 0.531125000 |
| C | 7.518628000 | -1.702385000 | 1.087010000 |
| C | 6.249826000 | -1.153350000 | 0.972764000 |
| C | 3.677617000 | -0.245063000 | -2.048685000 |
| C | 3.836462000 | 1.198180000 | -1.614900000 |
| C | 4.836142000 | 1.311630000 | -0.457874000 |
| C | 4.875447000 | 2.767696000 | -0.005927000 |
| C | 2.923366000 | 4.238502000 | -1.768784000 |
| C | 2.621073000 | 3.201666000 | -0.696728000 |
| C | 2.465833000 | 1.780880000 | -1.257336000 |
| C | 1.657615000 | 0.932607000 | -0.256603000 |
| H | -4.128020000 | -2.915326000 | 0.182512000 |
| H | -3.470268000 | -0.813482000 | 1.572140000 |
| H | -5.869873000 | 2.103857000 | 0.198234000 |
| H | -4.273871000 | 1.446600000 | 3.474934000 |
| H | -5.689558000 | 2.371192000 | 2.981176000 |
| H | -6.778176000 | 0.478884000 | 2.236477000 |
| H | -5.461548000 | -0.532624000 | 2.815356000 |
| H | -7.769968000 | 0.848230000 | -0.594894000 |
| H | -9.184228000 | -0.789151000 | -1.805309000 |
| H | -8.494272000 | -3.147306000 | -1.951711000 |
| H | -6.390806000 | -3.920226000 | -0.903678000 |
| H | -3.914832000 | 2.715512000 | -1.180580000 |
| H | -4.654918000 | 1.229371000 | -1.779876000 |
| H | -2.158998000 | 1.032493000 | -1.606002000 |
| H | -3.578488000 | -0.916319000 | -1.453622000 |
| H | -2.103687000 | -2.453940000 | -0.124336000 |
| H | -1.281009000 | -1.045139000 | 0.525820000 |
| H | -0.191084000 | 1.025590000 | -1.380989000 |
| H | -0.240258000 | 1.738992000 | 1.588269000 |
| H | -2.392526000 | 2.354227000 | 2.409237000 |
| H | 6.447819000 | 0.801599000 | 1.570964000 |
| H | 3.865040000 | 0.914130000 | 1.420886000 |
| H | 2.961471000 | -2.069652000 | -1.180011000 |
| H | 0.875873000 | -0.715026000 | 1.429657000 |
| H | 1.090046000 | -2.230335000 | 0.562671000 |
| H | 3.100564000 | -2.561553000 | 1.644428000 |
| H | 3.070419000 | -0.968836000 | 2.380678000 |
| H | 4.664570000 | -3.626895000 | -0.726752000 |
| H | 6.924839000 | -4.624750000 | -0.542734000 |

| | | | |
|---|---|---|---|
| H | 8.726568000 | -3.400231000 | 0.606859000 |
| H | 8.312439000 | -1.170161000 | 1.592417000 |
| H | 3.016079000 | -0.318404000 | -2.912640000 |
| H | 4.642126000 | -0.674491000 | -2.324901000 |
| H | 4.241171000 | 1.781865000 | -2.444380000 |
| H | 5.820889000 | 1.036689000 | -0.847430000 |
| H | 5.493948000 | 2.895371000 | 0.883056000 |
| H | 5.299162000 | 3.394514000 | -0.796537000 |
| H | 3.104990000 | 5.206603000 | -1.305726000 |
| H | 3.789088000 | 3.977846000 | -2.374716000 |
| H | 2.067088000 | 4.329411000 | -2.434965000 |
| H | 1.689543000 | 3.483344000 | -0.204073000 |
| H | 1.887186000 | 1.840574000 | -2.183416000 |
| H | 1.985290000 | 1.197897000 | 0.754423000 |
| O | -1.001375000 | -1.412309000 | -1.495680000 |
| O | 3.585314000 | 3.225135000 | 0.357129000 |
| H | -0.102187000 | -1.140990000 | -1.253149000 |

Compound: **15c**

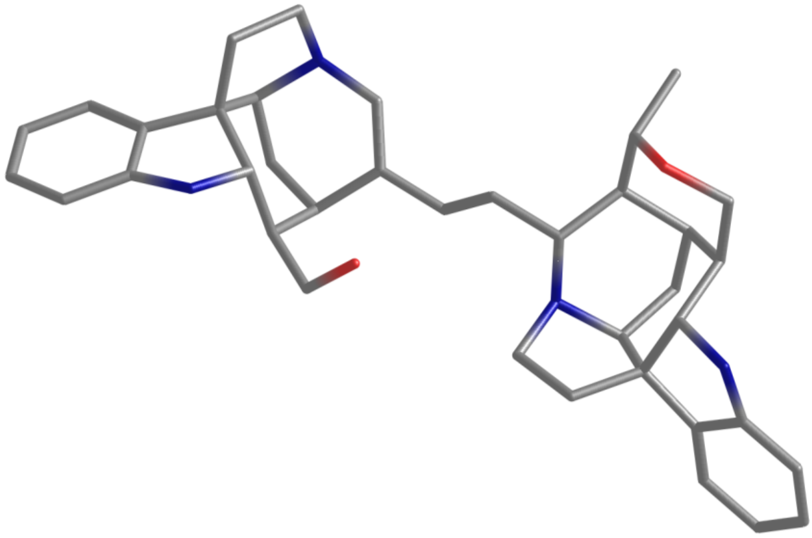

symmetry c1

| | | | |
|---|---|---|---|
| N | 5.054051000 | -1.168578000 | -1.700064000 |
| C | 4.224080000 | -0.395429000 | -0.760135000 |
| C | 4.837191000 | 0.999622000 | 1.326554000 |
| N | 3.947835000 | 2.119807000 | 1.012331000 |
| C | 4.590318000 | 2.851934000 | -0.090018000 |
| C | 5.504379000 | 1.822574000 | -0.808814000 |
| C | 5.287795000 | 0.491250000 | -0.049520000 |
| C | 6.496691000 | -0.408400000 | -0.095649000 |
| C | 7.678218000 | -0.379385000 | 0.614872000 |
| C | 8.669343000 | -1.319551000 | 0.327111000 |
| C | 8.461399000 | -2.262349000 | -0.670326000 |
| C | 7.274835000 | -2.297316000 | -1.399189000 |
| C | 6.296641000 | -1.361077000 | -1.100012000 |
| C | 4.151166000 | -0.029632000 | 2.197170000 |
| C | 2.936989000 | -0.550612000 | 1.435136000 |
| C | 3.440599000 | -1.314813000 | 0.191549000 |
| C | 2.338608000 | -2.073803000 | -0.557386000 |
| C | -0.244826000 | 0.957185000 | 0.129799000 |
| C | 0.595242000 | 0.382645000 | 0.992947000 |
| C | 2.040641000 | 0.617956000 | 1.083856000 |
| C | 2.595990000 | 1.820104000 | 0.864531000 |
| N | -5.790151000 | -0.416256000 | -1.664817000 |
| C | -4.577937000 | 0.091717000 | -0.996707000 |
| C | -3.498573000 | -0.562923000 | 1.274967000 |
| N | -2.092496000 | -0.443718000 | 0.889030000 |
| C | -1.862596000 | -1.693221000 | 0.149677000 |
| C | -3.196153000 | -2.042543000 | -0.574248000 |

| | | | |
|---|---|---|---|
| C | -4.234222000 | -1.049684000 | 0.010077000 |
| C | -5.593935000 | -1.658739000 | 0.246629000 |
| C | -6.033384000 | -2.526901000 | 1.225385000 |
| C | -7.356382000 | -2.970159000 | 1.201297000 |
| C | -8.212954000 | -2.542052000 | 0.195876000 |
| C | -7.778871000 | -1.673748000 | -0.802217000 |
| C | -6.461831000 | -1.239787000 | -0.764094000 |
| C | -4.052830000 | 0.699022000 | 1.884924000 |
| C | -3.949906000 | 1.800746000 | 0.850997000 |
| C | -4.830001000 | 1.476646000 | -0.363331000 |
| C | -4.624745000 | 2.572110000 | -1.403387000 |
| C | -2.637888000 | 4.483086000 | -0.195544000 |
| C | -2.395183000 | 3.055858000 | -0.668577000 |
| C | -2.479627000 | 2.025132000 | 0.469779000 |
| C | -1.736552000 | 0.724807000 | 0.072347000 |
| H | 4.620049000 | -1.971600000 | -2.129234000 |
| H | 3.519513000 | 0.220946000 | -1.313719000 |
| H | 5.710280000 | 1.412199000 | 1.839676000 |
| H | 3.833372000 | 3.275373000 | -0.744679000 |
| H | 5.186875000 | 3.669612000 | 0.310673000 |
| H | 6.546633000 | 2.124135000 | -0.725324000 |
| H | 5.273048000 | 1.716673000 | -1.866321000 |
| H | 7.839330000 | 0.360729000 | 1.388372000 |
| H | 9.596565000 | -1.314552000 | 0.880832000 |
| H | 9.232559000 | -2.988546000 | -0.886308000 |
| H | 7.121223000 | -3.035931000 | -2.173386000 |
| H | 3.847962000 | 0.428649000 | 3.137854000 |
| H | 4.849724000 | -0.836571000 | 2.420638000 |
| H | 2.379247000 | -1.254119000 | 2.056676000 |
| H | 4.134497000 | -2.076494000 | 0.564009000 |
| H | 1.676624000 | -2.551431000 | 0.172890000 |
| H | 2.780998000 | -2.873159000 | -1.151553000 |
| H | 0.152462000 | 1.654540000 | -0.601326000 |
| H | 0.187715000 | -0.361672000 | 1.670616000 |
| H | 1.982789000 | 2.673049000 | 0.600096000 |
| H | -6.348057000 | 0.269819000 | -2.152106000 |
| H | -3.789936000 | 0.191484000 | -1.739548000 |
| H | -3.544471000 | -1.376795000 | 2.004262000 |
| H | -1.023417000 | -1.588446000 | -0.533976000 |
| H | -1.612119000 | -2.479221000 | 0.862357000 |
| H | -3.488096000 | -3.068489000 | -0.360335000 |
| H | -3.122704000 | -1.942854000 | -1.655437000 |
| H | -5.363324000 | -2.862956000 | 2.006422000 |
| H | -7.714656000 | -3.644004000 | 1.965509000 |

| | | | |
|---|---|---|---|
| H | -9.237585000 | -2.886498000 | 0.185261000 |
| H | -8.450259000 | -1.347367000 | -1.584122000 |
| H | -3.488228000 | 0.954152000 | 2.782116000 |
| H | -5.092792000 | 0.537689000 | 2.173512000 |
| H | -4.326359000 | 2.732445000 | 1.277433000 |
| H | -5.870263000 | 1.500551000 | -0.025664000 |
| H | -5.142722000 | 2.345744000 | -2.335479000 |
| H | -5.014999000 | 3.523417000 | -1.029672000 |
| H | -3.570191000 | 4.593741000 | 0.354176000 |
| H | -1.826531000 | 4.788705000 | 0.462918000 |
| H | -2.660699000 | 5.155256000 | -1.051242000 |
| H | -1.403395000 | 3.021824000 | -1.116843000 |
| H | -1.973871000 | 2.438224000 | 1.346072000 |
| H | -1.977267000 | 0.539801000 | -0.984122000 |
| O | 1.591397000 | -1.293358000 | -1.472721000 |
| O | -3.256618000 | 2.694318000 | -1.746784000 |
| H | 1.087206000 | -0.636152000 | -0.979252000 |

Compound: **15d**

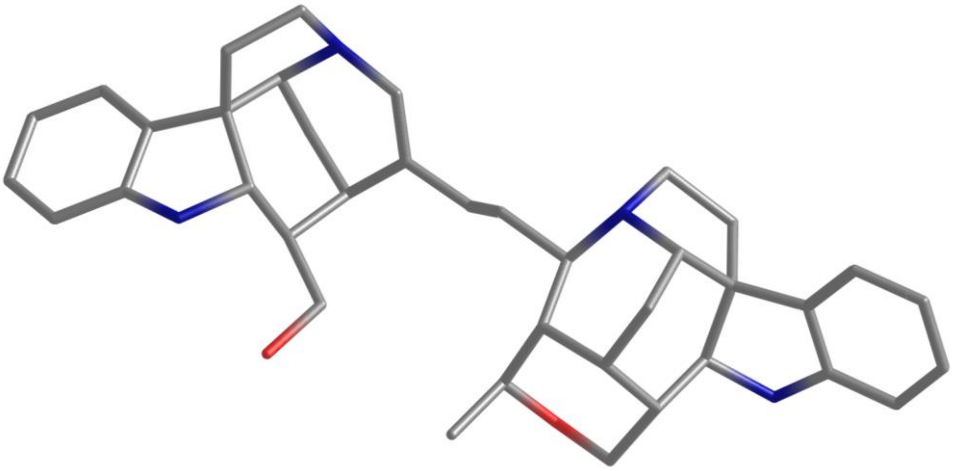

symmetry c1

| | | | |
|---|---|---|---|
| N | 5.108382000 | 1.190650000 | 1.181186000 |
| C | 4.191627000 | 0.214329000 | 0.566883000 |
| C | 4.635486000 | -1.933667000 | -0.809542000 |
| N | 3.625968000 | -2.757554000 | -0.142797000 |
| C | 4.186341000 | -3.088004000 | 1.175556000 |
| C | 5.186792000 | -1.949422000 | 1.513727000 |
| C | 5.137763000 | -0.993604000 | 0.295842000 |
| C | 6.448827000 | -0.288218000 | 0.058064000 |
| C | 7.619669000 | -0.720577000 | -0.528918000 |
| C | 8.718974000 | 0.139160000 | -0.569794000 |
| C | 8.626681000 | 1.408522000 | -0.015156000 |
| C | 7.452063000 | 1.852872000 | 0.587069000 |
| C | 6.365231000 | 0.991116000 | 0.616706000 |
| C | 4.073143000 | -1.239913000 | -2.031110000 |
| C | 2.926877000 | -0.343229000 | -1.575954000 |
| C | 3.507503000 | 0.781307000 | -0.691807000 |
| C | 2.434270000 | 1.800074000 | -0.320145000 |
| C | -0.327153000 | -0.763379000 | 0.187898000 |
| C | 0.500347000 | -0.767878000 | -0.854457000 |
| C | 1.909205000 | -1.181094000 | -0.830543000 |
| C | 2.320597000 | -2.265748000 | -0.158541000 |
| N | -5.848809000 | 0.815787000 | -1.488104000 |
| C | -4.554967000 | 0.593772000 | -0.816532000 |
| C | -3.932828000 | -0.988569000 | 1.156844000 |
| N | -2.592607000 | -1.389921000 | 0.725560000 |
| C | -2.887956000 | -2.408306000 | -0.289481000 |
| C | -4.186336000 | -1.952508000 | -1.018185000 |
| C | -4.747995000 | -0.800990000 | -0.140670000 |
| C | -6.245790000 | -0.842445000 | 0.037567000 |

| | | | |
|---|---|---|---|
| C | -7.037297000 | -1.675988000 | 0.801561000 |
| C | -8.423700000 | -1.519516000 | 0.770729000 |
| C | -8.992941000 | -0.536163000 | -0.027340000 |
| C | -8.205816000 | 0.304432000 | -0.809743000 |
| C | -6.829162000 | 0.137640000 | -0.768145000 |
| C | -3.923147000 | 0.204290000 | 2.079240000 |
| C | -3.322066000 | 1.373515000 | 1.326750000 |
| C | -4.215720000 | 1.756799000 | 0.140251000 |
| C | -3.525471000 | 2.878872000 | -0.627277000 |
| C | -0.911469000 | 3.393899000 | 0.801979000 |
| C | -1.327697000 | 2.162197000 | 0.010545000 |
| C | -1.889097000 | 1.036690000 | 0.893065000 |
| C | -1.759628000 | -0.313479000 | 0.150636000 |
| H | 4.756165000 | 2.136817000 | 1.203957000 |
| H | 3.417666000 | -0.065892000 | 1.282591000 |
| H | 5.459042000 | -2.594765000 | -1.092736000 |
| H | 3.387123000 | -3.182784000 | 1.905630000 |
| H | 4.708559000 | -4.041613000 | 1.120589000 |
| H | 6.191699000 | -2.352191000 | 1.622595000 |
| H | 4.938517000 | -1.426638000 | 2.434793000 |
| H | 7.690186000 | -1.712900000 | -0.955974000 |
| H | 9.639788000 | -0.182083000 | -1.033810000 |
| H | 9.481441000 | 2.069452000 | -0.052055000 |
| H | 7.389253000 | 2.842533000 | 1.017390000 |
| H | 3.719053000 | -1.983485000 | -2.744143000 |
| H | 4.860251000 | -0.656512000 | -2.509845000 |
| H | 2.447077000 | 0.119739000 | -2.441065000 |
| H | 4.269863000 | 1.296717000 | -1.288574000 |
| H | 1.756968000 | 1.372688000 | 0.419617000 |
| H | 1.838997000 | 2.049040000 | -1.204111000 |
| H | 0.030700000 | -1.070948000 | 1.166737000 |
| H | 0.126639000 | -0.402564000 | -1.810083000 |
| H | 1.612424000 | -2.873454000 | 0.391373000 |
| H | -6.056216000 | 1.769414000 | -1.746779000 |
| H | -3.777835000 | 0.539663000 | -1.574955000 |
| H | -4.354649000 | -1.853412000 | 1.676262000 |
| H | -2.040098000 | -2.534657000 | -0.958271000 |
| H | -3.062000000 | -3.359359000 | 0.212466000 |
| H | -4.898641000 | -2.772492000 | -1.078216000 |
| H | -3.996593000 | -1.611330000 | -2.034247000 |
| H | -6.592494000 | -2.444102000 | 1.421327000 |
| H | -9.053435000 | -2.160874000 | 1.369530000 |
| H | -10.067322000 | -0.417279000 | -0.043133000 |
| H | -8.655589000 | 1.065635000 | -1.431773000 |

| | | | |
|---|---|---|---|
| H | -3.339479000 | -0.025880000 | 2.970974000 |
| H | -4.943580000 | 0.431952000 | 2.392221000 |
| H | -3.275195000 | 2.243843000 | 1.984638000 |
| H | -5.156659000 | 2.135088000 | 0.550458000 |
| H | -4.065460000 | 3.136684000 | -1.538643000 |
| H | -3.471879000 | 3.779096000 | -0.007462000 |
| H | -1.706023000 | 3.774304000 | 1.441418000 |
| H | -0.061434000 | 3.146784000 | 1.436725000 |
| H | -0.607476000 | 4.185599000 | 0.119315000 |
| H | -0.449166000 | 1.786899000 | -0.512065000 |
| H | -1.278792000 | 0.975902000 | 1.798946000 |
| H | -2.025923000 | -0.135234000 | -0.898184000 |
| O | 2.956771000 | 2.985951000 | 0.268275000 |
| O | -2.232598000 | 2.483320000 | -1.046347000 |
| H | 3.399644000 | 3.494968000 | -0.415207000 |

Compound: **16a**

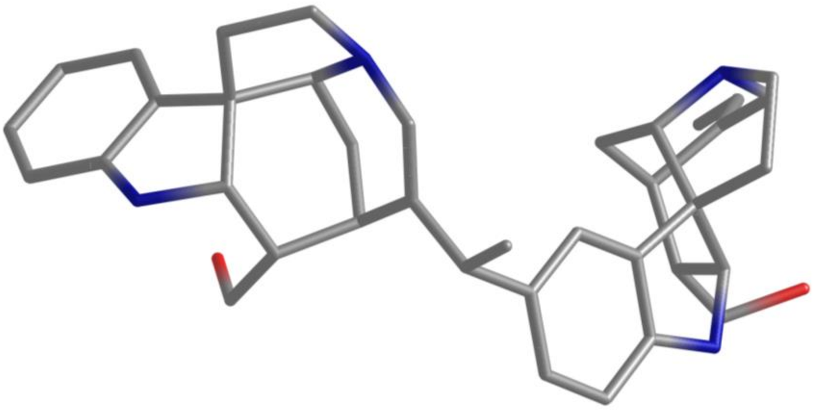

symmetry c1

| | | | |
|---|---|---|---|
| N | -5.910041000 | -0.430093000 | -0.724212000 |
| C | -4.589969000 | -0.786037000 | -0.183198000 |
| C | -2.976542000 | 0.451478000 | 1.411795000 |
| N | -2.710599000 | -0.723119000 | 2.246394000 |
| C | -3.933403000 | -0.962454000 | 3.029165000 |
| C | -5.082201000 | -0.256918000 | 2.262743000 |
| C | -4.430894000 | 0.257373000 | 0.958806000 |
| C | -5.174900000 | 1.433311000 | 0.381233000 |
| C | -5.136242000 | 2.779706000 | 0.680207000 |
| C | -5.971212000 | 3.656003000 | -0.016698000 |
| C | -6.833215000 | 3.168380000 | -0.989403000 |
| C | -6.887447000 | 1.809928000 | -1.296236000 |
| C | -6.049278000 | 0.950639000 | -0.601852000 |
| C | -1.969685000 | 0.574217000 | 0.290758000 |
| C | -2.071264000 | -0.680233000 | -0.575665000 |
| C | -3.456744000 | -0.791992000 | -1.250612000 |
| C | -3.610202000 | 0.147080000 | -2.454665000 |
| C | -1.077644000 | -4.315907000 | 0.536245000 |
| C | -1.115413000 | -3.060975000 | -0.335503000 |
| C | -1.809944000 | -1.883781000 | 0.304104000 |
| C | -2.172546000 | -1.840978000 | 1.588893000 |
| N | 4.209379000 | -1.648696000 | -1.741425000 |
| C | 4.511607000 | -0.620030000 | -0.728063000 |
| C | 3.068983000 | 0.223718000 | 1.285669000 |
| N | 4.110447000 | 0.433314000 | 2.294012000 |
| C | 4.372225000 | -0.932791000 | 2.757276000 |
| C | 4.226225000 | -1.864384000 | 1.518851000 |
| C | 3.537068000 | -0.983383000 | 0.440137000 |
| C | 2.428486000 | -1.686795000 | -0.304080000 |
| C | 1.150658000 | -2.012650000 | 0.097578000 |
| C | 0.290902000 | -2.684704000 | -0.778081000 |

| | | | |
|---|---|---|---|
| C | 0.757037000 | -3.015844000 | -2.044728000 |
| C | 2.050037000 | -2.704101000 | -2.462764000 |
| C | 2.880021000 | -2.035814000 | -1.577911000 |
| C | 2.759492000 | 1.471352000 | 0.493628000 |
| C | 4.019942000 | 1.889760000 | -0.260410000 |
| C | 4.339866000 | 0.798737000 | -1.305995000 |
| C | 5.532273000 | 1.176380000 | -2.184986000 |
| C | 5.768704000 | 4.475331000 | -0.043218000 |
| C | 5.794842000 | 3.286024000 | 0.867254000 |
| C | 5.093085000 | 2.155453000 | 0.772089000 |
| C | 5.340724000 | 1.033517000 | 1.752439000 |
| H | -6.165473000 | -0.839804000 | -1.609622000 |
| H | -4.632094000 | -1.785145000 | 0.246157000 |
| H | -2.938220000 | 1.332455000 | 2.059034000 |
| H | -4.099031000 | -2.031526000 | 3.137696000 |
| H | -3.828870000 | -0.536480000 | 4.025853000 |
| H | -5.453761000 | 0.589962000 | 2.836912000 |
| H | -5.924507000 | -0.913620000 | 2.055884000 |
| H | -4.461014000 | 3.156928000 | 1.437170000 |
| H | -5.941837000 | 4.714061000 | 0.196737000 |
| H | -7.470556000 | 3.854525000 | -1.528923000 |
| H | -7.554045000 | 1.440518000 | -2.062779000 |
| H | -0.966211000 | 0.643737000 | 0.713475000 |
| H | -2.162576000 | 1.474797000 | -0.284141000 |
| H | -1.315253000 | -0.645437000 | -1.363488000 |
| H | -3.508870000 | -1.784155000 | -1.712811000 |
| H | -2.859497000 | -0.153231000 | -3.185382000 |
| H | -4.586917000 | -0.009190000 | -2.921962000 |
| H | -0.619130000 | -5.135457000 | -0.013748000 |
| H | -0.482398000 | -4.145401000 | 1.433509000 |
| H | -2.079923000 | -4.617778000 | 0.839022000 |
| H | -1.671364000 | -3.307750000 | -1.246570000 |
| H | -2.035154000 | -2.693580000 | 2.241448000 |
| H | 4.506416000 | -1.443551000 | -2.683839000 |
| H | 5.544643000 | -0.741155000 | -0.414637000 |
| H | 2.176549000 | -0.094517000 | 1.830834000 |
| H | 3.624474000 | -1.192900000 | 3.505348000 |
| H | 5.351038000 | -0.994183000 | 3.227557000 |
| H | 5.184963000 | -2.236140000 | 1.162276000 |
| H | 3.612415000 | -2.728888000 | 1.761359000 |
| H | 0.792677000 | -1.742538000 | 1.084873000 |
| H | 0.092876000 | -3.527348000 | -2.730469000 |
| H | 2.389447000 | -2.970805000 | -3.454025000 |
| H | 2.432998000 | 2.262503000 | 1.167870000 |

| | | | |
|---|---|---|---|
| H | 1.949435000 | 1.266901000 | -0.208329000 |
| H | 3.817691000 | 2.804406000 | -0.813657000 |
| H | 3.464604000 | 0.771372000 | -1.964195000 |
| H | 5.369660000 | 2.177961000 | -2.595978000 |
| H | 5.611802000 | 0.493334000 | -3.030933000 |
| H | 6.766473000 | 4.665211000 | -0.441261000 |
| H | 5.091437000 | 4.358799000 | -0.883885000 |
| H | 5.476749000 | 5.370103000 | 0.507124000 |
| H | 6.489913000 | 3.365106000 | 1.697609000 |
| H | 5.931196000 | 1.404661000 | 2.589028000 |
| H | 5.957408000 | 0.273300000 | 1.252799000 |
| H | -4.101045000 | 1.883083000 | -1.701320000 |
| H | 6.763315000 | 1.729691000 | -0.783359000 |
| O | -3.390099000 | 1.530145000 | -2.245157000 |
| O | 6.780683000 | 1.108056000 | -1.518151000 |

Compound: **16b**

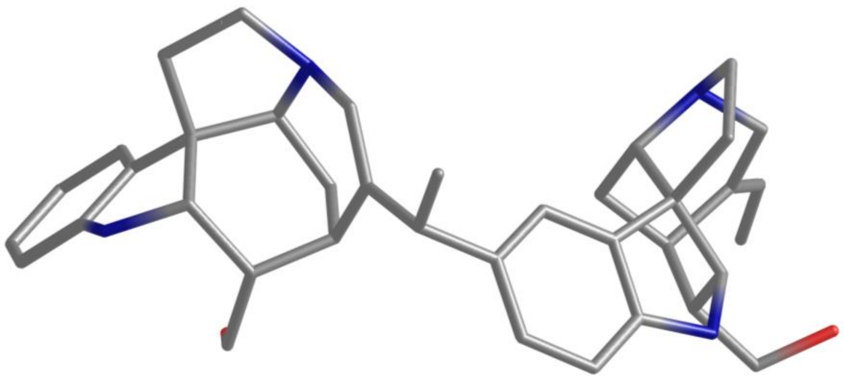

symmetry c1

| | | | |
|---|---|---|---|
| N | -5.545269000 | 1.442140000 | -1.422809000 |
| C | -4.617685000 | 0.325879000 | -1.097995000 |
| C | -4.618558000 | -1.475817000 | 0.798164000 |
| N | -4.168371000 | -2.622078000 | 0.007695000 |
| C | -5.337845000 | -3.025710000 | -0.777385000 |
| C | -6.160535000 | -1.734117000 | -1.025149000 |
| C | -5.452975000 | -0.632265000 | -0.188354000 |
| C | -6.458062000 | 0.325566000 | 0.402077000 |
| C | -7.294030000 | 0.195803000 | 1.494994000 |
| C | -8.214441000 | 1.207175000 | 1.766485000 |
| C | -8.296575000 | 2.321274000 | 0.941757000 |
| C | -7.451024000 | 2.462529000 | -0.155598000 |
| C | -6.536390000 | 1.456006000 | -0.406984000 |
| C | -3.446404000 | -0.755493000 | 1.428818000 |
| C | -2.568797000 | -0.219509000 | 0.301690000 |
| C | -3.322198000 | 0.883523000 | -0.463911000 |
| C | -3.535793000 | 2.118663000 | 0.420516000 |
| C | -0.640310000 | -2.291433000 | -2.419880000 |
| C | -0.871830000 | -1.223079000 | -1.351060000 |
| C | -2.181094000 | -1.360162000 | -0.612592000 |
| C | -2.987730000 | -2.418016000 | -0.726153000 |
| N | 3.666486000 | -0.937159000 | 2.062324000 |
| C | 4.590662000 | -0.127848000 | 1.246136000 |
| C | 4.208113000 | 1.031135000 | -1.067941000 |
| N | 4.966737000 | 2.269197000 | -0.878919000 |
| C | 4.049109000 | 3.073610000 | -0.066107000 |
| C | 3.312906000 | 2.089734000 | 0.889273000 |
| C | 3.622602000 | 0.677740000 | 0.318723000 |
| C | 2.423358000 | -0.237790000 | 0.273369000 |
| C | 1.326454000 | -0.237377000 | -0.566028000 |
| C | 0.309660000 | -1.179627000 | -0.393308000 |

| | | | |
|---|---|---|---|
| C | 0.425264000 | -2.102613000 | 0.642464000 |
| C | 1.517826000 | -2.109528000 | 1.504285000 |
| C | 2.515859000 | -1.165926000 | 1.308754000 |
| C | 5.024814000 | -0.064022000 | -1.710615000 |
| C | 6.189736000 | -0.405150000 | -0.783888000 |
| C | 5.610998000 | -1.018408000 | 0.510168000 |
| C | 6.708119000 | -1.491477000 | 1.463860000 |
| C | 9.280027000 | -0.152496000 | -1.199698000 |
| C | 8.322886000 | 0.942570000 | -0.840906000 |
| C | 7.011156000 | 0.853810000 | -0.614557000 |
| C | 6.242384000 | 2.075737000 | -0.169918000 |
| H | -5.930854000 | 1.391799000 | -2.355872000 |
| H | -4.343403000 | -0.187873000 | -2.015874000 |
| H | -5.293062000 | -1.860349000 | 1.567798000 |
| H | -5.024791000 | -3.519011000 | -1.693610000 |
| H | -5.928025000 | -3.733956000 | -0.197669000 |
| H | -7.184697000 | -1.866216000 | -0.682868000 |
| H | -6.201951000 | -1.458719000 | -2.077814000 |
| H | -7.247096000 | -0.679982000 | 2.128975000 |
| H | -8.868714000 | 1.122967000 | 2.621954000 |
| H | -9.014981000 | 3.097960000 | 1.161728000 |
| H | -7.495620000 | 3.339892000 | -0.785543000 |
| H | -2.883044000 | -1.455145000 | 2.046175000 |
| H | -3.806348000 | 0.042826000 | 2.076923000 |
| H | -1.659835000 | 0.220291000 | 0.716927000 |
| H | -2.690308000 | 1.227297000 | -1.287430000 |
| H | -4.369267000 | 1.971732000 | 1.113091000 |
| H | -2.638953000 | 2.279135000 | 1.019753000 |
| H | 0.290114000 | -2.090700000 | -2.947722000 |
| H | -0.557547000 | -3.279397000 | -1.966526000 |
| H | -1.453403000 | -2.309845000 | -3.145073000 |
| H | -0.892971000 | -0.252053000 | -1.857478000 |
| H | -2.752276000 | -3.238560000 | -1.391256000 |
| H | 4.068595000 | -1.736608000 | 2.528686000 |
| H | 5.132826000 | 0.543339000 | 1.906207000 |
| H | 3.364934000 | 1.286858000 | -1.714709000 |
| H | 3.329904000 | 3.552539000 | -0.729248000 |
| H | 4.591988000 | 3.856231000 | 0.459003000 |
| H | 3.654954000 | 2.175582000 | 1.918863000 |
| H | 2.242478000 | 2.282387000 | 0.885405000 |
| H | 1.242933000 | 0.492986000 | -1.363345000 |
| H | -0.363857000 | -2.831088000 | 0.782530000 |
| H | 1.582592000 | -2.830852000 | 2.307128000 |
| H | 5.390042000 | 0.271917000 | -2.680458000 |

| | | | |
|---|---|---|---|
| H | 4.398005000 | -0.942963000 | -1.868193000 |
| H | 6.801904000 | -1.174819000 | -1.249236000 |
| H | 5.074267000 | -1.915908000 | 0.183879000 |
| H | 7.402593000 | -2.137522000 | 0.917119000 |
| H | 6.280257000 | -2.091552000 | 2.267327000 |
| H | 9.745515000 | 0.048826000 | -2.164911000 |
| H | 10.087185000 | -0.199875000 | -0.467518000 |
| H | 8.814826000 | -1.132526000 | -1.245826000 |
| H | 8.773659000 | 1.925433000 | -0.743116000 |
| H | 6.851854000 | 2.965072000 | -0.324415000 |
| H | 6.077862000 | 1.999872000 | 0.914052000 |
| H | -4.447386000 | 3.087052000 | -0.950516000 |
| H | 7.832349000 | 0.096399000 | 1.423721000 |
| O | -3.733668000 | 3.300094000 | -0.335003000 |
| O | 7.406386000 | -0.436960000 | 2.102492000 |

Compound: **16c**

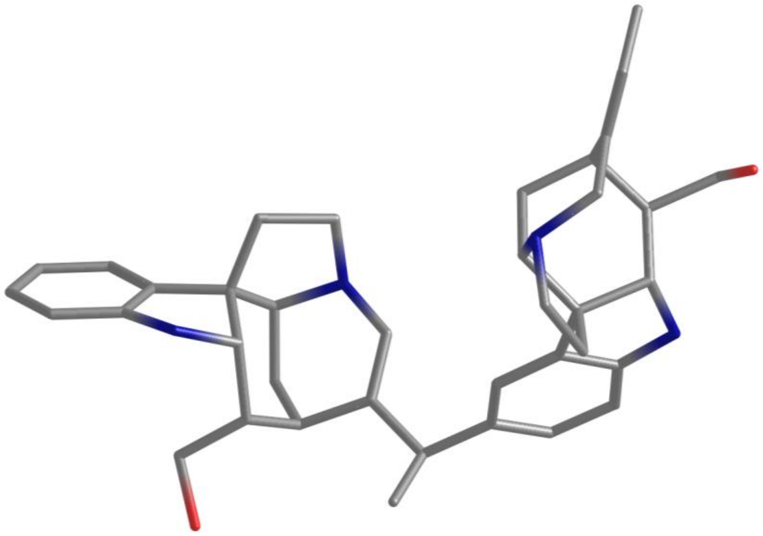

symmetry c1

| | | | |
|---|---|---|---|
| N | 4.202290000 | -1.293193000 | 1.667489000 |
| C | 3.466472000 | -0.435400000 | 0.724183000 |
| C | 3.529338000 | -0.227804000 | -1.844992000 |
| N | 2.073660000 | -0.232182000 | -2.003796000 |
| C | 1.623433000 | -1.605167000 | -1.725940000 |
| C | 2.755580000 | -2.269462000 | -0.901946000 |
| C | 3.768992000 | -1.134688000 | -0.630664000 |
| C | 5.155184000 | -1.669734000 | -0.379824000 |
| C | 6.143990000 | -2.089319000 | -1.243684000 |
| C | 7.336236000 | -2.594224000 | -0.719231000 |
| C | 7.508459000 | -2.680100000 | 0.655101000 |
| C | 6.512245000 | -2.266740000 | 1.538040000 |
| C | 5.338703000 | -1.758952000 | 1.004532000 |
| C | 4.058861000 | 1.182561000 | -1.708064000 |
| C | 3.415630000 | 1.814450000 | -0.473999000 |
| C | 3.875743000 | 1.056535000 | 0.794921000 |
| C | 5.358761000 | 1.300406000 | 1.064413000 |
| C | 1.465961000 | 3.322578000 | 1.319446000 |
| C | 1.116099000 | 2.975490000 | -0.135102000 |
| C | 1.911531000 | 1.789767000 | -0.654063000 |
| C | 1.365764000 | 0.789077000 | -1.353400000 |
| N | -4.488683000 | 2.218919000 | -0.648502000 |
| C | -4.653018000 | 0.885697000 | -0.038933000 |
| C | -2.888004000 | -0.644192000 | 1.139210000 |
| N | -3.703638000 | -1.223533000 | 2.208354000 |
| C | -3.751168000 | -0.132511000 | 3.186873000 |
| C | -3.795521000 | 1.197264000 | 2.378715000 |

| | | | |
|---|---|---|---|
| C | -3.425749000 | 0.789624000 | 0.925906000 |
| C | -2.445792000 | 1.721637000 | 0.255617000 |
| C | -1.090150000 | 1.879346000 | 0.446271000 |
| C | -0.377998000 | 2.820567000 | -0.307836000 |
| C | -1.074361000 | 3.590821000 | -1.230748000 |
| C | -2.449364000 | 3.459103000 | -1.422748000 |
| C | -3.126481000 | 2.515197000 | -0.669827000 |
| C | -2.854356000 | -1.499366000 | -0.104600000 |
| C | -4.276272000 | -1.614093000 | -0.650148000 |
| C | -4.726898000 | -0.212736000 | -1.118120000 |
| C | -6.109426000 | -0.240622000 | -1.770569000 |
| C | -6.133976000 | -4.106137000 | -1.006195000 |
| C | -5.871903000 | -3.346067000 | 0.257778000 |
| C | -5.120476000 | -2.257007000 | 0.427711000 |
| C | -5.064629000 | -1.585850000 | 1.779475000 |
| H | 4.334585000 | -0.928595000 | 2.598870000 |
| H | 2.399704000 | -0.491017000 | 0.935587000 |
| H | 3.961443000 | -0.715423000 | -2.723835000 |
| H | 0.677389000 | -1.580497000 | -1.189873000 |
| H | 1.467159000 | -2.146564000 | -2.657920000 |
| H | 3.240333000 | -3.049071000 | -1.486944000 |
| H | 2.403317000 | -2.719310000 | 0.023910000 |
| H | 6.004156000 | -2.026052000 | -2.315233000 |
| H | 8.124337000 | -2.917657000 | -1.383106000 |
| H | 8.434501000 | -3.071065000 | 1.052835000 |
| H | 6.656464000 | -2.332389000 | 2.607501000 |
| H | 3.799617000 | 1.756011000 | -2.597519000 |
| H | 5.145518000 | 1.160404000 | -1.639011000 |
| H | 3.747069000 | 2.847724000 | -0.377722000 |
| H | 3.353093000 | 1.478835000 | 1.655508000 |
| H | 5.664631000 | 0.780625000 | 1.974643000 |
| H | 5.988145000 | 0.930853000 | 0.252735000 |
| H | 0.905965000 | 4.201737000 | 1.634032000 |
| H | 1.213324000 | 2.501565000 | 1.990668000 |
| H | 2.527569000 | 3.537424000 | 1.432300000 |
| H | 1.418689000 | 3.838803000 | -0.738060000 |
| H | 0.295461000 | 0.729111000 | -1.502740000 |
| H | -4.997158000 | 2.379565000 | -1.505310000 |
| H | -5.582086000 | 0.877912000 | 0.523784000 |
| H | -1.877324000 | -0.548708000 | 1.544801000 |
| H | -2.844081000 | -0.169046000 | 3.788788000 |
| H | -4.600441000 | -0.257438000 | 3.854758000 |
| H | -4.774543000 | 1.671411000 | 2.411553000 |
| H | -3.075908000 | 1.909831000 | 2.775426000 |

| | | | |
|---|---|---|---|
| H | -0.562347000 | 1.261179000 | 1.163503000 |
| H | -0.529974000 | 4.314645000 | -1.823766000 |
| H | -2.967553000 | 4.075360000 | -2.144234000 |
| H | -2.452569000 | -2.483268000 | 0.135545000 |
| H | -2.202147000 | -1.036414000 | -0.847348000 |
| H | -4.270101000 | -2.252007000 | -1.531364000 |
| H | -4.016959000 | 0.065174000 | -1.904790000 |
| H | -6.123718000 | -1.014651000 | -2.544575000 |
| H | -6.315464000 | 0.709093000 | -2.264816000 |
| H | -7.205730000 | -4.143494000 | -1.205883000 |
| H | -5.648125000 | -3.674989000 | -1.876372000 |
| H | -5.798969000 | -5.138866000 | -0.906286000 |
| H | -6.377522000 | -3.736712000 | 1.135607000 |
| H | -5.490466000 | -2.247164000 | 2.532780000 |
| H | -5.711593000 | -0.698056000 | 1.746701000 |
| H | 6.453195000 | 2.881005000 | 1.406831000 |
| H | -7.051195000 | -1.289352000 | -0.429265000 |
| O | 5.527931000 | 2.702506000 | 1.229343000 |
| O | -7.172145000 | -0.433786000 | -0.853450000 |

Compound: **16d**

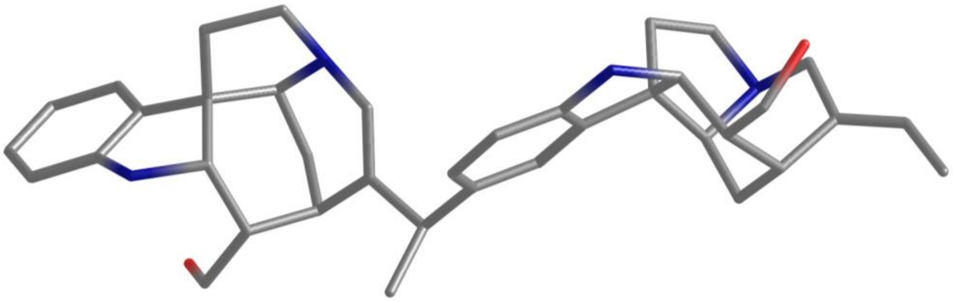

symmetry c1

| | | | |
|---|---|---|---|
| N | -6.045198000 | -1.352007000 | 1.060196000 |
| C | -4.748765000 | -0.817791000 | 0.616791000 |
| C | -3.988242000 | 1.532598000 | -0.098001000 |
| N | -2.767633000 | 1.492646000 | 0.710220000 |
| C | -3.180662000 | 1.588077000 | 2.120048000 |
| C | -4.684839000 | 1.218245000 | 2.148018000 |
| C | -4.990615000 | 0.712037000 | 0.722024000 |
| C | -6.457372000 | 0.797904000 | 0.392293000 |
| C | -7.240823000 | 1.847803000 | -0.040873000 |
| C | -8.605896000 | 1.637642000 | -0.248518000 |
| C | -9.160582000 | 0.387728000 | -0.009314000 |
| C | -8.381979000 | -0.679234000 | 0.435603000 |
| C | -7.026767000 | -0.460228000 | 0.631837000 |
| C | -3.738672000 | 1.027824000 | -1.500365000 |
| C | -3.231519000 | -0.413063000 | -1.404778000 |
| C | -4.311108000 | -1.333737000 | -0.784799000 |
| C | -5.417109000 | -1.701064000 | -1.783965000 |
| C | -1.294996000 | -2.787900000 | -1.136941000 |
| C | -0.829723000 | -1.331947000 | -1.006802000 |
| C | -1.954526000 | -0.401682000 | -0.586675000 |
| C | -1.835937000 | 0.491997000 | 0.401943000 |
| N | 3.804093000 | -0.517658000 | 2.173158000 |
| C | 4.739485000 | 0.052698000 | 1.185766000 |
| C | 4.359212000 | 0.658960000 | -1.330614000 |
| N | 5.158114000 | 1.881804000 | -1.437377000 |
| C | 4.276743000 | 2.881617000 | -0.826451000 |
| C | 3.524913000 | 2.169476000 | 0.335488000 |
| C | 3.782961000 | 0.654406000 | 0.104202000 |
| C | 2.553970000 | -0.203922000 | 0.281721000 |
| C | 1.445524000 | -0.361392000 | -0.525560000 |
| C | 0.400257000 | -1.203405000 | -0.134885000 |
| C | 0.506490000 | -1.866664000 | 1.084567000 |

| | | | |
|---|---|---|---|
| C | 1.611700000 | -1.711080000 | 1.916939000 |
| C | 2.633712000 | -0.870574000 | 1.503283000 |
| C | 5.133502000 | -0.581136000 | -1.708115000 |
| C | 6.300944000 | -0.736502000 | -0.736058000 |
| C | 5.722955000 | -1.015484000 | 0.668850000 |
| C | 6.819102000 | -1.289905000 | 1.698546000 |
| C | 9.391769000 | -0.686761000 | -1.221873000 |
| C | 8.472774000 | 0.491580000 | -1.117038000 |
| C | 7.162286000 | 0.499882000 | -0.867421000 |
| C | 6.436576000 | 1.815172000 | -0.710164000 |
| H | -6.214566000 | -2.332503000 | 0.895058000 |
| H | -3.973239000 | -1.110519000 | 1.322955000 |
| H | -4.337995000 | 2.568940000 | -0.124423000 |
| H | -2.578064000 | 0.915208000 | 2.725768000 |
| H | -3.031250000 | 2.599993000 | 2.493761000 |
| H | -5.288108000 | 2.102363000 | 2.347758000 |
| H | -4.927149000 | 0.471521000 | 2.901215000 |
| H | -6.804081000 | 2.819944000 | -0.229049000 |
| H | -9.229062000 | 2.445847000 | -0.601204000 |
| H | -10.216459000 | 0.232093000 | -0.179601000 |
| H | -8.820848000 | -1.651677000 | 0.609185000 |
| H | -2.982695000 | 1.651285000 | -1.977838000 |
| H | -4.652306000 | 1.091164000 | -2.083407000 |
| H | -3.010934000 | -0.787397000 | -2.407644000 |
| H | -3.842812000 | -2.300979000 | -0.585679000 |
| H | -4.937317000 | -2.249053000 | -2.595353000 |
| H | -6.123048000 | -2.390779000 | -1.312564000 |
| H | -0.466414000 | -3.418631000 | -1.454838000 |
| H | -1.673610000 | -3.172052000 | -0.190307000 |
| H | -2.089535000 | -2.879173000 | -1.876134000 |
| H | -0.533580000 | -1.015866000 | -2.013946000 |
| H | -0.950002000 | 0.523113000 | 1.022865000 |
| H | 4.188724000 | -1.205101000 | 2.803864000 |
| H | 5.310380000 | 0.839950000 | 1.669748000 |
| H | 3.515153000 | 0.786181000 | -2.013202000 |
| H | 3.562784000 | 3.217308000 | -1.577404000 |
| H | 4.850305000 | 3.746301000 | -0.500428000 |
| H | 3.884082000 | 2.479277000 | 1.315050000 |
| H | 2.460752000 | 2.390736000 | 0.294513000 |
| H | 1.370734000 | 0.170118000 | -1.467723000 |
| H | -0.295107000 | -2.517423000 | 1.407105000 |
| H | 1.668388000 | -2.234342000 | 2.861371000 |
| H | 5.494378000 | -0.490577000 | -2.732021000 |
| H | 4.478285000 | -1.451715000 | -1.653654000 |

| | | | |
|---|---|---|---|
| H | 6.883043000 | -1.612260000 | -1.014957000 |
| H | 5.156052000 | -1.946902000 | 0.562782000 |
| H | 7.487320000 | -2.065361000 | 1.310372000 |
| H | 6.385582000 | -1.675524000 | 2.621641000 |
| H | 8.894842000 | -1.637144000 | -1.051849000 |
| H | 9.859306000 | -0.718673000 | -2.206314000 |
| H | 10.200267000 | -0.597540000 | -0.495010000 |
| H | 8.954232000 | 1.455620000 | -1.249990000 |
| H | 7.070213000 | 2.625501000 | -1.068082000 |
| H | 6.283784000 | 1.994603000 | 0.363266000 |
| H | -6.665503000 | -0.198006000 | -1.761898000 |
| H | 7.988092000 | 0.210113000 | 1.286214000 |
| O | -6.120384000 | -0.646877000 | -2.415586000 |
| O | 7.555746000 | -0.138690000 | 2.072386000 |

Compound: **16e**

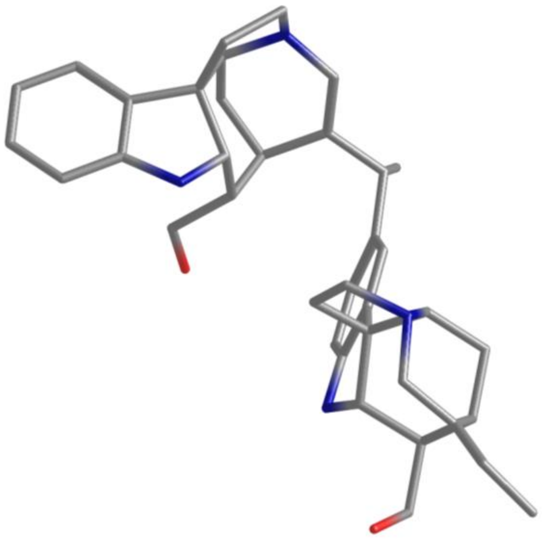

symmetry c1

| | | | |
|---|---|---|---|
| N | 2.346652000 | -2.010687000 | 0.000386000 |
| C | 2.563730000 | -0.602186000 | -0.372205000 |
| C | 4.699862000 | 0.728949000 | -0.959789000 |
| N | 4.136888000 | 1.377630000 | -2.147388000 |
| C | 4.043038000 | 0.334648000 | -3.180373000 |
| C | 4.022331000 | -1.021120000 | -2.427282000 |
| C | 4.015860000 | -0.645565000 | -0.927431000 |
| C | 4.618604000 | -1.726914000 | -0.067561000 |
| C | 5.930210000 | -2.042186000 | 0.218656000 |
| C | 6.202888000 | -3.147824000 | 1.026853000 |
| C | 5.161322000 | -3.920223000 | 1.521830000 |
| C | 3.832706000 | -3.615937000 | 1.232400000 |
| C | 3.576090000 | -2.511352000 | 0.436300000 |
| C | 4.497365000 | 1.569200000 | 0.282469000 |
| C | 2.994756000 | 1.755529000 | 0.489906000 |
| C | 2.338656000 | 0.386706000 | 0.796151000 |
| C | 2.743124000 | -0.113938000 | 2.185253000 |
| C | 1.727316000 | 4.705355000 | -0.021881000 |
| C | 1.267139000 | 3.372829000 | -0.616498000 |
| C | 2.426981000 | 2.405834000 | -0.752990000 |
| C | 2.982776000 | 2.147078000 | -1.939200000 |
| N | -2.539345000 | 0.231578000 | 2.166303000 |
| C | -3.342916000 | -0.321327000 | 1.060452000 |
| C | -3.097775000 | 0.007559000 | -1.522447000 |
| N | -3.437243000 | -1.337304000 | -1.992341000 |
| C | -2.199726000 | -2.080151000 | -1.736731000 |
| C | -1.586925000 | -1.497621000 | -0.429028000 |

| | | | |
|---|---|---|---|
| C | -2.382762000 | -0.189272000 | -0.166335000 |
| C | -1.506885000 | 0.962543000 | 0.260458000 |
| C | -0.614465000 | 1.722156000 | -0.461513000 |
| C | 0.156614000 | 2.704687000 | 0.172996000 |
| C | -0.026404000 | 2.911545000 | 1.534414000 |
| C | -0.919986000 | 2.143775000 | 2.285710000 |
| C | -1.648418000 | 1.159171000 | 1.637317000 |
| C | -4.290212000 | 0.932703000 | -1.482903000 |
| C | -5.306699000 | 0.368264000 | -0.492669000 |
| C | -4.684856000 | 0.422507000 | 0.920082000 |
| C | -5.669940000 | -0.023453000 | 2.000705000 |
| C | -8.230770000 | -0.658858000 | -0.916758000 |
| C | -6.961038000 | -1.399657000 | -1.204582000 |
| C | -5.705311000 | -1.003017000 | -0.991584000 |
| C | -4.562330000 | -1.951268000 | -1.267272000 |
| H | 1.551093000 | -2.196038000 | 0.593636000 |
| H | 1.873470000 | -0.324857000 | -1.169014000 |
| H | 5.766309000 | 0.571496000 | -1.145098000 |
| H | 3.153427000 | 0.492733000 | -3.784934000 |
| H | 4.908986000 | 0.385355000 | -3.838671000 |
| H | 4.922191000 | -1.590476000 | -2.652520000 |
| H | 3.165366000 | -1.639686000 | -2.685543000 |
| H | 6.740925000 | -1.440454000 | -0.171683000 |
| H | 7.224537000 | -3.400853000 | 1.269016000 |
| H | 5.381072000 | -4.772344000 | 2.149607000 |
| H | 3.026783000 | -4.218410000 | 1.627846000 |
| H | 4.978946000 | 2.537887000 | 0.151255000 |
| H | 4.971460000 | 1.079856000 | 1.131975000 |
| H | 2.817488000 | 2.399173000 | 1.351549000 |
| H | 1.255406000 | 0.529012000 | 0.855974000 |
| H | 2.298064000 | -1.092751000 | 2.377297000 |
| H | 3.820018000 | -0.227185000 | 2.294014000 |
| H | 0.881879000 | 5.370082000 | 0.154675000 |
| H | 2.248817000 | 4.564709000 | 0.924510000 |
| H | 2.417714000 | 5.192245000 | -0.707449000 |
| H | 0.887314000 | 3.569868000 | -1.621741000 |
| H | 2.575975000 | 2.579454000 | -2.846848000 |
| H | -3.041154000 | 0.499023000 | 2.999733000 |
| H | -3.547619000 | -1.366263000 | 1.275278000 |
| H | -2.353863000 | 0.397096000 | -2.222271000 |
| H | -1.516863000 | -1.914385000 | -2.568923000 |
| H | -2.401064000 | -3.147131000 | -1.677597000 |
| H | -1.669545000 | -2.181868000 | 0.413660000 |
| H | -0.529963000 | -1.279251000 | -0.573512000 |

| | | | |
|---|---|---|---|
| H | -0.466353000 | 1.541990000 | -1.521344000 |
| H | 0.550962000 | 3.671029000 | 2.043532000 |
| H | -1.039084000 | 2.319497000 | 3.346264000 |
| H | -4.725499000 | 1.011962000 | -2.478407000 |
| H | -3.970915000 | 1.927993000 | -1.170583000 |
| H | -6.182541000 | 1.013244000 | -0.474588000 |
| H | -4.482540000 | 1.483709000 | 1.101734000 |
| H | -5.279578000 | 0.211581000 | 2.991147000 |
| H | -6.604252000 | 0.534996000 | 1.884413000 |
| H | -8.801882000 | -0.510969000 | -1.833634000 |
| H | -8.862264000 | -1.243784000 | -0.246643000 |
| H | -8.068277000 | 0.311962000 | -0.458329000 |
| H | -7.099214000 | -2.390331000 | -1.626739000 |
| H | -4.922467000 | -2.793750000 | -1.856040000 |
| H | -4.225526000 | -2.368134000 | -0.307847000 |
| H | 1.413640000 | 1.030884000 | 3.030023000 |
| H | -6.308867000 | -1.657825000 | 1.160083000 |
| O | 2.336195000 | 0.803824000 | 3.189757000 |
| O | -5.919303000 | -1.418028000 | 2.007053000 |

Compound: **16f**

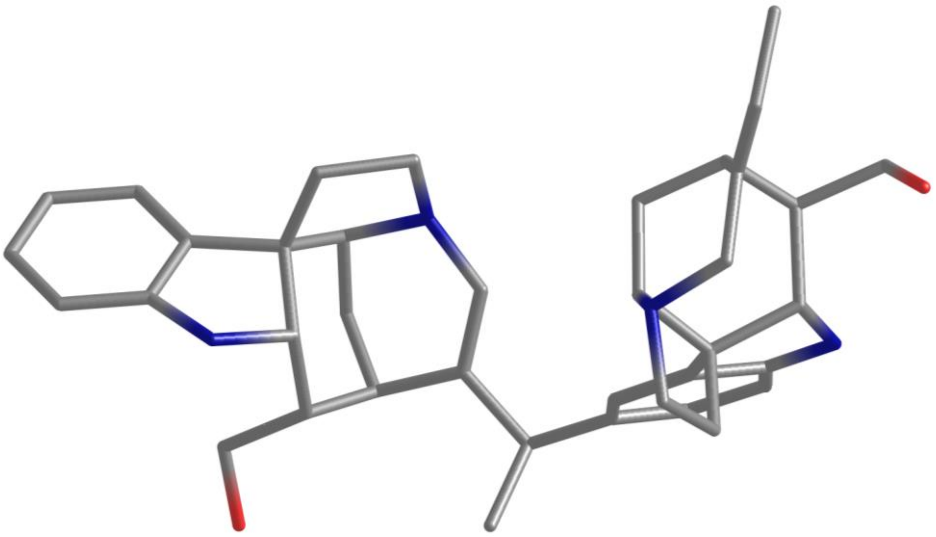

symmetry c1

| | | | |
|---|---|---|---|
| N | 4.196743000 | -1.292972000 | 1.667448000 |
| C | 3.463845000 | -0.431676000 | 0.725447000 |
| C | 3.530943000 | -0.220675000 | -1.843885000 |
| N | 2.076541000 | -0.220000000 | -2.008793000 |
| C | 1.623665000 | -1.594275000 | -1.742004000 |
| C | 2.745190000 | -2.260542000 | -0.904879000 |
| C | 3.763742000 | -1.130526000 | -0.630433000 |
| C | 5.147495000 | -1.672181000 | -0.379986000 |
| C | 6.133955000 | -2.095921000 | -1.244730000 |
| C | 7.323127000 | -2.608307000 | -0.721049000 |
| C | 7.494579000 | -2.697404000 | 0.653257000 |
| C | 6.500805000 | -2.280170000 | 1.536779000 |
| C | 5.330226000 | -1.764850000 | 1.003989000 |
| C | 4.064295000 | 1.188156000 | -1.703211000 |
| C | 3.419816000 | 1.816737000 | -0.467261000 |
| C | 3.881723000 | 1.058598000 | 0.799748000 |
| C | 5.371386000 | 1.294081000 | 1.075665000 |
| C | 1.462677000 | 3.316329000 | 1.333979000 |
| C | 1.118294000 | 2.977339000 | -0.123799000 |
| C | 1.915053000 | 1.794950000 | -0.648775000 |
| C | 1.369860000 | 0.797546000 | -1.353302000 |
| N | -4.484038000 | 2.217806000 | -0.654090000 |
| C | -4.650775000 | 0.885705000 | -0.042766000 |
| C | -2.888913000 | -0.643698000 | 1.140735000 |
| N | -3.706677000 | -1.221333000 | 2.209080000 |
| C | -3.755231000 | -0.129228000 | 3.186277000 |
| C | -3.798255000 | 1.199545000 | 2.376447000 |

| | | | |
|---|---|---|---|
| C | -3.425628000 | 0.790182000 | 0.924830000 |
| C | -2.443923000 | 1.721384000 | 0.256066000 |
| C | -1.088986000 | 1.879746000 | 0.450758000 |
| C | -0.375238000 | 2.821351000 | -0.301358000 |
| C | -1.068911000 | 3.590826000 | -1.226861000 |
| C | -2.443163000 | 3.458143000 | -1.423228000 |
| C | -3.122198000 | 2.514258000 | -0.671868000 |
| C | -2.853520000 | -1.500373000 | -0.102002000 |
| C | -4.274561000 | -1.615209000 | -0.649822000 |
| C | -4.723682000 | -0.214285000 | -1.120466000 |
| C | -6.105320000 | -0.242233000 | -1.774803000 |
| C | -6.132086000 | -4.107388000 | -1.005643000 |
| C | -5.872274000 | -3.345434000 | 0.257649000 |
| C | -5.120905000 | -2.256316000 | 0.427418000 |
| C | -5.067179000 | -1.583303000 | 1.778307000 |
| H | 4.328777000 | -0.933039000 | 2.600664000 |
| H | 2.396927000 | -0.483666000 | 0.937367000 |
| H | 3.965290000 | -0.706742000 | -2.722297000 |
| H | 0.669801000 | -1.572342000 | -1.220170000 |
| H | 1.482101000 | -2.131356000 | -2.678716000 |
| H | 3.229371000 | -3.046806000 | -1.481196000 |
| H | 2.382640000 | -2.702615000 | 0.020700000 |
| H | 5.994499000 | -2.030587000 | -2.316231000 |
| H | 8.109686000 | -2.934649000 | -1.385306000 |
| H | 8.418673000 | -3.093354000 | 1.050496000 |
| H | 6.645231000 | -2.347110000 | 2.606063000 |
| H | 3.808501000 | 1.764207000 | -2.591861000 |
| H | 5.151190000 | 1.158312000 | -1.633809000 |
| H | 3.734276000 | 2.858449000 | -0.375806000 |
| H | 3.361769000 | 1.481236000 | 1.661527000 |
| H | 5.667824000 | 0.771088000 | 1.982330000 |
| H | 6.006762000 | 0.917816000 | 0.272738000 |
| H | 0.896583000 | 4.189908000 | 1.652912000 |
| H | 1.213862000 | 2.489381000 | 1.999064000 |
| H | 2.522248000 | 3.537602000 | 1.453209000 |
| H | 1.420502000 | 3.844850000 | -0.721125000 |
| H | 0.299431000 | 0.738823000 | -1.501601000 |
| H | -4.991256000 | 2.378823000 | -1.511547000 |
| H | -5.580982000 | 0.879630000 | 0.518093000 |
| H | -1.878910000 | -0.548251000 | 1.548028000 |
| H | -2.848964000 | -0.165247000 | 3.789449000 |
| H | -4.605443000 | -0.253092000 | 3.853149000 |
| H | -4.777474000 | 1.673461000 | 2.406796000 |
| H | -3.079653000 | 1.912802000 | 2.773744000 |

| | | | |
|---|---|---|---|
| H | -0.563413000 | 1.262157000 | 1.170090000 |
| H | -0.523211000 | 4.314979000 | -1.818288000 |
| H | -2.959448000 | 4.073994000 | -2.146401000 |
| H | -2.452521000 | -2.484118000 | 0.140060000 |
| H | -2.199931000 | -1.038604000 | -0.844291000 |
| H | -4.267186000 | -2.254283000 | -1.530189000 |
| H | -4.012453000 | 0.062065000 | -1.906538000 |
| H | -6.119109000 | -1.017516000 | -2.547563000 |
| H | -6.309979000 | 0.706846000 | -2.270861000 |
| H | -5.796611000 | -5.139775000 | -0.903757000 |
| H | -7.203525000 | -4.145650000 | -1.206774000 |
| H | -5.645235000 | -3.677186000 | -1.875738000 |
| H | -6.379581000 | -3.734698000 | 1.135116000 |
| H | -5.494737000 | -2.243280000 | 2.531787000 |
| H | -5.713457000 | -0.695088000 | 1.743187000 |
| H | 5.702475000 | 3.130683000 | 0.487460000 |
| H | -7.050000000 | -1.288327000 | -0.433477000 |
| O | 5.640279000 | 2.666907000 | 1.324146000 |
| O | -7.169389000 | -0.433098000 | -0.858813000 |

Compound: **17a**

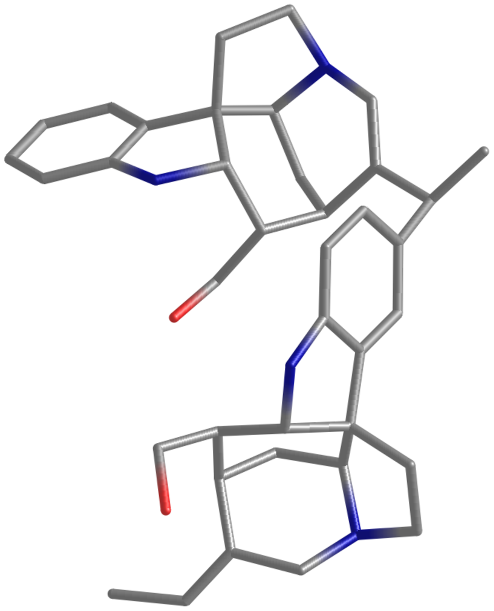

symmetry c1

| | | | |
|---|---|---|---|
| N | 2.363404000 | 1.530000000 | 1.342671000 |
| C | 2.681937000 | 0.252509000 | 0.649435000 |
| C | 4.452567000 | -0.375931000 | -1.171519000 |
| N | 4.870618000 | -1.681137000 | -0.663024000 |
| C | 5.739254000 | -1.384269000 | 0.476889000 |
| C | 5.197974000 | -0.072375000 | 1.099997000 |
| C | 4.129541000 | 0.448217000 | 0.095477000 |
| C | 4.196715000 | 1.950910000 | -0.025766000 |
| C | 5.080814000 | 2.760852000 | -0.713048000 |
| C | 4.984699000 | 4.144161000 | -0.568087000 |
| C | 4.021775000 | 4.696867000 | 0.265709000 |
| C | 3.121960000 | 3.886881000 | 0.953837000 |
| C | 3.222357000 | 2.516796000 | 0.792470000 |
| C | 3.317483000 | -0.529400000 | -2.163452000 |
| C | 2.118917000 | -1.109648000 | -1.412674000 |
| C | 1.606258000 | -0.051007000 | -0.412265000 |
| C | 1.078920000 | 1.191513000 | -1.143518000 |
| C | 2.023583000 | -4.810090000 | -0.032219000 |
| C | 1.491840000 | -3.484432000 | -0.570671000 |
| C | 2.547790000 | -2.412580000 | -0.760559000 |
| C | 3.826265000 | -2.586677000 | -0.414046000 |
| N | -2.667922000 | -0.979442000 | 2.361540000 |
| C | -3.465653000 | -0.430352000 | 1.247161000 |
| C | -3.300671000 | -0.866943000 | -1.322993000 |
| N | -4.725703000 | -0.887550000 | -1.659772000 |

| | | | |
|---|---|---|---|
| C | -5.135111000 | -2.222910000 | -1.211780000 |
| C | -4.286290000 | -2.561904000 | 0.050578000 |
| C | -3.198421000 | -1.456594000 | 0.100111000 |
| C | -1.822544000 | -1.952294000 | 0.472603000 |
| C | -0.866634000 | -2.606869000 | -0.279623000 |
| C | 0.364904000 | -2.937846000 | 0.287898000 |
| C | 0.583614000 | -2.640816000 | 1.630548000 |
| C | -0.371436000 | -1.993721000 | 2.406624000 |
| C | -1.570531000 | -1.634519000 | 1.806197000 |
| C | -2.670760000 | 0.493906000 | -1.483941000 |
| C | -3.363732000 | 1.461174000 | -0.527436000 |
| C | -3.048073000 | 1.015196000 | 0.917777000 |
| C | -3.597204000 | 1.994678000 | 1.952967000 |
| C | -5.057994000 | 4.030964000 | -1.084626000 |
| C | -5.515635000 | 2.607817000 | -1.182691000 |
| C | -4.827097000 | 1.498530000 | -0.907776000 |
| C | -5.514511000 | 0.154269000 | -0.981131000 |
| H | 2.416314000 | 1.469950000 | 2.350318000 |
| H | 2.666464000 | -0.560066000 | 1.372461000 |
| H | 5.323499000 | 0.075059000 | -1.654284000 |
| H | 5.751213000 | -2.219741000 | 1.171561000 |
| H | 6.755164000 | -1.230324000 | 0.115617000 |
| H | 5.999585000 | 0.653975000 | 1.214263000 |
| H | 4.758552000 | -0.230029000 | 2.083728000 |
| H | 5.846642000 | 2.333742000 | -1.347222000 |
| H | 5.664654000 | 4.789479000 | -1.104926000 |
| H | 3.958329000 | 5.770341000 | 0.372175000 |
| H | 2.356136000 | 4.315186000 | 1.585278000 |
| H | 3.633187000 | -1.202733000 | -2.959778000 |
| H | 3.085251000 | 0.432593000 | -2.617532000 |
| H | 1.305870000 | -1.322716000 | -2.111889000 |
| H | 0.741306000 | -0.448877000 | 0.121205000 |
| H | 1.889382000 | 1.848646000 | -1.468342000 |
| H | 0.541020000 | 0.872386000 | -2.038453000 |
| H | 1.209782000 | -5.528729000 | 0.041261000 |
| H | 2.789827000 | -5.221787000 | -0.687350000 |
| H | 2.454263000 | -4.694266000 | 0.961970000 |
| H | 1.061986000 | -3.680607000 | -1.558275000 |
| H | 4.162019000 | -3.501844000 | 0.055161000 |
| H | -2.473705000 | -0.343830000 | 3.120984000 |
| H | -4.511538000 | -0.439433000 | 1.539425000 |
| H | -2.819278000 | -1.578943000 | -1.999023000 |
| H | -4.915670000 | -2.936949000 | -2.004564000 |
| H | -6.206685000 | -2.249582000 | -1.027407000 |

| | | | |
|---|---|---|---|
| H | -4.877894000 | -2.568522000 | 0.964021000 |
| H | -3.831737000 | -3.545157000 | -0.050337000 |
| H | -1.048044000 | -2.835692000 | -1.323615000 |
| H | 1.542949000 | -2.880832000 | 2.072586000 |
| H | -0.175560000 | -1.758738000 | 3.443799000 |
| H | -2.774399000 | 0.830869000 | -2.515190000 |
| H | -1.609215000 | 0.443208000 | -1.240366000 |
| H | -2.925901000 | 2.449356000 | -0.649951000 |
| H | -1.955601000 | 1.060025000 | 0.984656000 |
| H | -3.264931000 | 3.005943000 | 1.696923000 |
| H | -3.197656000 | 1.769423000 | 2.942245000 |
| H | -4.033814000 | 4.129069000 | -0.737696000 |
| H | -5.137344000 | 4.524871000 | -2.053389000 |
| H | -5.699212000 | 4.585604000 | -0.397927000 |
| H | -6.544792000 | 2.482233000 | -1.505451000 |
| H | -6.460515000 | 0.259188000 | -1.511046000 |
| H | -5.769011000 | -0.154908000 | 0.041913000 |
| H | 0.598232000 | 2.052158000 | 0.494702000 |
| H | -5.398663000 | 2.171715000 | 1.238951000 |
| O | 0.154961000 | 1.924772000 | -0.355288000 |
| O | -5.008017000 | 1.954202000 | 2.091558000 |

Compound: **17b**

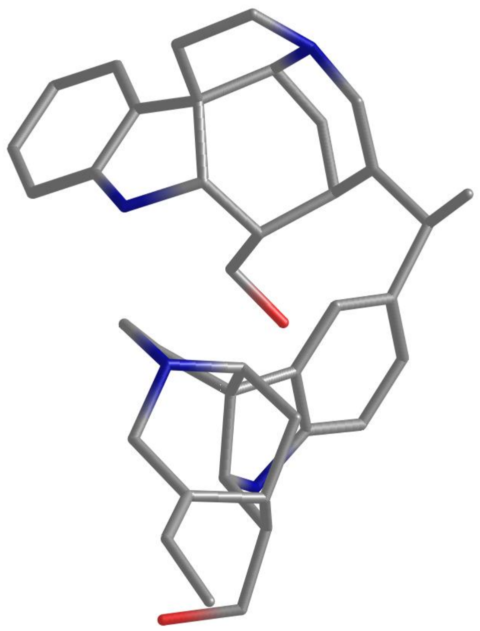

symmetry c1

| | | | |
|---|---|---|---|
| N | 2.356298000 | -1.941593000 | -0.215837000 |
| C | 2.572289000 | -0.493428000 | -0.379282000 |
| C | 4.698287000 | 0.909854000 | -0.800447000 |
| N | 4.113453000 | 1.726149000 | -1.866783000 |
| C | 3.990342000 | 0.848231000 | -3.040928000 |
| C | 3.995290000 | -0.604212000 | -2.497377000 |
| C | 4.014020000 | -0.455204000 | -0.958866000 |
| C | 4.628123000 | -1.652392000 | -0.279919000 |
| C | 5.943438000 | -2.008370000 | -0.067664000 |
| C | 6.226099000 | -3.223524000 | 0.559380000 |
| C | 5.190568000 | -4.061417000 | 0.949393000 |
| C | 3.858312000 | -3.715644000 | 0.732768000 |
| C | 3.591810000 | -2.502960000 | 0.117915000 |
| C | 4.519033000 | 1.559509000 | 0.553895000 |
| C | 3.020108000 | 1.713240000 | 0.809854000 |
| C | 2.370098000 | 0.312325000 | 0.925080000 |
| C | 2.799910000 | -0.384537000 | 2.218454000 |
| C | 0.784461000 | 4.331171000 | -1.116957000 |
| C | 1.282296000 | 3.472437000 | 0.041728000 |
| C | 2.433310000 | 2.549800000 | -0.309394000 |
| C | 2.972000000 | 2.470524000 | -1.529328000 |
| N | -2.529996000 | -0.101511000 | 2.239200000 |
| C | -3.308684000 | -0.499919000 | 1.051985000 |
| C | -3.038723000 | 0.220168000 | -1.448173000 |
| N | -3.353960000 | -1.040232000 | -2.123863000 |

| | | | |
|---|---|---|---|
| C | -2.109191000 | -1.798423000 | -1.968397000 |
| C | -1.522090000 | -1.418948000 | -0.577214000 |
| C | -2.336422000 | -0.174135000 | -0.128425000 |
| C | -1.479175000 | 0.909793000 | 0.476838000 |
| C | -0.580513000 | 1.769078000 | -0.115320000 |
| C | 0.168140000 | 2.654071000 | 0.669720000 |
| C | -0.036866000 | 2.658896000 | 2.043322000 |
| C | -0.933518000 | 1.785998000 | 2.663200000 |
| C | -1.644014000 | 0.902977000 | 1.864914000 |
| C | -4.244210000 | 1.114213000 | -1.282501000 |
| C | -5.266342000 | 0.390501000 | -0.408484000 |
| C | -4.664871000 | 0.231011000 | 1.005098000 |
| C | -5.654646000 | -0.400237000 | 1.984540000 |
| C | -8.169053000 | -0.584086000 | -1.050397000 |
| C | -6.884754000 | -1.261266000 | -1.418979000 |
| C | -5.638534000 | -0.890567000 | -1.120633000 |
| C | -4.477494000 | -1.771913000 | -1.515073000 |
| H | 1.572032000 | -2.211692000 | 0.359824000 |
| H | 1.870037000 | -0.099936000 | -1.114831000 |
| H | 5.761118000 | 0.783781000 | -1.026582000 |
| H | 3.081213000 | 1.087828000 | -3.586975000 |
| H | 4.834351000 | 1.001666000 | -3.711794000 |
| H | 4.896213000 | -1.123514000 | -2.819014000 |
| H | 3.139371000 | -1.187632000 | -2.830148000 |
| H | 6.749196000 | -1.355335000 | -0.377551000 |
| H | 7.250833000 | -3.511252000 | 0.742560000 |
| H | 5.418255000 | -4.999140000 | 1.436556000 |
| H | 3.057080000 | -4.370529000 | 1.045767000 |
| H | 5.001018000 | 2.536604000 | 0.556722000 |
| H | 5.005577000 | 0.952254000 | 1.315594000 |
| H | 2.857738000 | 2.226862000 | 1.758021000 |
| H | 1.288320000 | 0.438720000 | 1.026297000 |
| H | 2.361833000 | -1.383638000 | 2.271515000 |
| H | 3.878639000 | -0.505756000 | 2.293392000 |
| H | -0.028519000 | 4.971374000 | -0.780102000 |
| H | 1.581750000 | 4.962717000 | -1.505457000 |
| H | 0.410454000 | 3.719766000 | -1.937727000 |
| H | 1.646946000 | 4.148580000 | 0.821862000 |
| H | 2.573087000 | 3.037080000 | -2.360607000 |
| H | -3.048971000 | 0.038722000 | 3.092895000 |
| H | -3.495571000 | -1.568521000 | 1.108349000 |
| H | -2.292348000 | 0.721585000 | -2.069794000 |
| H | -1.418046000 | -1.496440000 | -2.754236000 |
| H | -2.295604000 | -2.864021000 | -2.079063000 |

| | | | |
|---|---|---|---|
| H | -1.608251000 | -2.225562000 | 0.148732000 |
| H | -0.465934000 | -1.170722000 | -0.670023000 |
| H | -0.412171000 | 1.731984000 | -1.186016000 |
| H | 0.538132000 | 3.341115000 | 2.657043000 |
| H | -1.067341000 | 1.801750000 | 3.736323000 |
| H | -4.667353000 | 1.343406000 | -2.259773000 |
| H | -3.942287000 | 2.051955000 | -0.813815000 |
| H | -6.151162000 | 1.014659000 | -0.303662000 |
| H | -4.488458000 | 1.253518000 | 1.356165000 |
| H | -6.599969000 | 0.149959000 | 1.941222000 |
| H | -5.283502000 | -0.316994000 | 3.006329000 |
| H | -8.028541000 | 0.303281000 | -0.440563000 |
| H | -8.718878000 | -0.296822000 | -1.947053000 |
| H | -8.810657000 | -1.271648000 | -0.497816000 |
| H | -7.001923000 | -2.176325000 | -1.991416000 |
| H | -4.815358000 | -2.522977000 | -2.227698000 |
| H | -4.146244000 | -2.321052000 | -0.622485000 |
| H | 1.479119000 | 0.614057000 | 3.243053000 |
| H | -6.248842000 | -1.893563000 | 0.887616000 |
| O | 2.405494000 | 0.373701000 | 3.352804000 |
| O | -5.871739000 | -1.783143000 | 1.766371000 |

Compound: **17c**

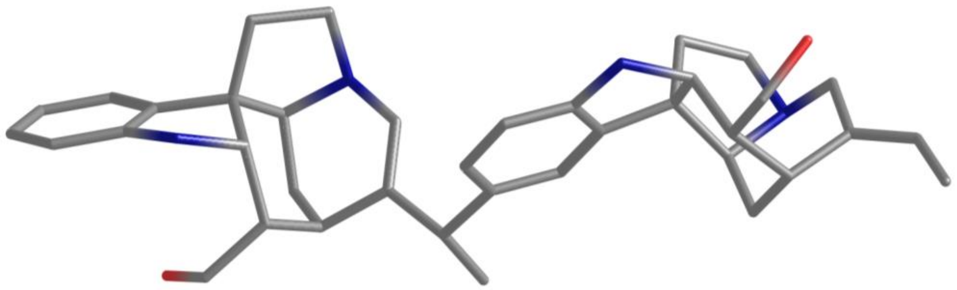

symmetry c1

| | | | |
|---|---|---|---|
| N | 5.362820000 | 1.981430000 | -0.287408000 |
| C | 4.337386000 | 0.931946000 | -0.191877000 |
| C | 4.291544000 | -1.340884000 | 1.041170000 |
| N | 2.950222000 | -1.151068000 | 1.599246000 |
| C | 3.084624000 | -0.140107000 | 2.659840000 |
| C | 4.382317000 | 0.648853000 | 2.344470000 |
| C | 4.874112000 | 0.079749000 | 0.993808000 |
| C | 6.361469000 | 0.244261000 | 0.816291000 |
| C | 7.427545000 | -0.500834000 | 1.276789000 |
| C | 8.727179000 | -0.092536000 | 0.968704000 |
| C | 8.934911000 | 1.055368000 | 0.216227000 |
| C | 7.867362000 | 1.821127000 | -0.249029000 |
| C | 6.581630000 | 1.402503000 | 0.058854000 |
| C | 4.243940000 | -2.054894000 | -0.290493000 |
| C | 3.426028000 | -1.200364000 | -1.257948000 |
| C | 4.108328000 | 0.160757000 | -1.525231000 |
| C | 5.291580000 | 0.053746000 | -2.496803000 |
| C | 0.478324000 | -2.225640000 | -2.153569000 |
| C | 0.885622000 | -0.850792000 | -1.614228000 |
| C | 2.053869000 | -0.984765000 | -0.658184000 |
| C | 1.924010000 | -0.926542000 | 0.669443000 |
| N | -3.399035000 | 2.031881000 | 0.764282000 |
| C | -4.506431000 | 1.061314000 | 0.680973000 |
| C | -4.515700000 | -1.412847000 | -0.165904000 |
| N | -5.446354000 | -2.015960000 | 0.790727000 |
| C | -4.604701000 | -2.182646000 | 1.979793000 |
| C | -3.632166000 | -0.967857000 | 2.018142000 |
| C | -3.761533000 | -0.312342000 | 0.615185000 |
| C | -2.439346000 | 0.095786000 | 0.010428000 |
| C | -1.435856000 | -0.673744000 | -0.551632000 |
| C | -0.269921000 | -0.067974000 | -1.023113000 |
| C | -0.152309000 | 1.317000000 | -0.907283000 |

| | | | |
|---|---|---|---|
| C | -1.145889000 | 2.104238000 | -0.340481000 |
| C | -2.294313000 | 1.475070000 | 0.121576000 |
| C | -5.196870000 | -0.941751000 | -1.428293000 |
| C | -6.204629000 | 0.143964000 | -1.057166000 |
| C | -5.423805000 | 1.363443000 | -0.519805000 |
| C | -6.342267000 | 2.547991000 | -0.219406000 |
| C | -9.319601000 | 0.235557000 | -1.335659000 |
| C | -8.526222000 | -0.459049000 | -0.271649000 |
| C | -7.202737000 | -0.476777000 | -0.104960000 |
| C | -6.608010000 | -1.166132000 | 1.100496000 |
| H | 5.348170000 | 2.560493000 | -1.112984000 |
| H | 3.385844000 | 1.379471000 | 0.088825000 |
| H | 4.872231000 | -1.920290000 | 1.764678000 |
| H | 2.202570000 | 0.495155000 | 2.677740000 |
| H | 3.167756000 | -0.627408000 | 3.630051000 |
| H | 5.133578000 | 0.459759000 | 3.109192000 |
| H | 4.225782000 | 1.724094000 | 2.291308000 |
| H | 7.262315000 | -1.396944000 | 1.860685000 |
| H | 9.570743000 | -0.673360000 | 1.310695000 |
| H | 9.943759000 | 1.359611000 | -0.024102000 |
| H | 8.037863000 | 2.707344000 | -0.843961000 |
| H | 3.761693000 | -3.024120000 | -0.161746000 |
| H | 5.252172000 | -2.219529000 | -0.657898000 |
| H | 3.330954000 | -1.717056000 | -2.215600000 |
| H | 3.392172000 | 0.772971000 | -2.085857000 |
| H | 4.889697000 | -0.304473000 | -3.444376000 |
| H | 5.705316000 | 1.048612000 | -2.685174000 |
| H | -0.331569000 | -2.141808000 | -2.877835000 |
| H | 1.323237000 | -2.707450000 | -2.642397000 |
| H | 0.155573000 | -2.877339000 | -1.342837000 |
| H | 1.252034000 | -0.269033000 | -2.467692000 |
| H | 0.964067000 | -0.711826000 | 1.123834000 |
| H | -3.626000000 | 2.986288000 | 0.527643000 |
| H | -5.094907000 | 1.134327000 | 1.590986000 |
| H | -3.786024000 | -2.188936000 | -0.410898000 |
| H | -4.037377000 | -3.106503000 | 1.874841000 |
| H | -5.220064000 | -2.268382000 | 2.872481000 |
| H | -3.883554000 | -0.257360000 | 2.803288000 |
| H | -2.612072000 | -1.301295000 | 2.195758000 |
| H | -1.557641000 | -1.746417000 | -0.625615000 |
| H | 0.750969000 | 1.791993000 | -1.272183000 |
| H | -1.027932000 | 3.176320000 | -0.265526000 |
| H | -5.695759000 | -1.781286000 | -1.911283000 |
| H | -4.451626000 | -0.544949000 | -2.119198000 |

| | | | |
|---|---|---|---|
| H | -6.721417000 | 0.473048000 | -1.956245000 |
| H | -4.775734000 | 1.673926000 | -1.346709000 |
| H | -5.754449000 | 3.448284000 | -0.038860000 |
| H | -6.968552000 | 2.745338000 | -1.095340000 |
| H | -10.031964000 | 0.925836000 | -0.881688000 |
| H | -8.705627000 | 0.800404000 | -2.030700000 |
| H | -9.904595000 | -0.485628000 | -1.906976000 |
| H | -9.121075000 | -1.000598000 | 0.457542000 |
| H | -7.365475000 | -1.790220000 | 1.572746000 |
| H | -6.340906000 | -0.395800000 | 1.837259000 |
| H | 6.811064000 | -0.515741000 | -1.406425000 |
| H | -7.708872000 | 1.607407000 | 0.796991000 |
| O | 6.332356000 | -0.849485000 | -2.171507000 |
| O | -7.143204000 | 2.373724000 | 0.936356000 |

Compound: **17d**

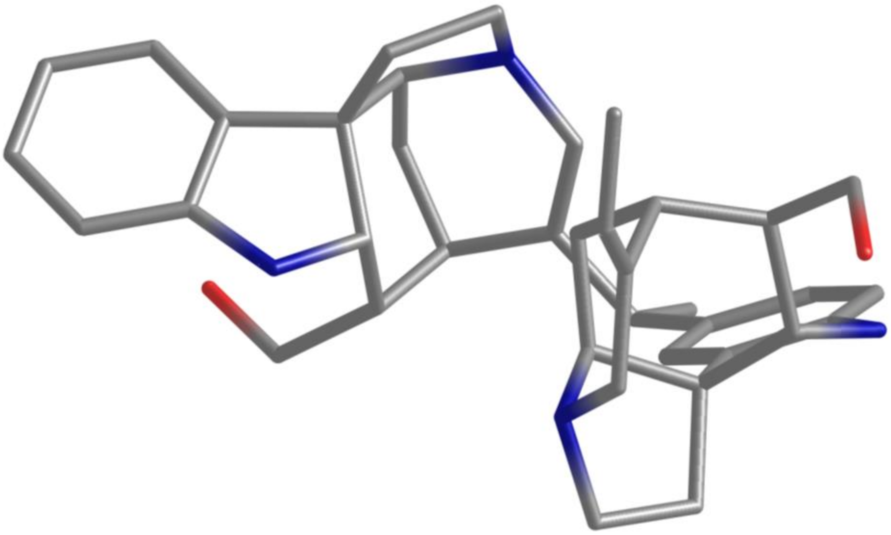

symmetry c1

| | | | |
|---|---|---|---|
| N | 2.612185000 | -1.697655000 | 1.128541000 |
| C | 2.472503000 | -0.412376000 | 0.428893000 |
| C | 3.559901000 | 0.504604000 | -1.742015000 |
| N | 2.320107000 | 1.025982000 | -2.321232000 |
| C | 1.572922000 | -0.150917000 | -2.783389000 |
| C | 2.051678000 | -1.343033000 | -1.911974000 |
| C | 3.108775000 | -0.738216000 | -0.954422000 |
| C | 4.157164000 | -1.745856000 | -0.556804000 |
| C | 5.298016000 | -2.180483000 | -1.199560000 |
| C | 6.088454000 | -3.160560000 | -0.595287000 |
| C | 5.718181000 | -3.691989000 | 0.632463000 |
| C | 4.564855000 | -3.266776000 | 1.289369000 |
| C | 3.791360000 | -2.289018000 | 0.681807000 |
| C | 4.272128000 | 1.558469000 | -0.923715000 |
| C | 3.352941000 | 1.968064000 | 0.225917000 |
| C | 3.101825000 | 0.788758000 | 1.193448000 |
| C | 4.292152000 | 0.499036000 | 2.117471000 |
| C | 1.716517000 | 4.880575000 | 0.088635000 |
| C | 1.244009000 | 3.465962000 | 0.427198000 |
| C | 2.037461000 | 2.435493000 | -0.354549000 |
| C | 1.605900000 | 1.918680000 | -1.507106000 |
| N | -4.243679000 | 2.143530000 | -0.162569000 |
| C | -4.188694000 | 0.708957000 | 0.178112000 |
| C | -2.203659000 | -0.723238000 | 1.102695000 |
| N | -2.923521000 | -1.597604000 | 2.030272000 |
| C | -3.137841000 | -0.717773000 | 3.183977000 |
| C | -3.365765000 | 0.717669000 | 2.624282000 |

| | | | |
|---|---|---|---|
| C | -2.951984000 | 0.628571000 | 1.130450000 |
| C | -2.131850000 | 1.797148000 | 0.643527000 |
| C | -0.806783000 | 2.102205000 | 0.840333000 |
| C | -0.248273000 | 3.255543000 | 0.271068000 |
| C | -1.073658000 | 4.090326000 | -0.468005000 |
| C | -2.426770000 | 3.800908000 | -0.671714000 |
| C | -2.942845000 | 2.643388000 | -0.119316000 |
| C | -2.040079000 | -1.326847000 | -0.271094000 |
| C | -3.429245000 | -1.546418000 | -0.867938000 |
| C | -4.088129000 | -0.165692000 | -1.086952000 |
| C | -5.446246000 | -0.280997000 | -1.779137000 |
| C | -4.882734000 | -4.183745000 | -1.717006000 |
| C | -4.739246000 | -3.635768000 | -0.330301000 |
| C | -4.164738000 | -2.494569000 | 0.053434000 |
| C | -4.211188000 | -2.082672000 | 1.506038000 |
| H | 2.445429000 | -1.698113000 | 2.123200000 |
| H | 1.415376000 | -0.188889000 | 0.292034000 |
| H | 4.192679000 | 0.168450000 | -2.568232000 |
| H | 0.504438000 | 0.032859000 | -2.702842000 |
| H | 1.803036000 | -0.339487000 | -3.830930000 |
| H | 2.514924000 | -2.104359000 | -2.536494000 |
| H | 1.244050000 | -1.816841000 | -1.357438000 |
| H | 5.585773000 | -1.762039000 | -2.155243000 |
| H | 6.991386000 | -3.500508000 | -1.080128000 |
| H | 6.339706000 | -4.444576000 | 1.096565000 |
| H | 4.289679000 | -3.678282000 | 2.250232000 |
| H | 4.475720000 | 2.422964000 | -1.555935000 |
| H | 5.217712000 | 1.170213000 | -0.558572000 |
| H | 3.811155000 | 2.780092000 | 0.796215000 |
| H | 2.327120000 | 1.112148000 | 1.899237000 |
| H | 4.450653000 | 1.394486000 | 2.718160000 |
| H | 4.030116000 | -0.304868000 | 2.811445000 |
| H | 1.199651000 | 5.627795000 | 0.690464000 |
| H | 2.785992000 | 4.972579000 | 0.272762000 |
| H | 1.545020000 | 5.099780000 | -0.964468000 |
| H | 1.475896000 | 3.293322000 | 1.484157000 |
| H | 0.634864000 | 2.190518000 | -1.905200000 |
| H | -4.796229000 | 2.389048000 | -0.970487000 |
| H | -5.099841000 | 0.448021000 | 0.708386000 |
| H | -1.218990000 | -0.552992000 | 1.547301000 |
| H | -2.241486000 | -0.734527000 | 3.802669000 |
| H | -3.966634000 | -1.079069000 | 3.788448000 |
| H | -4.399079000 | 1.044546000 | 2.722955000 |
| H | -2.745305000 | 1.436775000 | 3.154371000 |

| | | | |
|---|---|---|---|
| H | -0.169085000 | 1.443834000 | 1.421436000 |
| H | -0.667486000 | 4.986625000 | -0.915096000 |
| H | -3.047106000 | 4.464315000 | -1.258063000 |
| H | -1.497670000 | -2.269063000 | -0.197040000 |
| H | -1.459947000 | -0.647633000 | -0.899194000 |
| H | -3.327782000 | -2.004738000 | -1.849446000 |
| H | -3.426010000 | 0.357989000 | -1.785328000 |
| H | -5.336809000 | -0.891088000 | -2.681442000 |
| H | -5.794043000 | 0.701528000 | -2.098760000 |
| H | -4.381544000 | -5.148561000 | -1.799226000 |
| H | -5.935257000 | -4.358195000 | -1.944082000 |
| H | -4.481211000 | -3.527233000 | -2.483075000 |
| H | -5.176001000 | -4.252718000 | 0.449059000 |
| H | -4.525175000 | -2.929745000 | 2.114355000 |
| H | -4.990608000 | -1.316464000 | 1.618908000 |
| H | 5.503975000 | -0.623054000 | 1.071689000 |
| H | -6.217861000 | -1.688424000 | -0.679154000 |
| O | 5.545136000 | 0.229248000 | 1.516230000 |
| O | -6.470251000 | -0.799334000 | -0.948262000 |
